# Micro/nano-robotic medical device: preparation, actuation mechanisms and their applications in medicine

**DOI:** 10.1039/d5ra05398f

**Published:** 2025-10-09

**Authors:** Junteng Yao, Yonghui Yang, Meijuan Li, Xue-Bo Chen

**Affiliations:** a School of Electronic and Information Engineering, University of Science and Technology Liaoning Anshan 114051 China yjt10160418@163.com yangyh2636688@163.com 454675273@qq.com xuebochen@126.com

## Abstract

Hydrogels feature a unique three-dimensional crosslinked network integrated with responsive chemical functional groups, enabling them to undergo structural and functional transitions under various external stimuli, including chemical energy, temperature, light, pH, ultrasound, magnetic fields, and ions. These transformations arise from mechanisms such as molecular conformational changes, bond formation or cleavage, and ion exchange. Recent advances in micro/nanorobotics have led to the development of hydrogel-based micro/nano-robotic medical devices that combine excellent biocompatibility with multi-modal actuation, allowing adaptation to diverse environments and precise task execution. This review summarizes current progress in hydrogel micro/nano-robotic medical device research, with a focus on multi-drive synergistic strategies and advantages of composite hydrogel designs. Fabrication techniques, driving mechanisms, and biomedical applications—including targeted drug delivery, tissue engineering, and *in vivo* imaging—are discussed. Finally, the major challenges in clinical translation are analyzed, and possible solutions are proposed to facilitate future practical implementation.

## Abstract 2

Hydrogels, 3D crosslinked networks with responsive functional groups, undergo structural and functional transitions under diverse external stimuli like chemical energy, temperature, light, pH, ultrasound, magnetic fields, and ions.

## Graphical abstract

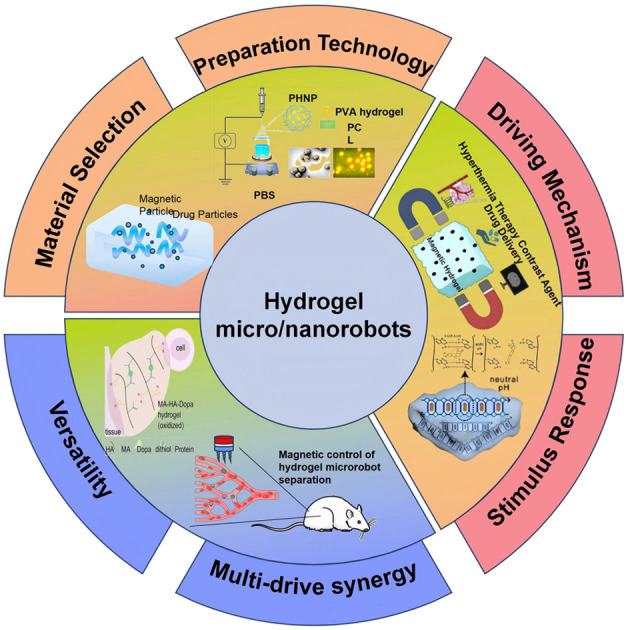

## 1. Introduction

At present, with the deep convergence of nanotechnology, materials science, and biomedical engineering, hydrogel-based micro/nano-robotic medical devices have garnered significant attention from experts across various fields as emerging intelligent diagnostic and therapeutic tools.^1,2^ These devices are capable of performing complex tasks at the micro- to nanoscale, such as targeted drug delivery,^3^ biosensing,^4^ minimally invasive surgical assistance,^5^ and *in vivo* imaging,^6^ offering advantages in addressing traditional medical challenges. However, existing actuation mechanisms still face significant challenges: magnetic field actuation suffers from gradient field attenuation in deep tissues,^7^ pH-responsive mechanisms are susceptible to interference from physiological buffer systems,^8^ and photo-actuation is limited by tissue penetration depth.^9^ More critically, single actuation modes struggle to adapt to the complex demands of dynamic physiological environments. For instance, the drastic pH variations within the gastrointestinal tract, from the stomach to the intestine, can lead to premature drug release from traditional pH-responsive micro/nano-robotic medical devices.^10^ Addressing these issues, recent research has shifted towards multi-actuation synergistic strategies. This involves using magnetic fields to guide micro/nano-robotic medical devices across biological barriers, followed by localized photothermal effects to trigger drug release. This synergistic approach leverages the physical orientation capability of magnetic nanoparticles in a magnetic field, combined with photothermal materials like gold nanoparticles or carbon nanotubes that convert light energy into heat through non-radiative relaxation under specific wavelengths of light. This allows for precise regulation of the hydrogel network structure and drug release kinetics, potentially elevating targeting accuracy to the cellular level.^11^ This ‘external navigation combined with internal response’ closed-loop design offers a novel approach to overcome the limitations of single mechanisms.^8^ Experiments have confirmed that dual-actuation micro/nano-robotic medical devices, utilizing both magnetic and light fields, demonstrate improved tumor drug enrichment efficiency and reduced overall toxicity compared to their single magnetic field-driven counterparts.^7^ Current research predominantly focuses on innovative actuation mechanisms for hydrogel-based micro/nano-robotic medical devices and their *in vitro* or preliminary *in vivo* functional validation. To achieve clinical translation, it is imperative to overcome the critical bottleneck of transitioning from functional demonstration to a safe and effective diagnostic and therapeutic platform. This particularly demands close attention to continuously updated biomedical product regulatory science requirements and emerging clinical trial design strategies worldwide.^12^ These evaluations must be aligned with the latest guidance principles from international regulatory bodies such as the US FDA and European EMA concerning novel medical devices or nanomedicines. This necessitates moving beyond the scope of locomotion and control performance research to systematically evaluate their biomedical behaviors. Firstly, their pharmacokinetic (PK) properties must be elucidated. This includes providing typical dosing metrics, such as drug loading per kilogram of body weight mg per kg or micro/nano-robotic medical device particle count per kilogram particles per kg. There is an urgent need to clarify the spatio-temporal distribution, retention, degradation, and clearance patterns of these devices in complex biological environments, for instance, drug concentration–time curves (AUC) and peak concentrations (*C*_max_) in various tissues and blood. Furthermore, the regulatory mechanisms by which their dynamic properties and material matrices influence targeting efficiency and drug release kinetics need to be clarified. Secondly, pharmacodynamic (PD) correlations must be established. It is necessary to quantify the causal relationship between their targeted enrichment and therapeutic efficacy, establish dose-response relationships, and accordingly evaluate and define their therapeutic window. Their advantages in enhancing local therapeutic index and reducing systemic toxicity compared to conventional therapies must also be assessed. Finally, immunogenicity presents a significant clinical challenge that cannot be overlooked. Although hydrogel matrices generally exhibit good biocompatibility, the potential immunogenic risks posed by exogenous functional components, such as magnetic nanoparticles, and their degradation products still require comprehensive evaluation. Actively evading immune recognition through material design and surface modification strategies is crucial for achieving long-term safe application. Therefore, while reviewing the preparation methods, actuation mechanisms, and current medical applications of hydrogel-based micro/nano-robotic medical devices, this paper particularly emphasizes the importance of a research perspective that integrates their actuation performance with pharmacokinetic, pharmacodynamic characteristics, and immunogenicity. It also discusses how to accelerate their clinical translation process through preclinical studies compliant with the latest regulatory science requirements.^13^ This paper aims to provide theoretical references and practical directions for developing the next generation of intelligent diagnostic and therapeutic platforms with both efficient actuation and controllable biomedical functions.

Hydrogels serve as ideal multifunctional materials for constructing hydrogel-based micro/nano-robotic medical devices. They form highly hydrated three-dimensional network structures through physical or chemical crosslinking. Physical crosslinking relies on reversible non-covalent interactions between polymer chains, such as hydrogen bonds, hydrophobic interactions, electrostatic forces, or chain entanglement. Chemical crosslinking, on the other hand, permanently connects polymer chains *via* irreversible covalent bonds, typically involving chemical reactions between multifunctional crosslinkers and specific reactive functional groups on the polymer chains, such as hydroxyl, amine, carboxyl, or vinyl groups. This structure not only mimics the mechanical properties of biological tissues with high biocompatibility, especially for performing delicate operations on biological entities in complex *in vivo* environments. Hydrogels can also be functionalized to respond to environmental variables such as temperature, pH, magnetic fields, and light, typically achieved through chemical modification. For instance, the introduction of acidic or basic functional groups capable of protonation or deprotonation enables pH responsiveness. Embedding photosensitive groups like azobenzene allows for light responsiveness by altering molecular conformation through photoisomerization. Grafting thermosensitive polymer segments such as poly(*N*-isopropylacrylamide) (PNIPAM) enables temperature responsiveness by leveraging its hydrophobic-hydrophilic phase transition. Furthermore, doping with surface-functionalized magnetic nanoparticles imbues the devices with physical orientation and force-response capabilities under a magnetic field. These precise chemical designs are central to determining the actuation modes of hydrogel-based micro/nano-robotic medical devices. Hydrogel types are generally categorized into natural hydrogels, synthetic hydrogels, and composite hydrogels, each possessing distinct characteristics based on their preparation conditions.^14,15^ The fabrication of hydrogel-based micro/nano-robotic medical devices is a complex and intricate process involving various preparation techniques. Emulsification, electrospray ionization, and microfluidic techniques are commonly used for preparing hydrogel microspheres, while photolithography, electrodeposition, inkjet printing, and microextrusion are frequently employed for fabricating hydrogel-based micro/nano-robotic medical devices. These techniques each have their advantages and disadvantages and are often combined. For example, in the preparation of hydrogel microspheres, microfluidic technology excels at batch production of uniformly sized microspheres compared to emulsification and electrospray ionization, but it incurs higher equipment maintenance costs. The resulting hydrogel microspheres can perform simple controlled drug release operations and can also serve as functional materials for photolithography, enabling multi-material composite printing with micron-level resolution through 3D and 4D printing. Notably, the combination of mold casting assistance and electrodeposition techniques has successfully addressed the challenge of oriented deposition of magnetic nanoparticles within the gel network.^16,17^ Currently, the actuation mechanisms of hydrogel-based micro/nano-robotic medical devices are broadly divided into self-response and external actuation, classifying movement types as fuel-driven and field-driven. Self-response mechanisms utilize chemical fuels that react under the action of enzymes or other catalysts, generating local changes in pH, temperature, or gas, which result in random movement. Subsequent specific operations are achieved through molecular conformational changes of environment-sensitive polymer chains within the hydrogel. For instance, pH-responsive hydrogels in the tumor microacidic environment undergo carboxylic group protonation, leading to polymer chain shrinkage, while temperature-responsive materials achieve on-demand deformation through hydrophobic–hydrophilic phase transitions induced by local hyperthermia. On the other hand, external actuation mechanisms enable remote precise control through physical fields such as magnetic, light, and acoustic fields. For example, after magnetic nanoparticles are embedded in a hydrogel, gradient magnetic fields can guide the directional movement of the device, demonstrating good performance in tumor-targeted therapy.^7,18^ Photo-actuation primarily relies on light field parameters such as wavelength, power, and irradiation location, along with asymmetrically distributed material designs, to precisely control movement speed, direction, and drug release location, suitable for non-invasive interventions in deep tissues.^16^ These mechanisms are enhanced through chemical modifications, such as the introduction of thermosensitive polymers or magnetic components, forming a closed-loop control from stimulus to response to functional output.^8^ However, single actuation mechanisms often face challenges such as environmental interference, insufficient response rates, or biocompatibility issues. For instance, as previously mentioned, magnetic field actuation in deep tissues may suffer from reduced precision due to magnetic field attenuation, while pH response in dynamic physiological environments may be weakened by buffering effects, reducing the sensitivity of protonation/deprotonation of ionizable groups within the hydrogel network. To address this, researchers have proposed multi-actuation synergistic strategies that integrate external physical fields with internal biochemical responses to overcome the limitations of single mechanisms. For example, after a micro/nano-robotic medical device is localized by a magnetic field, a photothermal effect can trigger localized drug release, or a pH response can regulate the device's retention time in specific tissues. This multimodal actuation not only improves environmental adaptability but also provides a technical foundation for complex tasks such as theranostics.^11^ In terms of clinical translation, it offers innovative solutions to challenges in traditional medicine such as insufficient precision, high invasiveness, and significant side effects. Its application scenarios cover four main directions: targeted drug delivery, which achieves local therapy through magnetic navigation and stimulus-responsive release mechanisms, such as ferric oxide nanoparticle-hydrogel composites guided by magnetic fields and utilizing their pH sensitivity for tumor DNA detection and drug delivery;^19^ biosensing, which employs the real-time monitoring capabilities of fluorescent hydrogels, detecting disease biomarkers through dynamic fluorescence signal changes to provide molecular-level sensitivity for early diagnosis;^20^ minimally invasive surgical assistance, which leverages miniaturized structures and multimodal actuation to achieve sub-millimeter operational precision in vascular intervention and nerve repair;^21^ and *in vivo* imaging, which integrates imaging, actuation, and drug release functions through ultrasound imaging and multimodal actuation techniques to achieve a closed loop of detection, treatment, and monitoring.^6^

**Fig. 1:**
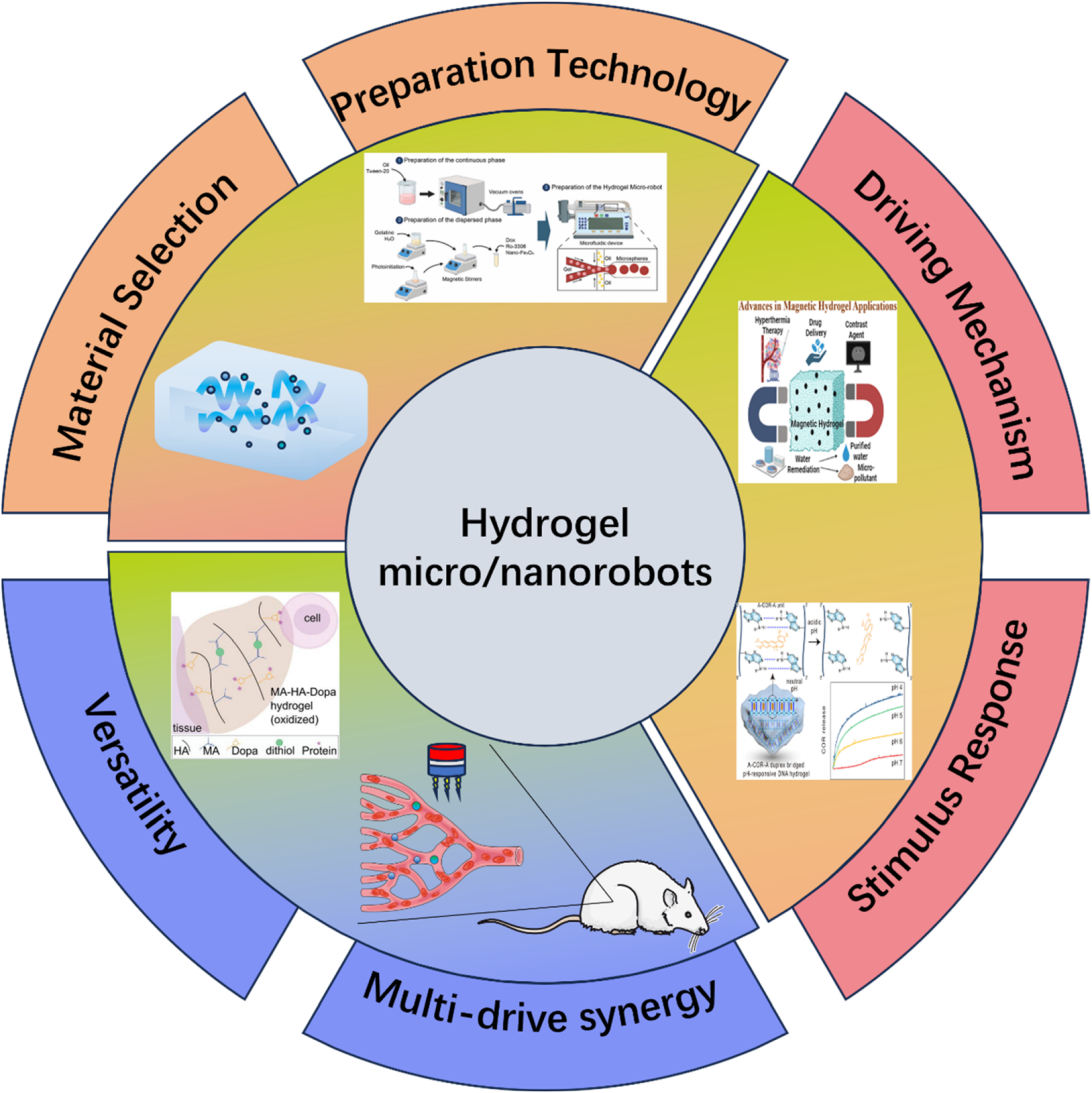
Illustrates composite hydrogel materials containing magnetic particles and specific drugs. One of the fabrication techniques for hydrogel-based micro/nano-robotic medical devices involves constructing hydrogel microspheres within the device using droplet microfluidic technology. Copyright © 2024 Tao, Li, Yang, Yin, Zhang, Wang, Wang, Pu, Wang, Zhang, Mu, Wu, He, and Yang.^18^ The incorporation of superparamagnetic particles, such as Fe_3_O_4_, into hydrogels enables external magnetic field manipulation of the hydrogel-based micro/nano-robotic medical device. Copyright © 2023, American Chemical Society.^22^ Imparting pH responsiveness to DNA hydrogels is typically achieved by introducing pH-sensitive nucleic acid bases into the DNA sequence or by covalently grafting pH-sensitive polymers onto the DNA backbone, allowing the device to control drug release in response to pH stimuli. Copyright © 2024 American Chemical Society.^23^ This includes drug delivery by magnetically controlled hydrogel-based micro/nano-robotic medical devices in *in vivo* experiments in mice, and biochemical reactions involving hydrogel-based micro/nano-robotic medical devices during minimally invasive surgery. © 2020 The Authors. Published by WILEY-VCH Verlag GmbH & Co. KGaA, Weinheim.^21^

This paper emphasizes the development and potential of hydrogel-based micro/nano-robotic medical devices in biomedicine. It analyzes the functionalization and modification methods of hydrogels, starting from material selection and fabrication design strategies, and their impact on the performance of these devices, such as grafting responsive polymer segments or introducing specific functional groups. As depicted in Fig. 1, the initial focus is on composite hydrogel materials incorporating magnetic particles and specific drugs, as well as the construction of hydrogel microspheres within micro/nano-robotic medical devices using droplet microfluidic techniques. Through advanced fabrication methods, we obtain chemically modified composite hydrogels, leading to multifunctional or multi-actuation synergistic hydrogel-based micro/nano-robotic medical devices. Subsequently, this paper explores the principles and applicable scenarios of single actuation mechanisms *versus* multi-actuation synergy in hydrogel-based micro/nano-robotic medical devices. A comparative analysis highlights the biomedical advantages of multifunctional and multi-actuation synergistic hydrogel-based micro/nano-robotic medical devices. For instance, incorporating superparamagnetic particles like Fe_3_O_4_ into hydrogels enables external magnetic field manipulation of these devices, while imparting pH-responsive capabilities to DNA hydrogels (either by introducing pH-sensitive nucleic acid bases into the DNA sequence or by covalently crosslinking pH-sensitive polymers) allows the devices to control drug release in response to pH stimuli. Following this, the discussion transitions from actuation methods to the biomedical applications of hydrogel-based micro/nano-robotic medical devices. This section presents medical applications such as *in vivo* experiments of magnetically controlled hydrogel-based micro/nano-robotic medical devices for drug delivery in mice and large animals (*e.g.*, pigs, dogs), as well as biochemical reaction data obtained from hydrogel-based micro/nano-robotic medical devices during minimally invasive surgery in humans. Finally, the paper discusses the enormous potential of hydrogel-based micro/nano-robotic medical devices in biomedical applications such as precise drug delivery, minimally invasive surgery, biosensing, and tissue engineering. It also addresses the main challenges hindering the development of hydrogels into practical materials and outlines potential future research directions in this field, particularly concerning biosensing visualization and precise drug delivery.

## 2. Preparation of hydrogel-based micro/nano-robotic medical devices

**Fig. 2:**
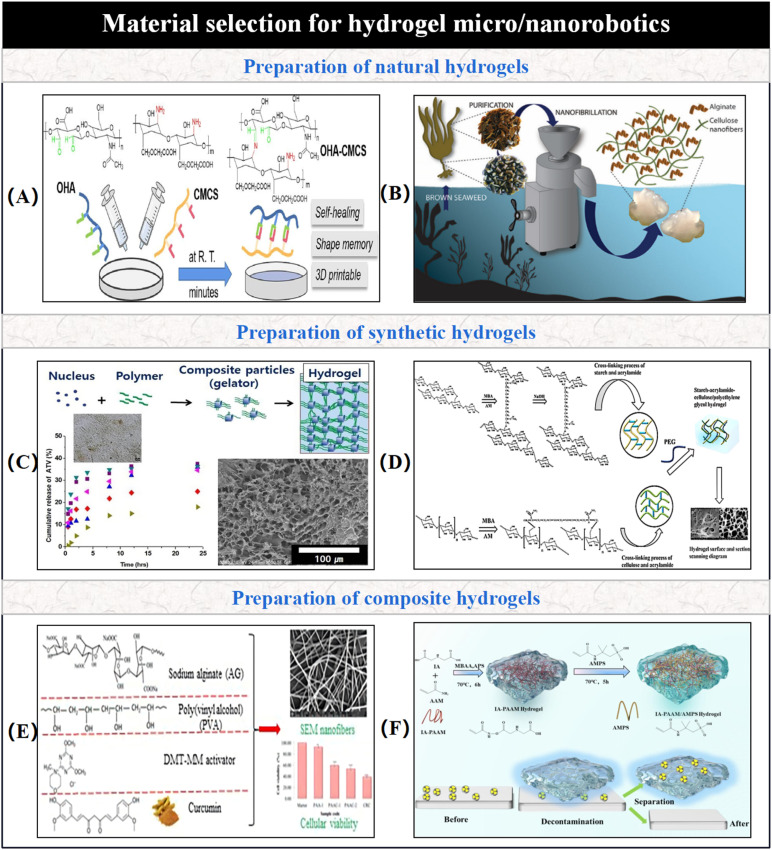
Material selection for hydrogel-based micro/nano-robotic medical devices. (A) Synthesis process, chemical structure, and functional properties of natural polysaccharide-based multifunctional hydrogels constructed *via* Schiff Base Reaction. © 2023 Elsevier Ltd. All rights reserved.^24^ (B) Full-process transformation pathway from natural brown algae to high-value nanomaterials. © 2020 American Chemical Society.^25^ (C) Construction of a smart polyacrylic acid hydrogel system with sustained drug release function using the lipid-lowering drug atorvastatin (ATV) as a crosslinker. © 2020 The Korean Society of Industrial and Engineering Chemistry. Published by Elsevier B.V. All rights reserved.^26^ (D) A synthesis strategy for a green high-performance hydrogel, demonstrated through a three-step crosslinking reaction and final product characterization. © 2021 The Authors. Published by American Chemical Society.^27^ (E) Preparation mechanism of sodium alginate/polyvinyl alcohol/curcumin composite hydrogel (AG-PVA-Cur). © 2025 Wiley Periodicals LLC.^28^ (F) Synthesis pathway and radionuclide decontamination mechanism of a double network hydrogel. © 2025 Published by Elsevier Ltd.^29^

As previously mentioned, hydrogels typically swell or contract by absorbing or releasing water in response to external stimuli. However, simple swelling alone cannot fulfill the functional requirements of micro/nano-robotic medical devices. Therefore, to achieve intelligent shape deformation for functionality, as shown in Fig. 2, traditional hydrogel materials are transformed into smart responsive materials by integrating propulsion, functionality, environmental adaptability, collective behavior, and interoperability through advanced fabrication techniques. In this chapter, we will introduce common fabrication techniques for hydrogel-based micro/nano-robotic medical devices and the design of hydrogel types.

### 2.1. Material selection for hydrogel-based micro/nano-robotic medical devices

#### 2.1.1. Natural and synthetic hydrogels

Natural hydrogels primarily fall into two categories: polysaccharide hydrogels and protein hydrogels. Natural hydrogels are readily prepared from natural sources. Typically, these hydrogels are composed of natural polymer compounds and exhibit excellent biocompatibility and biodegradability. As shown in Fig. 2(B), Berglund *et al.* demonstrated a comprehensive conversion pathway from natural brown algae to high-value nanomaterials. Starting with natural brown algae, they employed dilute acid treatment and water washing to convert it into millimeter-sized light brown fragments, effectively enhancing the efficiency of subsequent reactions. Subsequently, alginate nanofibers were produced through nanofibrillation, retaining their natural anionic properties and acquiring ionic crosslinking capability *via* coordination of their carboxylate groups with divalent cations, which were then used to construct hydrogel networks.^25^ These materials find extensive applications in drug delivery and tissue engineering. Examples such as chitosan, alginate, and hyaluronic acid exhibit good biocompatibility and biodegradability, with some possessing inherent antibacterial properties. The cationic nature of chitosan stems from the abundance of free amine groups (–NH_2_) in its structure. Under physiological pH conditions, these amine groups can be protonated to form ammonium groups (–NH_3_^+^), whose positive charge enables them to disrupt negatively charged bacterial cell membranes *via* electrostatic interactions. Chemical modifications such as sulfation (introducing sulfate groups) and carboxymethylation (introducing carboxyl groups) can enhance antibacterial activity or mechanical properties. As shown in Fig. 2(A), Yu *et al.* prepared OHA-CMCS hydrogel networks with self-healing, shape memory, and 3D printing applicability through Schiff base reaction (condensation of aldehyde and amine groups).^24^ Furthermore, proteins including fibronectin, sericin, collagen, and gelatin mimic the structure and function of the extracellular matrix through their amino acid sequences and spatial conformations, achieving synergistic antibacterial effects when combined with antimicrobial peptides or metallic nanoparticles like Ag and CuO.^30^ Among these, DNA hydrogels are particularly noteworthy. Their potential for molecular programmability and customized biological function arises from the precise molecular self-assembly of DNA sequences *via* specific hydrogen bonding (A–T and G–C base pairing). For example, Li and Chen noted that through DNA sequence design, specific binding of the sequence to target molecules can induce conformational changes or denaturation of DNA strands, thereby achieving intelligent hydrogel responses and dynamic regulation of structure and function.^31^ In the field of biosensing, Bai *et al.* mentioned optimizing the molecular recognition-based responsive sensing performance of DNA hydrogels for rapid detection of cancer biomarkers and pathogens by molecularly regulating aggregation-induced emission (AIE) materials and enhancing their fluorescent properties.^32–34^

Synthetic hydrogels, primarily fabricated through chemical crosslinking—that is, by forming stable covalent bonds to construct their networks—exhibit enhanced mechanical properties. However, during degradation, they tend to display higher toxicity compared to natural hydrogels. The physicochemical and functional attributes of these synthetic hydrogel materials can be finely tuned by modifying their synthesis techniques and the chemical structure and proportion of their constituent monomers, thus enabling a wide range of applications tailored to specific requirements. As shown in Fig. 2(C), Cho *et al.* utilized the lipid-lowering drug atorvastatin (ATV) as a crosslinker. By reacting its reactive functional groups, such as hydroxyl or carboxyl groups, with corresponding groups on the polyacrylic acid chains, they constructed a synthetic hydrogel system with sustained drug release capabilities. This system is suitable for chronic disease treatment requiring long-term drug administration and offers a new paradigm for the development of intelligent drug delivery systems.^26^ Among these, the characteristics of double-network hydrogels are particularly prominent. Saito *et al.* first proposed the double-network structure and subsequently introduced improved designs.^35^ As depicted in Fig. 2(D), Gao *et al.* demonstrated a synthesis strategy for a green, high-performance hydrogel through a three-step crosslinking reaction and final product characterization. The core of this strategy lies in the synergistic action of chemical and physical crosslinking, integrating the enzymatically degradable polysaccharide characteristics of starch, the hydrophilic amide groups of acrylamide, the microcrystalline-enhanced rigidity of cellulose, and the toughening function of the flexible ether bonds of PEG.^27^ Double-network hydrogels consist of two distinct polymer networks, typically comprising a tightly crosslinked rigid network and a loosely crosslinked flexible network. This structure endows the hydrogel with exceptionally high mechanical strength and toughness, enabling it to withstand significant stress and strain. Xu *et al.* proposed a double-network dynamic hydrogel bioink strategy aimed at addressing the application limitations of dynamic hydrogels in biomanufacturing due to mechanical instability, while simultaneously promoting angiogenesis.^36^ As illustrated in Fig. 2(F), the first network (IA-PAAM) is formed by free-radical polymerization using itaconic acid, which possesses acidic carboxyl groups, and acrylamide, with its hydrophilic amide groups, as raw materials. This creates a loosely crosslinked polymer network. The carboxyl groups can undergo protonation or deprotonation with pH changes, influencing the degree of hydration, thereby endowing the first network with high swelling ratios and pH responsiveness, enabling it to closely conform to uneven surfaces. The second network (AMPS) is established through the introduction of sulfonic acid groups. These strongly acidic groups remain ionized even across a broad pH range, providing stable electrostatic repulsion and osmotic pressure. This strengthens the rigid support of the network and enhances its resistance to radiation degradation, thereby achieving efficient and safe radioactive surface decontamination.^29^

**Fig. 3:**
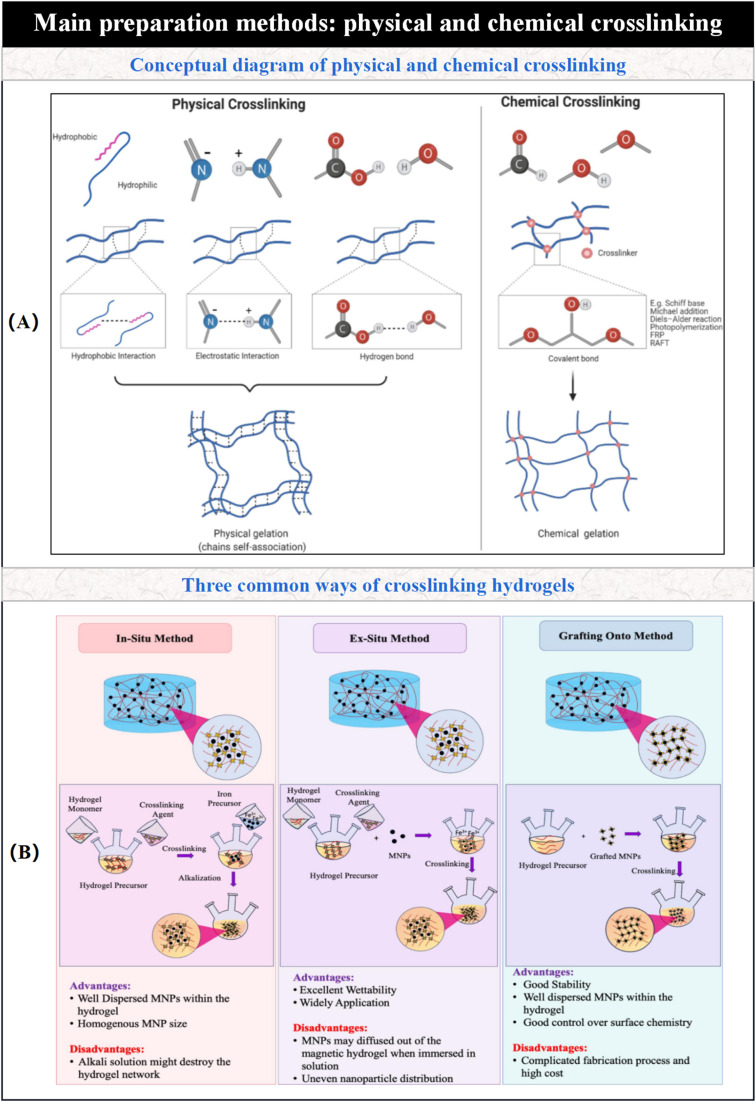
Two crosslinking methods of hydrogels: physical crosslinking and chemical crosslinking. © 2024 The Authors. Published by Elsevier B.V.^37^ (A) Comparison of fabrication pathways for chemical crosslinking (covalent bonds) and physical crosslinking (hydrogen bonds/electrostatic interactions). (B) Preparation strategies for magnetic hydrogels: *in situ* method, non-*in situ* method, and grafting method.

In the preparation of hydrogel-based micro/nano-robotic medical devices, the choice of crosslinking mechanism affects the physicochemical properties of the hydrogel, which in turn influences the performance of the device. As shown in Fig. 3(A) illustrates two fundamental crosslinking methods for hydrogel formation, which determine critical properties such as hydrogel structural stability and reversibility. Physical crosslinking achieves polymer chain crosslinking through non-covalent interactions; chemical crosslinking permanently links polymer chains *via* covalent bonds, relying on crosslinking agents. (B) Addresses “how magnetic nanoparticles are incorporated into hydrogels”, comparing three preparation strategies—*in situ* method, non-*in situ* method, and grafting method—which impact the dispersibility, stability, and application scenarios of magnetic hydrogels.^37^ For applications requiring good biocompatibility and biodegradability, physically crosslinked hydrogels may offer greater advantages. Conversely, for applications demanding higher mechanical strength and structural stability, chemically crosslinked hydrogels or hybrid crosslinking approaches may be necessary to optimize hydrogel performance. Different types of hydrogels possess distinct advantages and disadvantages, as summarized in Table 1.

**Table 1:** Comparison of different types of hydrogels

Typology	Kind	Specificities	Appliance	Reference
Classification according to composition	Protein-based hydrogels	Highly biocompatible, low toxicity, degradable	Tissue regeneration and accelerated wound healing	38
	Polysaccharide-based hydrogel		Antimicrobial, pro-vascular regenerative and injectable	39
	Polyacrylic acid	Strong mechanical properties, low biocompatibility, not easy to degrade and generate toxic substances	Rapid hemostasis for 3D printing	40
	Polyethylene glycol group		Myocardial repair, cartilage repair	41
Classification by structure	DNA hydrogel	Programmability and biodegradability	Fast-repairing, antibacterial	42
			Biosensing	43
	Double mesh hydrogel	Strong mechanical properties, versatility, two meshes can carry different functions	Fast-repairing, antibacterial	44
			Bionic platelets, hemostatic	45
			Simultaneous sensing of temperature and pressure	46
			Fast temperature response, high strength, toughness and expandability	47

#### 2.1.2. Composite hydrogels (stimuli-responsive hydrogels)

Composite hydrogels are hydrogel systems formed by combining multiple materials through physical or chemical means. Compared to single hydrogels, composite hydrogels can significantly improve their physical, chemical, and biological properties, such as mechanical strength, electrical conductivity, and magnetism, through judicious selection and combination of matrix materials. This characteristic enables micro/nano-robotic medical devices constructed from composite hydrogels to respond to multiple stimuli or be controlled by various fields, thereby providing possibilities for the fabrication of multifunctional and multi-actuation synergistic micro/nano-robotic medical devices. As shown in Fig. 1, these include composite hydrogel materials containing magnetic particles and specific drugs. For example, Santhosh *et al.* combined magnetic nanoparticles (SPIONs)-modified reduced graphene oxide (m-rGO) with collagen to form a magneto-responsive composite hydrogel. In this system, m-rGO served as a magnetic carrier, and its surface chemical modification promoted interaction with collagen, leading to guided growth, differentiation, and functionalization of neural cells, offering an innovative strategy for neural tissue engineering.^48^ Magnetic actuation is a common propulsion method for hydrogel-based micro/nano-robotic medical devices. Traditional magnetic hydrogels face bottlenecks such as poor mechanical properties and insufficient magnetic field response, making it difficult to achieve both high magnetic particle content and uniform dispersion simultaneously, while also lacking interfacial adhesion. Hu *et al.* modified Fe_3_O_4_ nanoparticles with TMSPMA silane coupling agent. Through the condensation reaction between silanol groups and hydroxyl groups on the nanoparticle surface, and free radical copolymerization of methacrylate groups with polyacrylamide (PAAm) monomers, Fe_3_O_4_ nanoparticles were covalently embedded into the PAAm hydrogel network. This enhanced the mechanical properties, leading to the preparation of a novel tough and adhesive magnetic hydrogel with high Fe_3_O_4_ content.^49^ As shown in Fig. 2(E), a composite hydrogel prepared from sodium alginate, polyvinyl alcohol, and curcumin features curcumin as the functional core due to its antibacterial and antioxidant properties. The AG-PVA network acts as an enhancing carrier, capable of reducing toxicity and controlling release. DMT-MM catalyzes the formation of stable crosslinks, offering new insights for the development of wound dressings for infected wounds.^28^ As smart materials, composite hydrogels are capable of responding to multiple stimuli. Magnetically-driven pH-responsive composite hydrogels for controlled drug delivery can undergo changes in shape and performance in response to variations in environmental pH, driven by magnetic fields. pH-sensitive hydrogels contract in acidic environments and swell in alkaline environments. This behavior is attributed to the protonation or deprotonation of weak acidic or basic functional groups incorporated into the hydrogel network upon pH changes. These processes alter the charge density, hydrophilicity, and interchain electrostatic interactions of the polymer chains, thereby regulating the hydrogel's swelling degree. This characteristic can be utilized to design intelligent drug release systems.^10^ Furthermore, a pH- and temperature-dual responsive microgel-embedded hydrogel has been developed, exhibiting excellent mechanical properties, adhesive properties, drug loading and release capabilities, and antibacterial properties, which can significantly promote wound healing.^8^

**Fig. 4:**
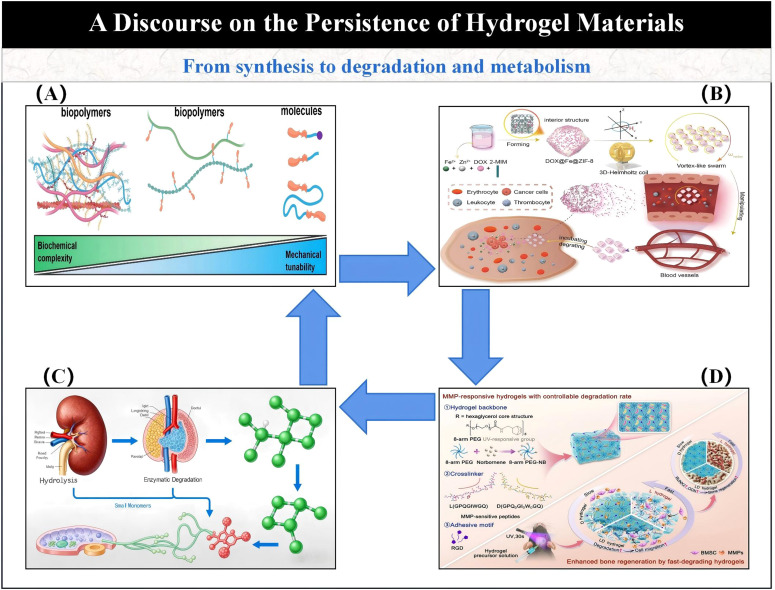
Sustainable cycle of hydrogel materials. (A) Evolution from natural hydrogels to synthetic hydrogels, ultimately forming fully synthetic systems. © The Royal Society of Chemistry 2025.^50^ (B) Process of magnetic micro/nano-robotic medical device swarms in tumor-targeted drug delivery. © Science China Press 2024.^51^ (C) MMP-responsive hydrogels with tunable degradation rates. © The Royal Society of Chemistry 2024.^52^ (D) Degradation products of hydrogel materials are metabolized and excreted *via* the liver or kidneys.

In summary, both natural and synthetic hydrogels, when utilized as materials for micro/nano-robotic medical devices, are capable of fulfilling the requirements for most specific operations in the biomedical field. As illustrated in Fig. 4, hydrogel-based micro/nano-robotic medical device materials, under ideal conditions, achieve a sustainable cyclic pathway. This systematically delineates the closed-loop process from material design to clinical translation, encompassing the entire chain of events for hydrogel-based micro/nano-robotic medical devices, from construction, actuation, and response to degradation and excretion. This also provides an intuitive foundation and theoretical support for subsequent biosafety assessment and analysis of clinical translation potential. However, significant challenges still persist regarding precise control within complex *in vivo* environments. Future research necessitates further investigation into material innovation and multi-actuation synergistic strategies to enhance the functionality of hydrogel materials, improve the stability and controllability of hydrogel-based micro/nano-robotic medical devices, and expand their applications in clinical therapy.

#### 2.1.3. Biological safety assessment

During the critical phase of clinical translation for hydrogel-based micro/nano-robotic medical devices, systematic biological safety assessment is no less important than innovations in actuation mechanisms. To ensure the scientific rigor and completeness of this assessment, we must deeply consider multiple independent dimensions: namely, the intrinsic toxicity of the materials themselves, the toxic effects of their degradation products, and the potential risks posed by the long-term accumulation of functional nanoparticles.

Regarding the intrinsic toxicity of the materials themselves, hydrogels, as biomedical materials, typically have matrices designed for good biocompatibility. However, not all hydrogels, especially synthetic ones, can completely avoid intrinsic toxicity. While hydrogels prepared from natural polymers such as gelatin, alginate, and hyaluronic acid generally exhibit good biocompatibility, synthetic hydrogels may induce cytotoxicity or immune responses if unreacted monomers, photoinitiators, or chemical crosslinkers remain after polymerization or crosslinking. For instance, the polymerization reactions used in synthetic hydrogels, as employed by Hushka *et al.*, are rarely 100% complete. Residual unreacted active functional groups such as DBCO, azide, FPBA, and ND may slowly leach from the gel, causing chemical toxicity to surrounding cells and tissues or triggering unpredictable biological reactions.^53^ Furthermore, incompletely polymerized acrylamide-based monomers, even in trace amounts, can lead to inflammatory responses or cytotoxicity due to their reactive vinyl groups being capable of undergoing Michael addition reactions with biomacromolecules such as protein thiols or amines.^54^ Therefore, when evaluating the intrinsic toxicity of the materials themselves, it is recommended to employ rigorous *in vitro* cell viability assays, such as CCK-8, MTT, and hemolysis tests, as well as short-term animal implantation observations compliant with Good Laboratory Practice (GLP), including assessment of local inflammatory responses and tissue compatibility. This aims to clarify the direct toxic effects of the bulk material on the host before degradation. These assessments must align with the latest regulatory science literature for nanomedicines, such as the recommendations for novel biomaterial toxicity assessment found in ref. 55.

Regarding the toxicity of degradation products, the *in vivo* degradation of hydrogels is one of the critical aspects of their biological safety assessment. The chemical nature and toxicological effects of these degradation products directly determine the long-term safety of the material. Hydrogels undergo diverse degradation pathways, including hydrolysis, enzymatic degradation, and responsive degradation triggered by specific pH or redox environments. For example, the rapid stress relaxation properties of the hydrogels reported by Hushka *et al.* originate from the dynamic boronate ester bonds formed between fluorophenylboronic acid and nitrodopamine. The *in vivo* toxicity, inflammatory response, and long-term safety of these chemical groups and their degradation products, such as boronic acid, benzene derivatives, and catecholamines, remain unknown. Researchers have only demonstrated their non-toxicity to organoid cultures for a few days, but comprehensive *in vitro* cytotoxicity assays and *in vivo* acute/sub-chronic toxicity studies are lacking. Local accumulation of these products could lead to a decrease in microenvironmental pH, triggering cellular stress responses, and even affecting the activity of key biomolecules such as matrix metalloproteinases by altering enzyme conformations and the protonation states of active sites, thereby interfering with tissue repair processes.^53^ In contrast, the enzymatic or hydrolytic degradation products of natural polysaccharide hydrogels, such as chitosan and alginate, are generally considered biosafety-friendly. However, their degradation rates and product compositions still require strict control. Excessively rapid degradation or abnormal product accumulation could still adversely affect the local cellular microenvironment, necessitating a comprehensive assessment of their long-term biological safety.^56^ To precisely assess the toxicity of degradation products, it is recommended to combine advanced analytical techniques such as liquid chromatography-mass spectrometry (LC-MS) and high-performance liquid chromatography (HPLC) to identify the types and quantities of degradation products. Furthermore, based on the latest preclinical toxicology guidelines,^57^ acute/chronic animal models should be designed to independently evaluate the toxicological effects of these degradation products, avoiding confusion with the risks posed by the bulk material, in order to meet future clinical trial submission requirements.

Regarding the long-term accumulation risks of functional nanoparticles, to achieve various complex functions such as magnetic actuation, photothermal therapy, and catalysis, hydrogel-based micro/nano-robotic medical devices often require the incorporation of functional nanoscale components, such as Fe_3_O_4_, CuS, Au, and MnO_2_, into their hydrogel matrices. However, prolonged retention of these exogenous nanoparticles in the body can trigger a range of adverse reactions, including chronic inflammation, oxidative stress, and even lead to tissue fibrosis. For instance, to promote cell adhesion, Hushka *et al.* incorporated RGD peptides into hydrogels.^53^ While the RGD sequence is a universal recognition motif for integrins and can promote organoid growth, it may also non-specifically activate other cell types, such as immune cells and fibroblasts. In *in vivo* applications, this could potentially exacerbate fibrotic encapsulation or inflammatory responses.^53^ Although some functional nanoparticles can be cleared from the body through specific mechanisms, long-term or repeated administration still poses a risk of toxicity. For example, Wang *et al.* developed a magnetically actuated bionic drug-loading hydrogel-based micro/nano-robotic medical device based on ultrasmall iron oxide nanoparticles (IONPs), achieving precise drug delivery through a customized three-dimensional magnetic control platform. Despite demonstrating excellent targeting and therapeutic effects in animal models, its future application in humans still faces a series of safety challenges. While IONPs smaller than 10 nm are conducive to renal clearance, long-term or repeated administration may lead to the accumulation of iron ions in mononuclear phagocyte system organs such as the liver and spleen, inducing iron overload or oxidative stress, such as the Fenton reaction.^58^ To quantify and assess the long-term risks of functional nanoparticles, it is recommended to employ highly sensitive techniques, such as inductively coupled plasma mass spectrometry, to quantitatively analyze the residual amounts of nanoparticles in major organs like the liver, spleen, and kidneys, as well as in target tissues. Concurrently, a comprehensive assessment of potential toxicity and inflammatory responses under conditions of long-term or multiple interventions should be conducted, combining histopathological examinations (*e.g.*, H&E staining, Masson's staining) and immunohistochemical analysis of inflammatory markers (*e.g.*, IL-6, TNF-α), in accordance with the latest biosafety evaluation standards.^55^ At the material design level, priority should be given to selecting biodegradable nanoparticles that comply with regulatory requirements to mitigate the risk of long-term retention. This aligns with the principles for biosafety assessment of advanced cell therapy products outlined in ref. 59.

Key indicators and experimental conclusions from toxicological assessments of different types of hydrogels and their composite systems are also particularly important. As shown in Table 2, these allow for a direct comparison of the risks and benefits of various material systems in biomedical applications.

**Table 2:** Summary of toxicology for various hydrogels

Material category	Internal sources of toxicity	Degradation products	Nanoparticle risks	Toxicity data	Immunogenicity results	Prevention strategies	References
Natural hydrogel	Negligible (typically exhibiting good biocompatibility)	Biologically benign degradation products, such as monosaccharides	Low	Good cell viability test results, *e.g.*, HEK293 cell viability >95%	Typically no significant inflammation	No additional measures required	60 and 56
Synthetic hydrogel	Residual unreacted monomers, photoinitiators, or chemical crosslinkers	Potentially toxic small molecular substances, such as boric acid, benzene derivatives, and catecholamines	Low, unless functional nanoparticles are intentionally doped	Can cause cytotoxicity or inflammatory responses, *e.g.*, toxicity from acrylamide-based monomers	Enhanced immune response or inflammation, *e.g.*, elevated IL-6	Complete polymerization, thorough washing; rigorous *in vitro* cell viability tests and short-term animal implantation observations	54, 53 and 55
Composite hydrogel	Introduction of exogenous functional components, such as magnetic nanoparticles and RGD peptides	Hydrogel matrix fragments and degradation products of functional nanoparticles, such as iron ion accumulation	High, such as risks of long-term retention, accumulation, and oxidative stress	Residual nanoparticle levels in organs, such as liver *C*_max_, oxidative stress, and tissue fibrosis	Chronic inflammation, fibrosis, immune cell activation, *e.g.*, elevated TNF-α	Material design optimization, surface modification to evade immune recognition; selection of biodegradable nanoparticles; controlled release design	53, 58, 55 and 59

### 2.2. Lithography

#### 2.2.1. 3D printing

**Fig. 5:**
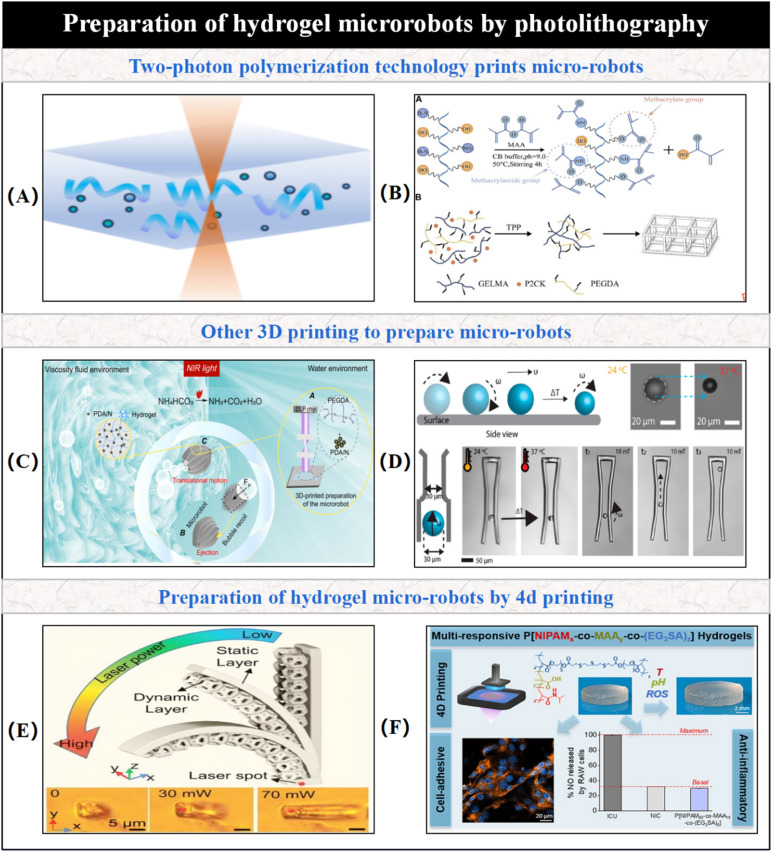
Preparation of hydrogel-based micro/nano-robotic medical devices *via* photolithography. (A) 3D printing of functional hydrogels using two-photon polymerization (TPP) technology. (B) Schematic illustrating GelMA material synthesis and the two-photon polymerization (TPP) fabrication process. © 2024 Fu and Yu.^61^ (C) Locomotion mechanism and fabrication method of photothermally driven hydrogel-based micro/nano-robotic medical devices in different fluid environments. © 2023 The Authors. Advanced Intelligent Systems published by Wiley-VCH GmbH.^68^ (D) Active regulation mechanism of temperature-responsive micro/nanostructures on magnetically actuated behavior and their application principles in microscale capture/release operations. © 2020 The Authors. Published by American Chemical Society.^69^ (E) Femtosecond laser 4D printing of photo-responsive smart hydrogel-based micro/nano-robotic medical devices. © 2023 Wiley-VCH GmbH.^70^ (F) Achieving triple environmental responsiveness in 4D printed hydrogels through molecular design innovation (EG_3_SA monomer). Copyright © 2024 The Authors. Published by American Chemical Society.^71^

Two-photon polymerization (TPP) is one of the most important 3D printing techniques for hydrogel-based micro/nano-robotic medical devices, offering ultra-high resolution down to 100 nm. As shown in Fig. 5(A), the core chemical principle behind 3D printing functional hydrogels using TPP lies in the monomers (such as GelMA and PEGDA) containing acrylate or methacrylate groups, and photoinitiator molecules. Upon absorbing two near-infrared (NIR) photons, the photoinitiator is excited to generate free radicals. These free radicals then initiate a chain-growth free-radical polymerization reaction of the monomers, forming a stable covalently crosslinked three-dimensional polymer network. Due to this nonlinear excitation mechanism, polymerization occurs only at the focal point of the NIR laser beam, thus achieving ultra-high resolution fabrication at the 100 nm scale, preventing undesired effects in other areas. Fig. 5(B) illustrates the core schematic of GelMA material synthesis and the TPP fabrication process. The synthesis mechanism of highly substituted GelMA, whose high reactivity stems from the chemical reaction of abundant amine and hydroxyl groups in the gelatin molecule with methacrylic anhydride, introduces a large number of photopolymerizable methacrylate groups onto its side chains. These carbon–carbon double bonds exhibit high reactivity under photoinitiation, forming the basis for subsequent high-resolution TPP. The synergistic effect of a photosensitive solution (GelMA + PEGDA + P2CK) and femtosecond laser enables micro-scale precision hydrogel molding.^61^ By altering the focal trajectory of the laser beam, the geometric shape of two-photon polymerized prints can be easily customized. Therefore, TPP holds immense potential for the precision manufacturing of hydrogel-based micro/nano-robotic medical devices with customized and complex structures, applicable to promising applications such as drug delivery. Billiet *et al.* noted that a major drawback when using hydrogels is their lack of mechanical strength, thus maintaining and improving the mechanical integrity of processed scaffolds has become a critical issue for 3D hydrogel structures.^62,63^ Addressing this issue, Fu and Yu first combined DS220 GelMA with PEGDA for TPP, overcoming the mechanical limitations of traditional GelMA and achieving stable fabrication of sub-micron biocompatible structures. The synergistic covalent crosslinking of high DS GelMA and PEGDA significantly enhances the mechanical properties of TPP structures, enabling self-supporting micro/nano-scale hydrogels.^64^ Lee's team, utilizing two-photon polymerization 3D micropatterning technology in conjunction with Nanoscribe equipment, achieved decoupled multifunctional operations through hierarchical material design, addressing the limitations of functional coupling and insufficient environmental adaptability in traditional hydrogel-based micro/nano-robotic medical devices. They fabricated hydrogel-based micro/nano-robotic medical devices with magnetic field, temperature, and pH responsiveness, with precision down to the nanoscale.^65^ However, TPP technology involves complex and expensive equipment, leading to significant attention on other 3D printing techniques. Sanchis-Gual *et al.* developed polyvinyl alcohol-based magnetic hydrogel micro/nano-robotic medical devices with varying morphologies and tunable stability by combining 3D-printed template-assisted casting with a salting-out process. Direct laser writing was used to prepare 3D micromolds for shaping PVA magnetic nanoparticle composite hydrogel micro/nano-robotic medical devices with high architectural complexity. These PVA-based magnetic micro/nano-robotic medical devices are suitable platforms for targeted drug and cell delivery.^66^ Multi-Material Cryoprinting (MCP) technology is a technique for fabricating hydrogel soft micro/nano-robotic medical devices and devices with complex 3D structures. Utilizing a low-temperature solvent phase transition strategy, hydrogel precursors can be rapidly solidified in environments ranging from −30 °C to −10 °C, achieving high-resolution printing with a minimum line width of 42 μm. This process also enables simultaneous chemical crosslinking reactions, making it suitable for the construction of multi-material 3D structures.^67^ Advantages of using 3D printing for fabricating hydrogel-based micro/nano-robotic medical devices include: precise control over shape and structure, ability to create complex geometries, capability to design specific internal structures, material selection flexibility, use of various biocompatible hydrogel materials, and the ability to achieve multi-material composite printing.^16,62,67^ As shown in Fig. 5(C), Chen *et al.* proposed a method combining photothermally responsive hydrogels with nanoparticles, precisely constructing PDA/N–P photothermal hydrogel-based micro/nano-robotic medical devices using DLP 3D printing technology. The actuation mechanism relies on the high near-infrared (NIR) light absorption capacity of polydopamine (PDA) nanoparticles. Under NIR light excitation, PDA non-radiatively converts light energy into heat, leading to a sharp local temperature increase in the hydrogel, which promotes a rapid phase transition (vaporization) of water within the hydrogel network to generate microscopic bubbles. The expansion and ejection of these bubbles provide thrust for the hydrogel-based micro/nano-robotic medical devices, enabling jet propulsion. The asymmetric structure and high specific surface area concentrate bubbles at the tail, propelling high-speed forward motion. In highly viscous liquids like glycerol, the hydrogel-based micro/nano-robotic medical devices achieve directional translational motion through continuous bubble recoil, overcoming viscous drag.^68^

#### 2.2.2. 4D printing

Traditional 3D printing yields static structures with limited functionality in complex *in vivo* environments. In contrast, 4D printing introduces a temporal dimension, enabling printed structures to dynamically change shape or function in response to external stimuli. By integrating smart materials capable of responding to external environmental changes—such as thermosensitive composite hydrogels achieved by grafting PNIPAM segments for LCST phase transition, or magnetically responsive ones incorporating superparamagnetic Fe_3_O_4_ nanoparticles—into micro/nanoscale structures, the resulting hydrogel-based micro/nano-robotic medical devices can dynamically react to external stimuli. Alternatively, 4D printing can be leveraged to preset micro/nanoscale structures (*e.g.*, helical, star-shaped) that undergo controlled deformation under external stimuli, thereby accomplishing tasks like targeted drug delivery and minimally invasive surgery. As shown in Fig. 5(D), Lee *et al.* first integrated 4D printing, multi-stimuli responsive materials, and magnetically actuated medical devices. This figure illustrates the active control mechanism of temperature-responsive micro/nanostructures on magnetic actuation behavior, and their application principles in microscale capture/release operations. Through 4D printing, they achieved a multi-physical field coupling mechanism, such as magnetic–thermal–mechanical cascading.^69^ For instance, Sadraei and Naghib reported the combination of magnetic nanoparticles with hydrogels to fabricate biocompatible and magnetically responsive smart materials. Hydrogel-based micro/nano-robotic medical devices constructed from such hydrogels can perform specific biomedical operations like drug delivery and can also achieve shape memory effects through magnetic field-induced heating. They noted that magnetically controlled 4D printing is still in its early stages of development, and future innovations in materials and processes hold promise for breakthrough applications in fields such as medicine and robotics.^72^ Subsequently, Young Cho *et al.* developed a 4D printing technique for biodegradable, untethered, multi-stimuli responsive soft hydrogel-based micro/nano-robotic medical devices, utilizing functional gradient porous materials (FGMM). By controlling the crosslinker concentration gradient and the physical distribution of magnetic functional particles, they formed a porous hydrogel network with humidity/magnetic responsiveness. These soft medical devices can achieve directed crawling and turning through changes in environmental humidity and external magnetic field control. The polyvinyl alcohol-based material exhibited a hydrolysis degradation rate exceeding 96% within 72 hours in phosphate buffer, demonstrating environmental friendliness. The stability of the gradient structures was verified through techniques such as SEM, EDS, and ATR-IR, and printing parameters were optimized.^73^ To adapt to more complex environments, Wang *et al.* fabricated magnetic polymers based on polylactic acid, doped with NdFeB magnetic particles, exhibiting both magnetic responsiveness and shape memory effects. Their Young's modulus could be varied within the range of 0.2–500 MPa, switching between soft/hard states *via* temperature between 25–135 °C. Magnetic shape memory polymers (MRSMPs) were programmed through self-assembly or folding guidance, combined with pulsed magnetic field magnetization, to achieve a continuous three-dimensional magnetic anisotropic distribution, overcoming the spatial limitations of traditional magnetization techniques.^74^ In Fig. 5(E), femtosecond laser 4D printing technology achieved heterogeneous integration of static/dynamic layers within a single material system. By controlling the polymer's crosslinking density through a laser power gradient, and consequently influencing the local deformation behavior of the dynamic layer, they demonstrated a low-threshold, micron-scale photo-driven mechanical work output capability.^70^ 4D printing is also widely applied in multifunctional responsive hydrogels, from material design to biomedical applications. As shown in Fig. 5(F), by using (EG_3_SA)_2_ with two acrylate functional groups as a crosslinker, thermosensitive, pH-responsive, and reactive oxygen species (ROS)-responsive units were combined within the monomer, simultaneously endowing the hydrogel-based micro/nano-robotic medical device with covalent crosslinking functionality and ROS responsiveness.^71^

3D printing technology is a commonly employed method for fabricating hydrogel-based micro/nano-robotic medical devices. It involves layer-by-layer material deposition *via* a printhead, with precise control of the printhead to accurately create desired specific structures. Firstly, 3D printing technology offers high design flexibility, enabling intricate control over the structure and morphology of these devices. This leads to greater adaptability in terms of functionality and performance. Secondly, the production process allows for personalized and customized design, manufacturing, and fabrication to meet specific requirements and application scenarios. Furthermore, 3D printing boasts high scalability and reproducibility, facilitating large-scale production of hydrogel-based micro/nano-robotic medical devices, which enhances fabrication efficiency and reduces costs. However, current 3D printing technologies employed for fabricating these devices present several drawbacks and limitations. Firstly, resolution and fabrication precision are limited, making it challenging to achieve nanoscale intricate structures. Secondly, material properties, including their flowability and viscosity during the printing process, can result in surface roughness and morphological errors in the fabricated hydrogel-based micro/nano-robotic medical devices. Moreover, 3D printing is a relatively slow process, and extended printing times can lead to material instability and variations in quality. Current techniques are primarily constrained by material selection, nanoscale control, and the integration of multifunctional materials. Further development of 3D printable nanomaterials is required to address issues related to their rheological properties and printability. Additionally, enhancing the photocuring reaction rate or crosslinking efficiency in 3D printing is crucial for fabricating finer micro- and nanostructures at the nanoscale. The integration of multifunctional materials presents a challenge, necessitating consideration of the chemical compatibility and interfacial bonding between different materials.

### 2.3. Electrodeposition technology

**Fig. 6:**
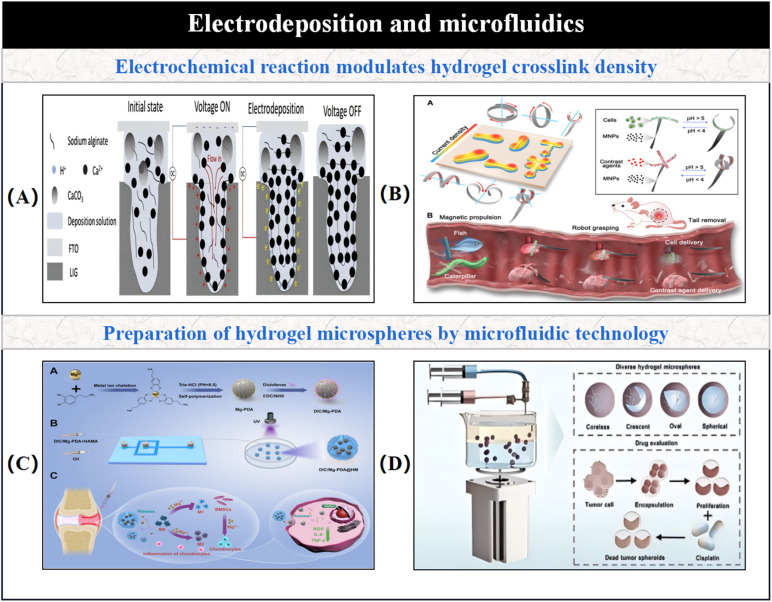
(A) Real-time regulation of hydrogel crosslinking density using electrochemical reactions, imparting gradient deformability to the structure. © 2023 The Authors. *Advanced Science* published by Wiley-VCH GmbH.^75^ (B) A modular micro/nano-robotic medical device system with a main framework composed of pH-responsive hydrogels, featuring gradient crosslinking density formed through anisotropic electrodeposition, capable of performing multi-tasks *in vivo*. Copyright © 2022 The Authors, some rights reserved; exclusive licensee American Association for the *Advancement of Science*.^76^ (C) Schematic diagram of hydrogel microsphere preparation using microfluidic technology. © 2025 Xu, Ma, Hu, Liu, Yang, Chen, Xu, Wang, Luo and Chen.^79^ (D) Rapid fabrication technology for hydrogel microspheres based on a rotating microfluidic system (RMS) and its full workflow application in tumor drug evaluation. Copyright © 2025 American Chemical Society.^71^

Electrodeposition is a technique that forms materials by driving the directional migration of ions in a solution under an electric field, followed by deposition on the electrode surface. In recent years, this technique has been widely applied to the controlled synthesis of hydrogel-based micro/nano-robotic medical devices. This technique enables precise molding of microscale structures by controlling electric field parameters such as voltage, current, and electrode shape. It utilizes H^+^ or OH^−^ generated from water electrolysis to alter local pH, which in turn induces ionization or deionization of pH-sensitive polymers, such as the ionic crosslinking of sodium alginate's carboxyl groups with Ca^2+^. As shown in Fig. 6(A), electrochemical reactions are employed to real-time regulate the local pH value inside the hydrogel, which then influences the charge state of ionizable groups in the hydrogel network. This enables controllable adjustment of crosslinking density, endowing the structure with gradient deformation capability. Functional components such as magnetic particles and biomolecules can be integrated in a single-step process, playing a crucial role in achieving multi-actuation cooperation for hydrogel-based micro/nano-robotic medical devices. For instance, Zheng *et al.* proposed a one-step anisotropic electrodeposition method for fabricating modular hydrogel-based micro/nano-robotic medical devices with different functionalities in each modular part.^75^ By programming the electric field, micro-striped structures can be endowed with various shape deformation capabilities, such as helix, twist, bend, and coil. Photolithography is used to pre-pattern molds with the aid of masks, and then hydrogel-based micro/nano-robotic medical devices are prepared modularly *via* electrodeposition. This provides a safe, flexible, intelligent, and multifunctional integrated design paradigm for next-generation hydrogel-based micro/nano-robotic medical devices, particularly suitable for gastrointestinal drug delivery, tissue engineering, and other scenarios, addressing the issues of single functionality and insufficient biocompatibility in traditional micro/nano-robotic medical devices.^76^ To address the issue of single functionality and locomotion modes, Zhong *et al.* employed an electrodeposition process to fabricate ultra-soft magnetic hydrogel-based micro/nano-robotic medical devices. Photolithographic patterns were created on a conductive glass substrate, followed by dropping a solution containing sodium alginate, calcium carbonate, and magnetic particles. A constant current was applied to initiate water electrolysis, generating H^+^ ions in the cathodic region. These H^+^ ions reacted with calcium carbonate (CaCO_3_) to produce Ca^2+^ ions. These Ca^2+^ ions then immediately reacted with the carboxylate groups on the sodium alginate polymer chains, triggering the gelation of sodium alginate and forming deformable, ultra-soft, magnetically controlled hydrogel-based micro/nano-robotic medical devices. This method allows for optimizing magnetic response sensitivity and locomotion efficiency by adjusting the concentration and distribution of doped magnetic particles, while also enabling mass production and adapting to complex biological environments through morphological switching.^77^ Regarding the functionalization of hydrogels *via* electrodeposition technology, as depicted in Fig. 6(B), the hydrogel-based micro/nano-robotic medical device proposed by Zheng *et al.* features a main skeleton composed of pH-responsive hydrogel in its core functional area. By precisely controlling the electric field distribution, a local pH gradient is induced, which in turn regulates the ionization degree of polymer chains and the crosslinking reaction rate, forming an anisotropic structure with a gradient distribution of crosslinking density, thereby achieving fluorescence imaging.^76^ Mold casting is a common method for preparing hydrogels. Molds are designed and prepared, then treated according to the different reaction conditions for hydrogel formation, such as physical crosslinking, chemical crosslinking, and photopolymerization, until the hydrogel is crosslinked. This is a relatively simple and cost-effective method, facilitating the preparation of various shapes. Simultaneously, mold casting can be used modularly for preparing hydrogel-based micro/nano-robotic medical devices. Functional materials can be directionally deposited within hydrogels through electrochemical processes, suitable for constructing magneto-responsive structures. Zhu *et al.* proposed a method for efficient preparation of high-precision microstructures on complex curved surfaces using photo-responsive hydrogels, addressing the problem of traditional techniques relying on expensive equipment and complex procedures. By using hydrogels as flexible micromolds, microstructures on the hydrogel surface are processed *via* UV photolithography, then transferred to materials like polydimethylsiloxane (PDMS), finally casting complex curved structures with nanoscale surface smoothness.^78^ Mold casting and electrodeposition techniques complement each other in the fabrication of hydrogel-based micro/nano-robotic medical devices: mold casting excels at efficient replication of macroscopic structures, while electrodeposition is adept at *in situ* integration of nanoscale functionalities. In the future, through the integration of processes and innovation in material chemistry, it is expected to overcome single-technology bottlenecks and advance hydrogel-based micro/nano-robotic medical devices towards higher intelligence and functionalization.

### 2.4. Microfluidics

**Fig. 7:**
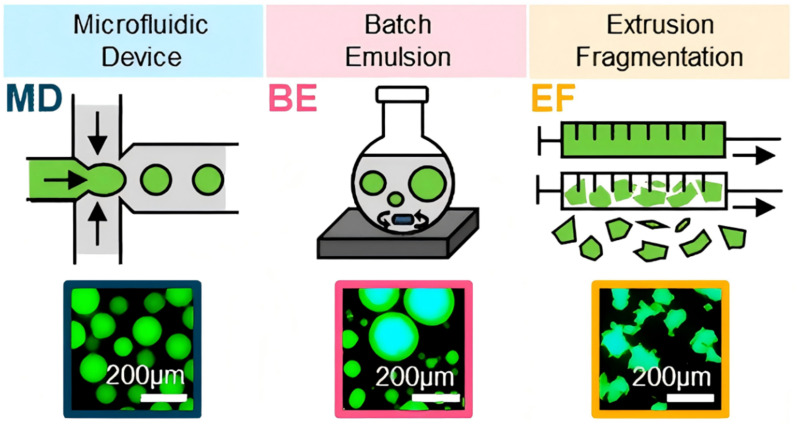
Working principles, product morphology differences, and the mechanism by which they influence the properties of particulate hydrogels for three microgel preparation techniques. Copyright© 2021 American Chemical Society.^82^

Microfluidic technology, which enables precise control of fluid flow at micro-scales, is widely applied in the preparation of hydrogel micro/nanoparticles and the design of hydrogel-based micro/nano-robotic medical devices. As shown in Fig. 6(C), microfluidic technology is used for preparing hydrogel microspheres. By adjusting parameters such as shear force and pressure gradient, microfluidic systems can generate highly uniform hydrogel microparticles and achieve densification through crosslinking reactions. Its core functions are manifested in precise structural control, integration of functional modules, efficient production processes, and assurance of biocompatibility.^79^ Microfluidic technology allows for precise control of droplet generation and crosslinking, making it suitable for producing multifunctional hydrogel microspheres of identical size. For example, Zhao *et al.* prepared stimulus-responsive hydrogel microparticles with rapid response rates and high drug loading capacities using microfluidic technology. The microparticle size ranges from 50 nm to 200 μm, making them suitable for various complex environments. They also noted that the control precision of hydrogel microspheres prepared by this technique needs improvement regarding multi-stimuli synergistic response.^80^ Subsequently, Wang *et al.* developed an injectable hydrogel system with reactive oxygen species (ROS)-responsive properties for dynamically regulating the cellular microenvironment, thereby enhancing the precision and stability of hydrogel microspheres prepared by this technique under dynamic regulation.^81^ The achievements of microfluidic technology in preparation include multifunctional hydrogel microspheres. These hydrogels can achieve drug delivery or deformation in response to specific stimuli, acting as composite hydrogels. Composite hydrogels are, in turn, important materials for realizing the multifunctionality of hydrogel-based micro/nano-robotic medical devices. These hydrogel microspheres serve not only as crucial constituent materials for hydrogel-based micro/nano-robotic medical devices but also find widespread applications in disease treatment. As shown in Fig. 6(D), Cheng *et al.* achieved high-speed, low-cost, and morphologically controllable preparation of hydrogel microspheres using a Rotating Microfluidic System (RMS), and validated the significant impact of microsphere morphology on drug efficacy using tumor spheroid models, providing a theoretical basis and experimental platform for targeted delivery system design.^24^ Therefore, microfluidic technology is one of the important techniques for preparing hydrogel-based micro/nano-robotic medical devices, but it cannot fully fabricate them on its own. Similar to microfluidic devices are batch emulsion and extrusion-breakup methods. As shown in Fig. 7, Muir *et al.* described the process principles and product morphology differences of three microgel fabrication techniques (MD/BE/EF), and how the morphological characteristics of microgels ultimately determine the mechanical properties, pore structure, and injectability of particulate hydrogels.^82^

### 2.5. Other

**Fig. 8:**
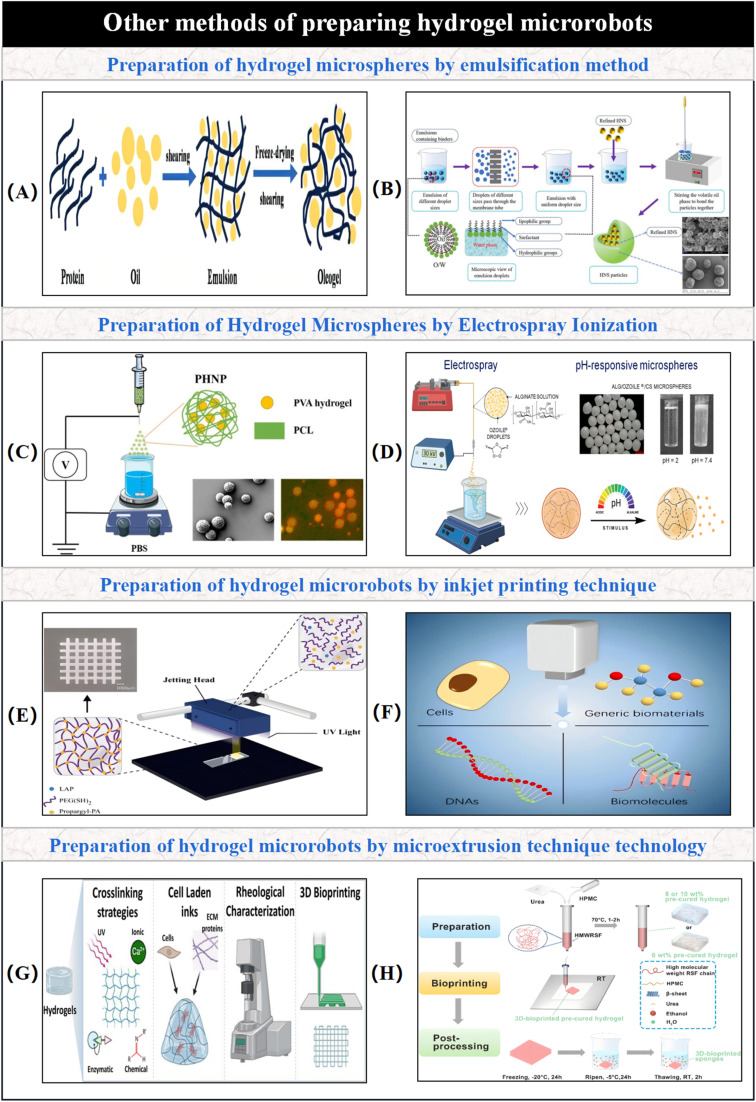
(A) Preparation of hydrogel microspheres *via* emulsification method. © 2025 The Authors. Published by Elsevier Ltd.^87^ (B) Core process and interfacial chemical mechanism for preparing hexanitrostilbene microspheres *via* membrane emulsification technology. © 2022 Chinese Society of Particuology and Institute of Process Engineering, Chinese Academy of Sciences.^88^ (C) Double-walled hydrogel prepared by single-needle electrospray method. © 2019 Elsevier B.V. All rights reserved.^89^ (D) Full process, material design principle, and functional verification mechanism for preparing pH-responsive alginate/ozonated oil microspheres using electrospray technology. © 2024 by the authors. Licensee MDPI, Basel, Switzerland.^91^ (E) Inkjet printing for fabricating hydrogel-based micro/nano-robotic medical devices. © 2024 The Authors. Published by American Chemical Society.^92^ (F) Inkjet bioprinting technology as a multifunctional biomanufacturing platform for integrated processing of four key biological components to fabricate functional hydrogels. © 2020 American Chemical Society.^93^ (G) Microextrusion technology for fabricating hydrogel-based micro/nano-robotic medical devices. © 2025 Elsevier Ltd. All rights are reserved, including those for text and data mining, AI training, and similar technologies.^94^ (H) Low-temperature deposition 3D bioprinting and gradient cryoforming technology for pre-crosslinked silk fibroin hydrogels. © The Royal Society of Chemistry 2022.^95^

In addition to the commonly used fabrication techniques mentioned above, emulsification and electrospray ionization methods are employed for preparing hydrogel microspheres, while inkjet printing and microextrusion technologies are utilized for fabricating hydrogel-based micro/nano-robotic medical devices.^83–86^ As shown in Fig. 8(A and B), hydrogel microspheres can be prepared *via* membrane emulsification. Emulsification involves mixing immiscible liquids. With the aid of surfactants, mechanical stirring or microfluidic shear forces reduce the oil–water interfacial tension, generating crosslinkable hydrogel droplets. Surfactants form an adsorbed layer at the interface, directionally stabilizing HNS particles. Subsequent volatile solidification forms highly dense, low-defect HNS microspheres. This process overcomes the particle size dispersion and density bottlenecks of traditional crystallization methods, significantly enhancing the safety and energy output efficiency of energetic materials.^87,88^ The electrospray ionization method is also one of the commonly used techniques for preparing hydrogel microspheres. Electrospray ionization is a potential-driven liquid atomization technique where a high-voltage electric field is applied to charge a polymer solution, forming charged droplets. These droplets interact with an ionic crosslinker during flight, undergoing ion exchange and coordination crosslinking reactions, thereby promoting the synthesis of hydrogel microspheres. As depicted in Fig. 8(C), a double-walled hydrogel prepared by a single-needle electrospray method consists of an outer layer of polyvinyl alcohol hydrogel with hydrophilicity and adjustable swelling properties, and an inner layer of polycaprolactone, which provides hydrophobicity, mechanical support, and slows drug release.^89^ While both electrospray ionization and microfluidic technologies are used for preparing hydrogel microspheres, Shao *et al.* noted that emulsification and electrospray ionization methods often suffer from certain limitations, leading to non-uniform particle sizes in the prepared hydrogel microspheres.^90^ In Fig. 8(D), pH-responsive alginate/ozonated oil microspheres were prepared using the electrospray technique. The carboxyl groups in alginate become protonated in acidic environments, leading to reduced polymer chain hydrophilicity and weakened electrostatic repulsion, causing the microspheres to contract and protect the drug. In neutral environments, the carboxyl groups deprotonate, increasing electrostatic repulsion and hydration, which results in hydrogel swelling and precise drug release. This offers a new strategy for targeted delivery in inflammatory bowel disease.^91^ Inkjet printing is widely used for stimulus-responsive hydrogels. As shown in Fig. 8(E and F), inkjet printing for fabricating hydrogel-based micro/nano-robotic medical devices works by obtaining digital data of a design model from a computer and forming preset objects on a substrate *via* ink droplets. Inkjet bioprinting technology, as a versatile biomanufacturing platform, can integrate four key biological components: cells, biomaterials, DNA, and biomolecules. Through precise deposition of picoliter droplets, these four elements are combined into a single manufacturing platform, enabling full-chain biomanufacturing from tissue regeneration to gene therapy, and driving the paradigm shift in life sciences from “single-functional devices” to “integrated living systems”.^92,93^ Microextrusion technology is employed for fabricating hydrogel-based micro/nano-robotic medical devices. As shown in Fig. 8(G and H), microextrusion involves loading biomaterials into a barrel and extruding them through a nozzle onto a manufacturing platform or a predetermined location on the platform using pneumatic or mechanical force. A computer-controlled movable print nozzle then deposits the final structure layer by layer. Through urea/HPMC-assisted pre-crosslinking, forming hydrogen bonds or physical entanglement, stable extrusion printing of high-concentration silk fibroin is achieved. Combined with a gradient freezing-ethanol solidification technique, biomimetic porous scaffolds are constructed, addressing the concentration limitations and uncontrolled structures of traditional silk fibroin bioprinting, and providing a novel customized material platform for cartilage repair.^94,95^

In summary, micro/nano-robotic medical devices composed of composite hydrogels hold significant research value and application prospects in terms of mechanical properties and the generation of intelligent materials.^96^ By rationally designing the chemical composition and network structure of hydrogels, it is possible to fabricate micro/nano-robotic medical devices with excellent mechanical properties and chemical responsiveness. However, different fabrication techniques possess their own advantages and disadvantages, as summarized in Table 3. A single fabrication technique struggles to meet the demands for structural complexity, functional integration, and dynamic responsiveness; performance breakthroughs require the synergistic combination of multiple techniques. Therefore, the future necessitates the integration of various fabrication technologies, and establishing multi-technique synergistic platforms is the correct direction for hydrogel-based micro/nano-robotic medical devices.

**Table 3:** Advantages as well as disadvantages of different preparation methods

Methodologies	Dominance	Malpractice	Reference
Photolithography (3/4D printing)	Specific size and morphology of microrobots can be obtained, allowing direct preparation of microspheres containing cells and reducing cell damage	Relatively low production and expensive TPP technology	62 and 72
Electrodeposition technology	Efficient, programmable structural design, multifunctional integration and biocompatibility	Complex and costly equipment, uneven density of localized gel networks	76–78
Microfluidics	Highly uniform size and shape of microspheres; versatility; precise control of reaction conditions and regulation of hydrogel physicochemical properties	High maintenance cost of equipment; complexity of work; high technical requirements for personnel; low efficiency of large-scale production	80 and 81
Emulsification	Precise control of microsphere size and structure; easy to use; low cost; high yield; controllable	Uneven dispersion of emulsion; wide particle size distribution of microspheres; complicated operation procedure, precise control of emulsification conditions is required	83
Electrospray ionization	Precise control of microsphere size; flexible material selection and ability to prepare microspheres with complex structures	Limited production capacity; high equipment requirements; sensitivity to the operating environment	84
Inkjet printing	The diameter, shape and internal structure of the microrobots can be precisely controlled; different materials can be mixed to build multifunctional microspheres	Complex device parameters make it difficult to maintain biomolecular activity	85
Micro-extrusion technology			86

## 3. Actuation mechanisms and stimulus-responsive mechanisms of hydrogel-based micro/nano-robotic medical devices

**Fig. 9:**
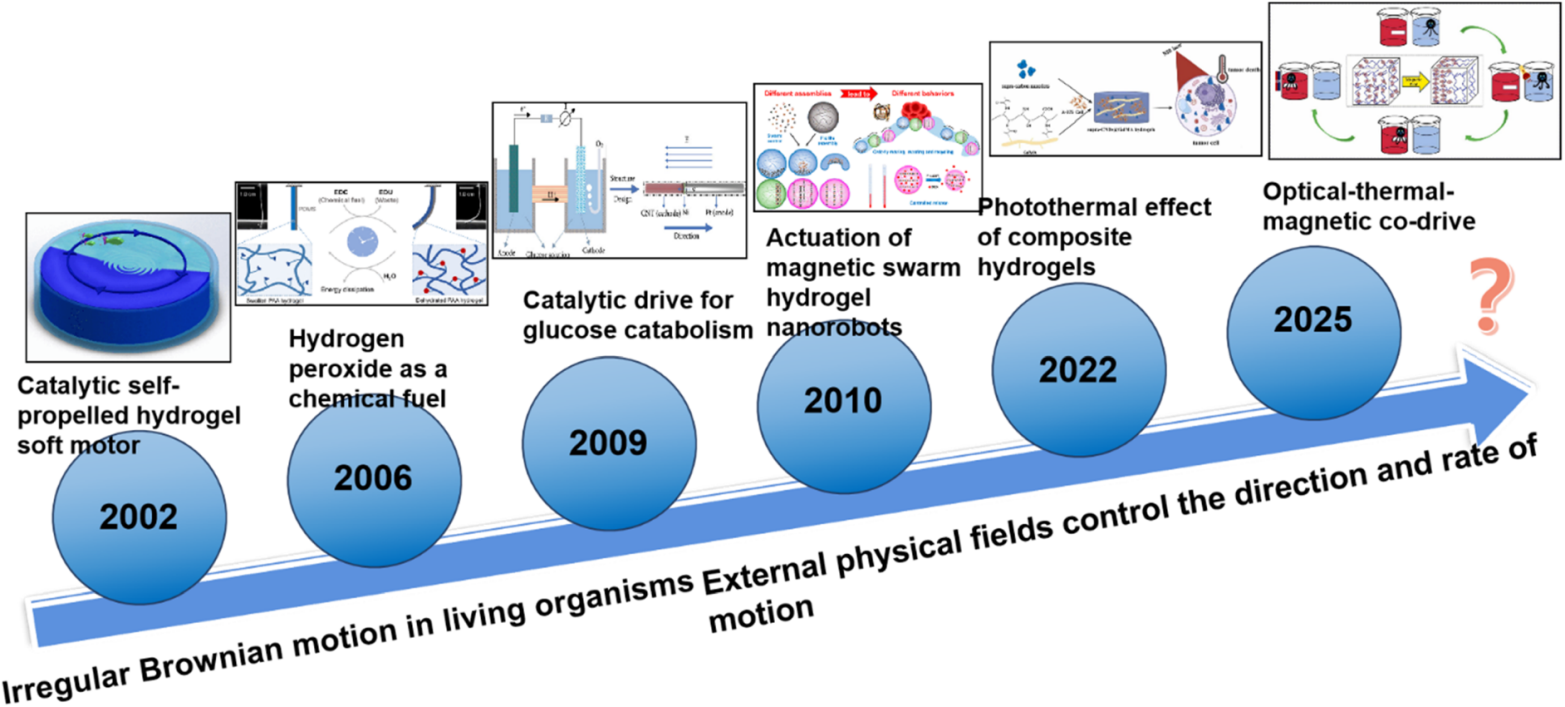
Illustrates low-energy catalytic self-propelled hydrogel soft motors © 2016 American Chemical Society;^97^ self-resetting hydrogel actuators powered by chemical fuels © 2022 American Chemical Society;^98^ hydrogel-based micro/nano-robotic medical devices utilizing glucose in human body fluids as a driving fuel © 2021 Hao Wang *et al.*;^99^ spatiotemporally driven hydrogels for magnetic swarm micro/nano-robotic medical devices © 2022 American Chemical Society;^100^ the photothermal effect of composite hydrogels under near-infrared stimulation © 2023 Elsevier B.V. All rights reserved;^101^ and achieving movement by creating asymmetric structures during fabrication to alter local field symmetry © 2023 Elsevier B.V. All rights reserved.^102^

Currently, the actuation of hydrogel-based micro/nano-robotic medical devices primarily relies on two main mechanisms: chemical fuel-driven and external field-driven actuation. The early development of hydrogel-based micro/nano-robotic medical devices centered on self-propulsion, achieving autonomous movement by responding to environmental changes through the material's inherent chemical or physical properties. Examples include low-energy catalytic self-propelled hydrogel soft motors and self-resetting hydrogel actuators powered by chemical fuels. Subsequently, the diversity of chemical fuels for self-propulsion increased, leading to hydrogel-based micro/nano-robotic medical devices that utilize glucose from body fluids as a driving fuel and those carrying hydrogen peroxide for propulsion. Chemical fuel-driven actuation of hydrogel-based micro/nano-robotic medical devices achieves irregular Brownian motion within the biological body by producing local pH or temperature changes through chemical reactions, or directly generating gas to form microbubbles. However, this method typically involves a relatively slow process to reach the target location. To enhance motion controllability and speed, research has shifted towards active regulation using external physical fields such as magnetic fields, light, and ultrasound, forming a high-precision control system for speed and direction. Examples include spatiotemporally driven hydrogels for magnetic swarm hydrogel-based micro/nano-robotic medical devices and composite hydrogels exhibiting photothermal effects under near-infrared stimulation, or achieving movement by creating asymmetric structures during fabrication to alter local field symmetry. As shown in Fig. 9, hydrogel-based micro/nano-robotic medical devices have evolved from self-propulsion mechanisms involving irregular Brownian motion of hydrogel microspheres in liquid environments to regulated movement where external physical fields combined with self-propulsion mechanisms control their direction and speed. In complex and confined biological environments, these actuation methods may be susceptible to environmental interference, leading to reduced control precision. To overcome the limitations of single actuation mechanisms, researchers have recently begun exploring hydrogel-based micro/nano-robotic medical devices that integrate multiple actuation mechanisms. Multi-actuation systems can synergistically utilize external physical fields and hydrogel chemical fuel-driven actuation. Here, direction and speed play critical roles in determining performance, with speed regulation primarily achieved through chemical reactions and movement direction controlled by external fields. Furthermore, by integrating the hydrogel's stimulus-responsive mechanisms, the precision and functionality of the device's control can be effectively enhanced, enabling more complex tasks.

Composite hydrogels play a crucial role in enabling diverse actuation possibilities for hydrogel-based micro/nano-robotic medical devices. By combining natural and synthetic hydrogels, or different types of hydrogels with other functional materials, enhanced physical, chemical, and biological properties can be achieved. In the design of hydrogel-based micro/nano-robotic medical devices, composite hydrogels, as an emerging material system, effectively address the limitations of single hydrogel materials in terms of mechanical properties, biocompatibility, and controllability.^103^ The chemical modifiability of hydrogels, achieved through the introduction of specific chemical functional groups, grafting of responsive polymer segments, or doping with functional nanomaterials, directly influences their responsive characteristics, and consequently dictates the choice of actuation method. Chemical modification can endow hydrogels with responsiveness to stimuli such as temperature, pH, magnetic fields, and light. For instance, incorporating thermosensitive polymer chains enables temperature responsiveness, aligning with temperature-driven actuation mechanisms.^8,39^ Modifying with magnetic nanoparticles confers magnetic responsiveness, providing the foundation for magnetic actuation.^19,22,48,49,60,104–119^ Adding photosensitive groups like azobenzene, which utilize photoisomerization reactions, can achieve photo-actuation.^9,16,120,121^ This association between chemical modification, responsive properties, and actuation methods is a critical aspect of designing hydrogel-based micro/nano-robotic medical devices, directly impacting their functional realization and application scenarios. For example, in fields like drug delivery and tissue engineering, the hydrogel's responsive characteristics must be customized through chemical modification to match the appropriate actuation method, based on practical needs.

### 3.1. Chemical fuel drive

Chemical propulsion mechanisms in hydrogel-based micro/nano-robotic medical devices primarily utilize chemical reactions with specific chemical fuels to generate microbubble propulsion for the purpose of movement within biological systems. Common fuels include hydrogen peroxide, glucose, ionic environments, and others. As shown in Fig. 9, most hydrogel-based micro/nano-robotic medical devices primarily composed of hydrogel materials achieve movement through chemical reactions that generate local pH or temperature changes, or directly produce gas, combined with repeated, self-resetting actions. For instance, Xu *et al.* proposed a chemical fuel-driven biomimetic self-resetting hydrogel actuator, where local pH or osmotic pressure changes generated by catalytic reactions transiently alter the protonation state of ionizable groups or the hydrophilicity/hydrophobicity of polymer chains within the hydrogel network, thereby enabling autonomous actuation and deformation, and spontaneously returning to their initial state after fuel depletion. This exemplifies this principle.^98^ Subsequently, Nan *et al.* introduced a biomimetic self-resetting bilayer hydrogel actuator. This research established a negative feedback loop between H^+^ ions produced by acid-catalyzed reactions and specific chemical components within the hydrogel, precisely controlling the deformation rate and self-resetting process of the hydrogel. Coupled with a biomimetic bilayer structural design, they developed a hydrogel actuator with self-resetting capabilities. This resolved the limitations of traditional actuating materials regarding signal irreversibility or irreversible reactions, offering an efficient and sustainable actuation solution for next-generation soft robotics.^122^ Concurrently, Zhao *et al.* combined two hydrogel layers with different functionalities through an asymmetric design, forming a bendable responsive bilayer structure. They leveraged osmotic pressure differences between the inside and outside of the hydrogel network caused by ion concentration gradients, as well as coordination or ion exchange reactions between specific ions and charged functional groups on the polymer chains, to trigger hydrogel swelling/contraction, achieving controllable deformation. This provided a bio-friendly and highly controllable actuation solution for soft robotics, with significant application potential in fields such as healthcare and environmental monitoring.^123^ Chemically driven hydrogel-based micro/nano-robotic medical devices offer advantages such as ease of application and a broad range of propulsion reactions. However, these chemical propulsion systems typically lack effective control over the locomotion characteristics of hydrogel-based micro/nano-robotic medical devices, thus necessitating the use of external magnetic fields for precise motion regulation. Furthermore, the lifespan of chemically propelled hydrogel-based micro/nano-robotic medical devices is generally limited.

### 3.2. External physical field drives

#### 3.2.1. Magnetic field drive

**Fig. 10:**
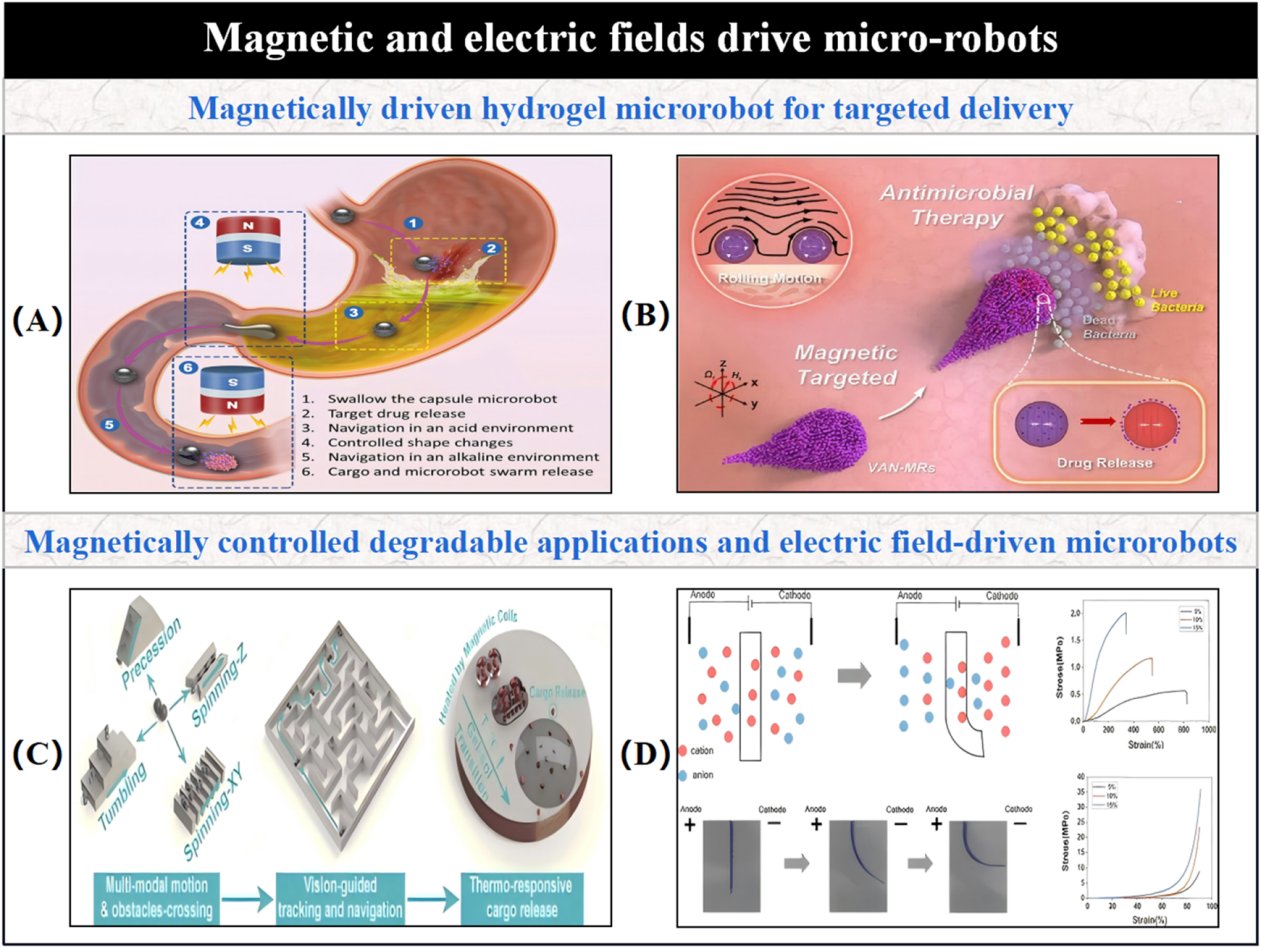
(A) Full closed-loop targeted delivery process of magnetic-controlled capsule micro/nano-robotic medical devices within the gastrointestinal tract. © 2023 The Authors.^60^ (B) Closed-loop antibacterial therapy *via* magnetic-driven VAN-MRS hydrogel-based micro/nano-robotic medical devices: targeted rolling to the infection site, local sustained release of vancomycin using a pH-responsive mechanism, achieving “precise navigation, controlled drug release, and efficient bactericidal action.” © 2025 Wiley-VCH GmbH.^107^ (C) A degradable magnetic-driven hydrogel-based micro/nano-robotic medical device with multimodal locomotion, autonomous navigation, and thermo-responsive release capabilities. © 2023 American Chemical Society.^112^ (D) Electric field-triggered directional migration of cations and anions and formation of a charge accumulation layer at the membrane interface, inducing controllable bending deformation of the material. © 2020 American Chemical Society.^124^

Magnetic fields are utilized to manipulate magnetic particles, such as Fe_3_O_4_ nanoparticles, within hydrogel-based micro/nano-robotic medical devices, enabling precise navigation through gradient or oscillating magnetic fields. Magnetic hydrogels can achieve cooperative movement of multiple devices *via* programmed magnetic fields; during intravascular navigation, swarms of these magnetic devices can be tracked in real-time using laser scattering imaging. This actuation method offers advantages such as non-invasiveness, remote control, and precise manipulation, making it suitable for *in vivo* applications and in complex environments. As shown in Fig. 1, the incorporation of superparamagnetic particles like Fe_3_O_4_ into hydrogels enables external magnetic field manipulation of hydrogel-based micro/nano-robotic medical devices. For instance, Chen *et al.* designed and fabricated a biodegradable magnetic hydrogel-based micro/nano-robotic medical device based on gelatin and ferric oxide nanoparticles, demonstrating its advantages in diverse actuation methods, flexible maneuverability, and precise motion control, making these devices attractive for biomedical applications.^112^ Magnetic field-driven hydrogel-based micro/nano-robotic medical devices have demonstrated potential applications in cancer therapy. Jiang's team utilized magnetic field-driven hydrogel-based micro/nano-robotic medical devices for loading lycorine hydrochloride to inhibit colorectal cancer cells, offering a new strategy for tumor therapy.^7^ Tao's team designed and fabricated Ro-3306-loaded magnetic field-driven hydrogel-based micro/nano-robotic medical devices to enhance the chemotherapeutic efficacy against MYC-dependent osteosarcoma, providing a new strategy for osteosarcoma treatment.^18^ By designing devices with specific shapes and structures, the gradient distribution of magnetic fields enables efficient shuttling and targeted therapy within tumor tissues. These devices can precisely deliver chemotherapeutic drugs to tumor cells and, concurrently, enhance the efficacy of tumor treatment through photothermal effects or chemotherapy synergism. The vast majority of teams apply magnetic field-driven hydrogel-based micro/nano-robotic medical devices in drug delivery, where magnetic field-driven hydrogel actuators can rotate and propel under the influence of a magnetic field. In Fig. 10(A), Xu *et al.* developed a magnetic hydrogel soft capsule micro/nano-robotic medical device, approximately 2 mm in diameter, based on a magnetic-responsive dual control mechanism, achieving precise operation in an *in vitro* human phantom through ultrasound guidance. This system employs gradient magnetic field actuation, achieving non-damaging navigation at speeds up to 1.5 mm s^−1^ through narrow channels in weak magnetic fields (<3*T* m^−1^). In strong magnetic fields (>6*T* m^−1^), it triggers physical disintegration to release cargo. It maintains structural stability in extreme pH environments, lasting over 2 hours at pH 1.5 and over 2 days at pH 12. In drug loading experiments, a Ca(OH)_2_ solution mimicked drug release, showing accelerated diffusion by a swarm of these devices, with a 300% speed increase. Biocompatibility validation showed 90% survival rate for zebrafish embryos encapsulated for 24 hours, and HEK293 cell viability >95% after co-culturing with the material. Regarding pharmacokinetic (PK), pharmacodynamic (PD), and immunogenicity, magnetic triggering enabled instantaneous drug release, increasing diffusion area by 70% within 5 seconds. The PD potential was demonstrated by lesion targeting and local enrichment of phenolphthalein gel, despite the material's good biosafety. The study systematically describes the closed-loop targeted delivery process of magnetic-controlled capsule hydrogel-based micro/nano-robotic medical devices within the gastrointestinal tract, involving oral ingestion of the capsule, followed by navigation in gastric acid environment, magnetic-controlled morphological changes, navigation in intestinal alkaline environment, swarm regrouping, and targeted release. This research was validated in various experimental scenarios simulating the human internal environment, such as constructing gastric acid and intestinal alkaline environments by adjusting pH, and creating 3D rough surfaces mimicking intestinal folds. The experiments comprehensively covered complex internal human environments, verifying the feasibility of targeted delivery of the capsule within the digestive system. Overall, through a magnetic-controlled staged strategy and environment-responsive design, precise delivery and cooperative swarm operation of capsule micro/nano-robotic medical devices in the gastrointestinal tract were achieved.^60^ The combination of hydrogels and magnetic particles enables hydrogels to respond and actuate in a tunable and wireless manner under magnetic field guidance, which is a significant advantage for further research and development of magneto-responsive hydrogels. This combination holds great application potential in biomedical fields, and the preparation methods are applicable to other filler-containing soft functional materials.^19,22,48,49,60,104–119^ For example, in Fig. 10(B), Chen *et al.* developed a magnetic-responsive hydrogel-based micro/nano-robotic medical device system, consisting of composite microspheres with magnetic Fe_3_O_4_ nanoball chains as the driving core and externally encapsulated by a poly(acrylic acid-*co*-acrylamide) hydrogel shell, for targeted antibiotic delivery. This system exhibited excellent performance in an *in vitro* human phantom, where agar gel simulated an infected site within a microfluidic channel. Through a magnetic-driven response mechanism, individual hydrogel-based micro/nano-robotic medical devices achieved rolling motion under a rotating magnetic field, reaching a peak speed of 4.8 μm s^−1^ with a stepping frequency of 6 Hz. Swarm motion achieved a peak speed of 62 μm s^−1^ with a stepping frequency of 4 Hz, successfully navigating to the target area and covering bacterial colonies. For drug loading, the hydrogel matrix achieved efficient loading of vancomycin *via* electrostatic adsorption, with a drug loading efficiency of 38.2% and sustained release characteristics, releasing 10% in 12 hours. The reservoir effect of the hydrogel pores demonstrated its local sustained release capability. Validation showed a significant inhibition of *Staphylococcus aureus* growth at a concentration of 1 mg mL^−1^, reducing bacterial survival rate to 10%. Immunogenicity assessment *via* RAW264.7 cytotoxicity testing indicated good biocompatibility, with cell viability exceeding 80%. This magnetic field-driven system offers an efficient and biosafety-conscious solution for targeted antibacterial therapy.^107^ The post-mission handling of magnetic field-driven hydrogel-based micro/nano-robotic medical devices after completing their tasks is also a crucial topic. Chen *et al.* proposed a solution to this problem, describing a degradable magnetic field-driven hydrogel-based micro/nano-robotic medical device possessing multimodal locomotion, autonomous navigation, and thermo-responsive release capabilities. Its main structure, a gelatin hydrogel, undergoes slow solation at 35–37 degrees Celsius, thereby achieving degradation. Chen *et al.* developed a biodegradable magnetic hydrogel-based micro/nano-robotic medical device that achieved multimodal locomotion and targeted drug delivery through magnetic field-driven responses in an *in vitro* human phantom simulating the intestinal surface of large animals. This device, comprising gelatin and Fe_3_O_4_ nanoparticles, forms a peanut-shaped structure with a long axis of 1.85 mm and a short axis of 0.95 mm. Driven by a rotating magnetic field of 10 mT strength and 1–9 Hz frequency, it can switch between four locomotion modes: precession, tumbling, *XY*-rotation, and *Z*-rotation, reaching a maximum speed of 12.75 mm s^−1^. It demonstrated exceptional obstacle-crossing capabilities, including climbing a 45° incline, traversing a 1.5 mm step, and navigating a 1.5 mm narrow channel. Regarding drug loading, the magnetic hydrogel-based micro/nano-robotic medical device achieved functional delivery by loading doxorubicin-chitosan microparticles, exhibiting a drug loading capacity of 5.44% ± 0.63% and an encapsulation efficiency of 51.8% ± 6.35%, with a cumulative release rate of 43.63% ± 5.33% over 12 hours. Similar to previous examples, the porous structure of the hydrogel demonstrated its local sustained release capability. Validation showed an inhibitory activity against HCT116 colon cancer cells of 56.8% ± 3.11%, compared to 27.54% ± 4.65% for the free DOX group. Immunogenicity assessment *via* 293T cytotoxicity testing showed extremely high biocompatibility, with cell viability close to 100%. This magnetic field-driven system, integrating degradability, multimodal locomotion, and intelligent drug release, provides an innovative solution for targeted delivery.^112^ Degradability was achieved through the gelatin-based hydrogel; magnetic actuation by Fe_3_O_4_ nanoball chains; and enriched functionality *via* chitosan drug-loaded microparticles. This example fully illustrates the compatibility of hydrogels as the main component of hydrogel-based micro/nano-robotic medical devices.

#### 3.2.2. Electric field drive

Actuation *via* electric fields is achieved by integrating materials with differing polarization rates, similar to mechanisms used in magnetic actuation,^112^ primarily utilizing direct current (DC) or alternating current (AC) electric fields for propulsion. Electro-responsive hydrogels are widely applied in fields such as soft robotics and microfluidic valves, but traditional hydrogels often suffer from low mechanical strength and slow response speeds. Jiang and Tang developed a high-strength and tough nanocomposite hydrogel through zirconium hydroxide nanoparticle crosslinking, exhibiting a tensile strength of up to 2 MPa and a Young's modulus of 1.2 MPa, while also possessing rapid electro-responsiveness. Its high mechanical strength ensures stability during repeated deformation, and its rapid response and controllability make it suitable for enhancing the accuracy and stability of *in vivo* operations. However, this actuation method is difficult to synergize with other actuation mechanisms, still presenting issues of singular actuation mode and functionality. As shown in Fig. 10(D), Jiang *et al.* developed a nanocomposite hydrogel based on zirconium hydroxide nanoparticle crosslinking, which achieves rapid bending actuation *via* electric field driving, effectively enhancing the mechanical strength of electro-responsive hydrogels. Electric field actuation experiments were conducted in a customized setup where a hydrogel strip was placed in a 0.5 M Na_2_SO_4_ solution, with two parallel platinum electrodes spaced 50 mm apart, and a ±15 V voltage applied. The bending mechanism originates from osmotic pressure differences caused by ion migration, leading to expansion on the anodic side and inducing anisotropic deformation. Specific experimental data show a rapid and reversible response, with a bending angle of up to 80° towards the cathode within 100 seconds under a 15 V applied voltage, and bending to −70° within 100 seconds after voltage reversal. The response rate and maximum bending angle can be adjusted by varying NP concentration, with the sample reaching 90° in 80 seconds and the highest rate observed at 10 wt%. The electric field triggers directional migration of cations and anions, forming a charge accumulation layer at the diaphragm interface, which induces controllable bending deformation of the material. Its significant softening and enhanced ductility in the energized state bestow the material with dynamically adjustable properties under high loads.^124^ Subsequently, Lee *et al.* systematically investigated the response behavior of agarose/poly(acrylic acid) double-network hydrogels in ionic environments. The core experimental system involved preparing hydrogels with varying polymer concentrations (*e.g.*, 2Ag_5.6_PAAc, 3Ag_5.6_PAAc, 3Ag_9_PAAc) through UV-initiated polymerization after heat-induced agarose gelation, and their properties were characterized using techniques such as tensile testing, swelling ratio measurement, zeta potential analysis, and liquid-phase atomic force microscopy. Research data showed significantly enhanced mechanical properties of the hydrogel, with 3Ag_5.6_PAAc exhibiting a fracture stress of up to 0.5 MPa. Its surface charge and microstructure changed regularly with ion concentration. However, its actuation mechanism originated from osmotic pressure differences caused by ion concentration gradients and the charge regulation effect of polyelectrolytes. This revealed the regulatory mechanism of the ionic environment on the microstructure, mechanical properties, and surface characteristics of charged double-network hydrogels within the agarose/poly(acrylic acid) system, as well as the universal law governing its charge regulation. The responsive behavior of double-network hydrogels is jointly determined by their composition and the ionic environment, and the dynamic regulation mechanism of their charge and microstructure opens new avenues for functional material design.^125^ However, in practical applications, hydrogel-based micro/nano-robotic medical devices must navigate autonomously in complex fluid environments, where non-uniform electric field distribution and variations in media conductivity can pose challenges.

#### 3.2.3. Optical drive

**Fig. 11:**
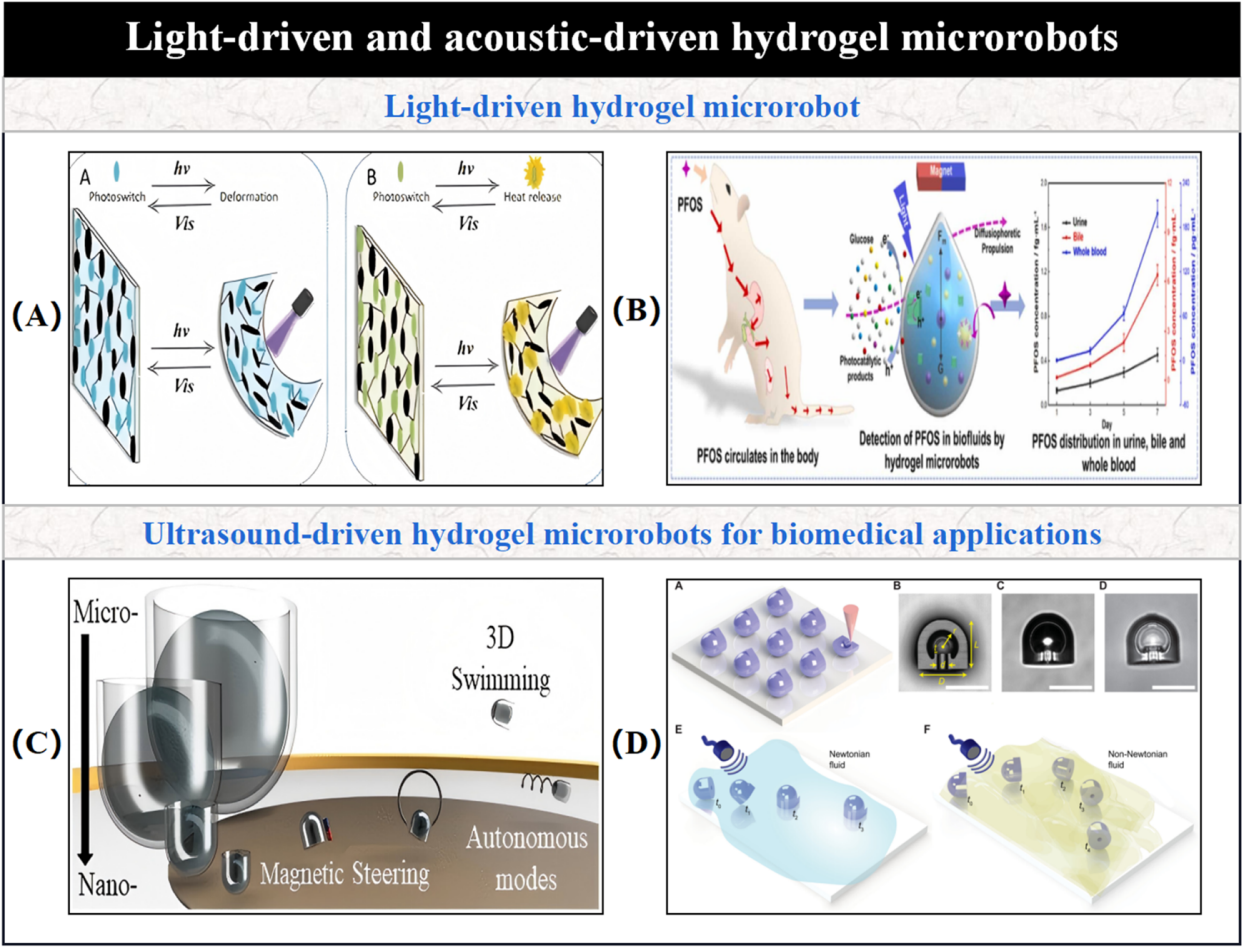
(A) Two different actuation mechanisms of photo-responsive materials. © 2024 The Author(s). Published by the Royal Society of Chemistry.^126^ (B) Visualized correlation between PFOS biodistribution patterns and micro/nano-robotic medical device detection principles in literature. © 2025 Elsevier B.V. All rights are reserved.^129^ (C) Ultrasound-propelled nanoswimmers penetrating and diffusing into tumor microvessels. © 2020 American Chemical Society.^130^ (D) Differential interaction mechanisms of acoustically-driven micro/nano-robotic medical devices with microscale structures in Newtonian and non-Newtonian fluids. © 2022 The Authors, some rights reserved.^131^

Photo-actuation drives hydrogel-based micro/nano-robotic medical devices by utilizing light's effect on photosensitive materials within the hydrogel. Upon absorbing photons, these photosensitive materials undergo photothermal effects, photochemical reactions, or photo-induced deformations, which can also enable drug release. Photo-actuation offers advantages such as rapid response speed, strong controllability, and remote control, making it suitable for applications in biomedical engineering and environmental science.^16^ Photo-actuation is primarily divided into photomechanical actuation and photothermal actuation. As shown in Fig. 11(A), a clear comparison is made between these two distinct actuation mechanisms of photo-responsive materials. The experimental systems are mainly based on two types of materials, such as thermosensitive hydrogels and liquid crystal elastomers, achieving photo-responsiveness by doping with photothermal conversion materials like gold nanoparticles, carbon nanotubes, or photomechanical switching molecules such as azobenzene. The actuation mechanisms are categorized into photothermal actuation, which converts light energy into heat to induce material contraction and expansion, and photomechanical actuation, which directly induces molecular conformational changes *via* light. Specific experiments include rapid bending and coiling of gold-coated PNIPAM strips within 90 ms under infrared light, LCE-based biomimetic snail-like crawling devices, and light-controlled micro-grippers for grasping and transporting objects. Photothermal actuation can achieve millisecond-level response times, while photomechanical actuation can realize complex three-dimensional deformations. Application areas include biomimetic technology, surface cleaning, and intelligent transportation. However, photo-actuated soft medical devices still face challenges such as low actuation force, expensive equipment, and limited application in opaque media.^126^ For example, Zhu *et al.* combined multi-step electric field alignment and photolithographic polymerization techniques to fabricate anisotropic hydrogels with complex patterned structures. Photothermal effects triggered rapid localized contraction and expansion of the hydrogel, while temperature concurrently modulated its friction coefficient. This technique was applied to a biomimetic soft medical device based on a poly(*N*-isopropylacrylamide) nanocomposite hydrogel, which achieves multi-modal locomotion through photo-actuation. The specific experimental system involved constructing anisotropic structures based on electric field-aligned fluorohectorite nanosheets and gold nanoparticles. Under 520 nm green light irradiation, rapid temperature changes could be triggered, with temperatures rising to 55 °C within 3 seconds, leading to anisotropic deformation: expansion by 1.5 times perpendicular to the nanosheet orientation and contraction to 0.8 times parallel to it. Experimental data indicated that this hydrogel could achieve crawling, walking, and turning movements on a PVC substrate *via* light scanning, with its locomotion mechanism relying on the dynamic regulation of photothermal deformation and friction coefficient.^120^ The bottleneck of the aforementioned hydrogel actuators lies in their reliance on water molecule diffusion mechanisms, which leads to slow actuation speeds, and complex movements are limited by material fabrication methods. Ni *et al.* proposed a thermosensitive hydrogel based on dynamic disulfide bond crosslinking, achieving high-speed, programmable actuation through photomechanical programming. UV light triggers the cleavage and recombination of disulfide bonds, introducing spatially selective network anisotropy that locally aligns the hydrogel chains. Actuation behavior is dominated by temperature-regulated polymer chain conformational changes, rather than traditional water diffusion mechanisms, significantly enhancing speed. The addition of carbon particles enables the photothermal effect for near-infrared light actuation, achieving a rapid temperature response.^127^ Concurrently, Deng *et al.* developed carbon nanotube-doped composite hydrogels, which significantly enhance the photosensitivity and response speed of hydrogel-based micro/nano-robotic medical devices by improving the light absorption rate, thermal conductivity, and mechanical modulus of the crosslinked network. Laser-induced *in situ* photocrosslinking of hydrogels enables one-step printing of 3D structures and programming of photo-responsive properties. This supports high-precision manufacturing of complex microstructures, with resolutions down to the sub-micron level. This method provides new insights for the large-scale manufacturing of photo-actuated hydrogel-based micro/nano-robotic medical devices, promoting their application in targeted drug delivery, minimally invasive surgery, and micro/nano engineering. Future extensions to other photosensitive material systems could further enhance actuation efficiency and functional integration.^70^ Furthermore, variations in light intensity can be utilized to promote precise control over movement and morphological adjustments. The rapid response and versatile adaptability of photo-responsive hydrogels pave the way for innovative applications. However, challenges persist, including nanoscale limitations in actuation force, expensive optical manipulation equipment, and insufficient light penetration in opaque media. From the locomotion control of hydrogel-based micro/nano-robotic medical devices to the dynamic shape regulation of wearable technologies, future efforts need to combine multi-field actuation to enhance three-dimensional control capabilities and expand their applications in biomedical and other fields.^121,126,128^ Dual-actuation synergy can effectively improve precision. As shown in Fig. 11(B), magnetic fields address deep tissue penetration, while photo-actuation enables precise localized energy delivery. Magnetic fields guide the hydrogel-based micro/nano-robotic medical devices to the target area, and near-infrared light irradiation activates the generation of photoelectrons, granting photo-actuated hydrogel-based micro/nano-robotic medical devices greater functionality through the combination of these two actuation methods.^129^

#### 3.2.4. Sound driver

Ultrasound actuation is one of the crucial actuation methods for propelling hydrogel-based micro/nano-robotic medical devices, with three main actuation strategies widely employed in the biomedical field. (1) Utilizing the acoustic radiation force generated when sound waves propagate through a medium, acting upon the surface of the hydrogel-based micro/nano-robotic medical device. For instance, the distribution of acoustic pressure nodes and anti-nodes in a standing wave field can exert periodic forces on structures like metal nanorods, compelling them to move along the acoustic pressure gradient. McNeill *et al.* developed a wafer-scale scalable manufacturing technique that addresses the limitations of traditional methods regarding size constraints, insufficient yield, and degrees of freedom in motion through acoustic actuation and magnetic control technologies.^130^ (2) When sound waves propagate in a fluid, they induce viscous shear forces, creating stable vortices or directed flow fields. Hydrogel-based micro/nano-robotic medical devices gain propulsive force through the interaction between their surfaces and the acoustic streaming. For example, by acoustically driving microbubble oscillations to generate high shear rate microfluidic fields, Aghakhani *et al.* addressed the challenge of propelling hydrogel-based micro/nano-robotic medical devices in complex biological fluids.^131^ (3) Designing micron-sized cavities on or within the surface of the device, utilizing ultrasound to excite periodic oscillations of bubbles (cavitation effect), and achieving propulsion through the recoil force generated by bubble contraction/expansion. For instance, Ren *et al.*'s nanoswimmer employed a half-capsule structure, fabricated using direct laser writing combined with metal layer deposition, with a hydrophobic surface treatment to capture bubbles. In a megahertz acoustic field, bubble oscillations generate two main forces: the secondary Bjerknes force, which attracts the nanoswimmer towards boundaries, and acoustic streaming, which provides propulsive force for movement.^132^ This technology is extendable to fields such as hydrogel-based micro/nano-robotic medical devices, targeted drug delivery, and biomanufacturing, and its biosafety can be further enhanced by incorporating biodegradable materials or magnetic nanoparticles in the future. Combining acoustic actuation with other external physical fields effectively enriches the functionality of hydrogel-based micro/nano-robotic medical devices. As shown in Fig. 11(C), this hydrogel-based micro/nano-robotic medical device features micro-scale swimmers and nano-scale swimmers in its left section, with micro-swimmers designed for tumor vessel penetration and nano-swimmers for blood–brain barrier crossing. The central section is a *U*-shaped magnetic control region, where the Fe_3_O_4_ layer is steered by Lorentz force, overcoming the limitations of acoustic propulsion. The right section is ultrasound-driven, executing three types of movement: circular, helical, and linear trajectories *via* ultrasound. This 3D design, incorporating structural hierarchy, functional mapping, and environmental identification, particularly highlights the breakthrough value of 50 nm swimmers combined with acoustic autonomy and magnetic guidance.^130^ Subsequently, to address adaptation to more complex environments, Kaynak *et al.* fabricated biocompatible polyethylene glycol diacrylate using two-photon polymerization 3D printing technology. Without the need for bubbles or magnetic materials, driven by acoustic resonance, they achieved acoustic frequency-addressable control of an all-hydrogel micro-system for the first time, opening new pathways for the development of micro-scale biomedical tools.^133^ To date, ultrasound-actuated hydrogel-based micro/nano-robotic medical devices have become a key technology for targeted drug delivery and precise therapy due to their non-invasiveness, high penetrability, and real-time imaging compatibility, requiring drug delivery or operation execution in a controlled manner at specific times and locations, tailored to individual patient differences.^134–136^ In Fig. 11(D), the differential interaction mechanisms of Newtonian and non-Newtonian fluids on micro-scale structures are systematically presented. Under ultrasound actuation, a hydrogel-based micro/nano-robotic medical device in a Newtonian fluid encapsulates a probe with blue fluid, penetrates a cavity, and then undergoes structural settlement and deformation. In a non-Newtonian fluid, it encapsulates a probe with yellow fluid, resulting in non-uniform deformation.^131^

### 3.3. Stimulus response mechanisms

#### 3.3.1. pH response

pH-responsiveness is a critical characteristic of hydrogels as smart materials, primarily owing to the reversible changes in their molecular structure and physicochemical properties with variations in environmental pH. This responsive mechanism in hydrogel-based micro/nano-robotic medical devices primarily manifests in two categories.

The first mechanism involves pH-induced hydrolysis of chemical bonds or decomposition of complexes. In this mechanism, pH can directly trigger the cleavage of specific covalent or coordination bonds within the hydrogel network, leading to the structural degradation of the material. This in turn enables drug release or the disintegration of the device. For example, in metal–organic framework materials like zeolitic imidazolate framework (ZIF-8), the metal–organic coordination bonds can undergo hydrolysis in acidic environments, causing the framework structure to collapse and release encapsulated cargo. Cao *et al.* utilized the pH-responsive degradation properties of their synthesized Fe@ZIF-8 nanoparticles to achieve efficient doxorubicin loading.^51^ Similarly, certain pH-sensitive covalent bonds, such as hydrazone and acetal bonds, can undergo hydrolysis under specific pH conditions, thereby enabling hydrogel degradation or site-specific drug release.^137^

The second, and more prevalent, mechanism is the reversible protonation/deprotonation of weak acidic or weak basic functional groups within the hydrogel network, triggered by changes in pH. This consequently affects the charge density, hydrophilicity, and inter-chain electrostatic interactions of the polymer chains, ultimately leading to hydrogel swelling or shrinking.^138^

Specifically, carboxyl-containing (–COOH) hydrogels, such as poly(acrylic acid) (PAAc) and its derivatives like poly(methacrylic acid) (PMAA) and acrylic acid–acrylamide copolymers (AAc–Am), contain numerous weakly acidic carboxyl groups on their polymer backbone. In acidic environments, carboxyl groups remain protonated, resulting in low charge density, lack of electrostatic repulsion between polymer chains, and increased hydrophobicity. This leads to water expulsion from the hydrogel interior and network contraction. In alkaline environments, carboxyl groups deprotonate to form negatively charged carboxylates, significantly increasing electrostatic repulsion between polymer chains and enhancing hydrophilicity, causing a large influx of water molecules into the network *via* osmosis, and hydrogel swelling. This mechanism is widely applied in controlled drug release, such as poly(methacrylic acid)-*g*-poly(ethylene glycol) hydrogel systems for anticonvulsant drug release.^139^ NIPAM/AA/MAA copolymer hydrogels utilize the carboxyl groups of acrylic acid (AA) and methacrylic acid (MAA) to achieve volume shrinkage at pH 7–5 and swelling in alkaline environments for drug loading and release.^140^ Agar-PAAc hydrogels also leverage PAAc carboxyl deprotonation-induced volume expansion combined with magnetic fields for actuation at pH > 6.^141^ Sodium alginate (SA), as a natural polysaccharide hydrogel, is also rich in carboxyl groups on its main chain. Similar to PAAc, SA's carboxyl groups undergo protonation/deprotonation with pH changes, conferring pH responsiveness. For example, Zhang *et al.* utilized this characteristic of sodium alginate to design pH-responsive hydrogels.^10^ In electrodeposition techniques, local pH changes induced by H^+^ or OH^−^ generated from water electrolysis can induce ionization of sodium alginate carboxyl groups, which in turn affects their ionic crosslinking with Ca^2+^, enabling precise molding of micron-scale structures.^76^ Double-network hydrogels containing itaconic acid (IA), such as the first network formed by itaconic acid and acrylamide (IA-PAAM), where the carboxyl groups of the itaconic acid portion undergo protonation/deprotonation with pH changes, directly affecting the degree of hydration. This results in the network exhibiting high swelling ratios and pH responsiveness, allowing it to conform closely to uneven surfaces.^29^ Amine-containing (–NH_2_) hydrogels typically contain weakly basic amine groups. In acidic environments, amine groups are protonated to form positively charged ammonium groups (–NH^3+^), leading to increased electrostatic repulsion between polymer chains and hydrogel swelling. In alkaline environments, amine groups remain deprotonated, reducing charge density and causing hydrogel contraction. Chitosan is a typical amine-containing pH-responsive hydrogel, where the protonation degree of its amine groups varies with pH, affecting its bioactivity and swelling behavior. The pH responsiveness of DNA hydrogels can be achieved in two ways: firstly, by introducing pH-sensitive nucleobases into the DNA sequence, where the protonation/deprotonation of these modified bases affects the stability of the DNA double helix or forms specific conformations, leading to changes in the hydrogel network; secondly, by covalently crosslinking pH-sensitive polymers to the DNA backbone, thereby imparting the pH-responsive characteristics of the external polymer to the DNA hydrogel, enabling controlled drug release. In poly(2-hydroxyethyl methacrylate)-methacrylic acid (PHEMA-MAA) copolymers, Li *et al.* designed a bilayer pH-responsive hydrogel where methacrylic acid was introduced into the PHEMA layer through copolymerization. The carboxyl groups in MAA protonate in the tumor's microacidic pH environment, leading to reduced hydrophilicity and contraction of the polymer chains, thus exposing drug loading sites for precise release.^142^ In ionized group polymer modified (HHPA) MOF hydrogel-based micro/nano-robotic medical devices proposed by Zhang *et al.*, HHPA typically refers to polymers containing ionizable groups. These ionizable groups can undergo charge reversal upon pH changes, altering the polymer's surface charge state to adapt to the pH environment, thereby promoting intratumoral accumulation and cellular uptake.^143^

pH-stimuli responsive hydrogel-based micro/nano-robotic medical devices are not only widely used in targeted cancer therapy and targeted drug delivery^10,142,144^ but also show great potential in assisted positioning and real-time monitoring. For example, Zheng Yu *et al.*'s swarm of magnetic photonic crystal hydrogel-based micro/nano-robotic medical devices, where the integrated pH-responsive hydrogel spheres (acrylic acid and acrylamide copolymer) swell and shrink in acidic microenvironments of pH 5.0–7.4. This leads to changes in the lattice constant of the one-dimensional Fe_3_O_4_ nanoparticle assembly, causing a shift in structural color from red to blue, thereby achieving real-time visual pH mapping and self-regulated drug release.^145^

In summary, pH-responsive hydrogels, due to their diverse chemical mechanisms and broad application prospects, represent a significant branch of hydrogel systems. Future research needs to further deepen the understanding of these molecular-level responsive mechanisms, and by combining them with other external physical conditions or stimuli-responsive elements, further optimize the performance and functionality of hydrogel-based micro/nano-robotic medical devices to address the needs for precise diagnosis and therapy in complex biological environments.

#### 3.3.2. Temperature response

**Fig. 12:**
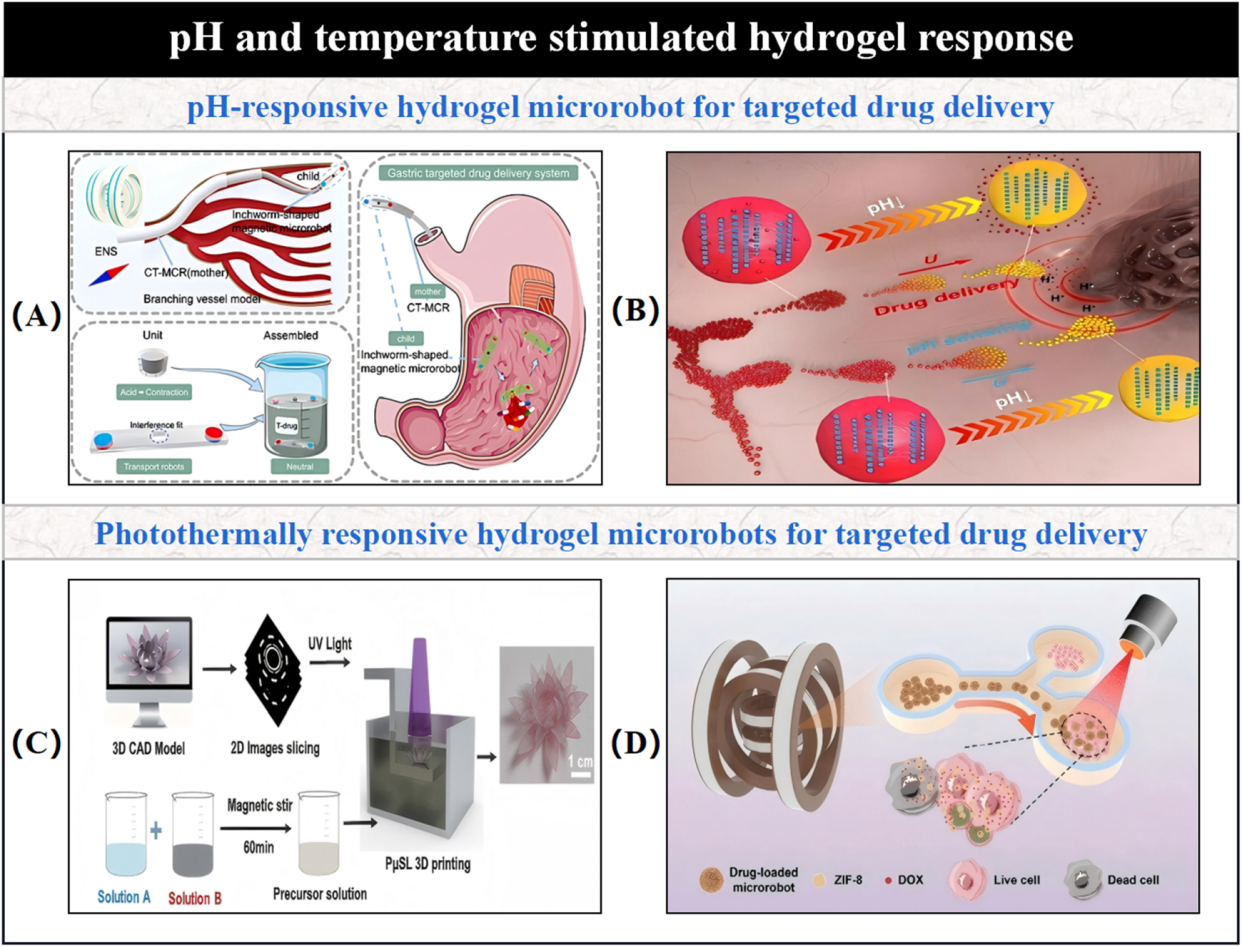
(A) Assembly principle of pH-responsive hydrogels with inchworm-like magnetically driven micro/nano-robotic medical devices, and a magnetically controlled mother-daughter micro/nano-robotic medical device system for targeted drug delivery in the stomach. © 2025 The Author(s). Published by Elsevier Ltd.^140^ (B) Magnetic field-driven locomotion and pH-visualizing real-time detection using micro/nano-robotic medical devices. © 2023 The Authors. *InfoMat* published by UESTC and John Wiley & Sons Australia, Ltd.^145^ (C) Photothermally responsive hydrogel-based micro/nano-robotic medical device system. © 2021 The Author(s). Published by IOP Publishing Ltd on behalf of the IMMT.^147^ (D) MOF-loaded biotemplated magnetic micro/nano-robotic medical device. © 2025 American Chemical Society.^148^

**Fig. 13:**
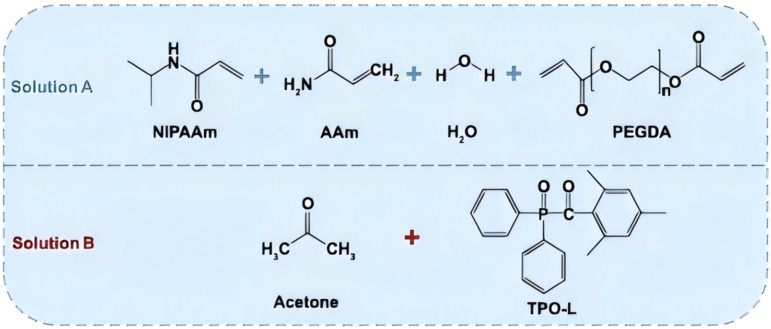
UV light excites TPO-L to generate free radicals, initiating NIPAAm/PEGDA polymerization and crosslinking, forming an intelligent responsive hydrogel network. © 2021 The Author(s). Published by IOP Publishing Ltd on behalf of the IMMT.^147^

Temperature, due to its ease of control, is one of the widely applied external stimuli. Critical Solution Temperature (CST) is an important parameter for hydrogels. Low Critical Solution Temperature (LCST) hydrogels exhibit a hydrophilic, extended conformation below their LCST, where polymer segments form stable hydrogen bonds with water molecules. As the temperature rises above the LCST, the hydrophobic interactions of PNIPAM chains intensify, leading to water expulsion, polymer chain aggregation, and contraction. High Critical Solution Temperature (UCST) hydrogels, such as *N*-acetylglucosamine (NAGA)-based hydrogels, contract as temperature decreases due to enhanced inter-chain hydrogen bonding.^146^ Similar to pH stimuli, temperature stimuli are also applied in both actuation and deformation. As shown in Fig. 12(C), Zhan proposed a photothermally responsive hydrogel based on *N*-isopropylacrylamide. The π-electron conjugated structure of carbon nanoparticles endows it with efficient near-infrared (NIR) light absorption capability, which is then converted into thermal energy, enhancing NIR absorption. Utilizing grayscale projection microstereolithography 3D printing technology, complex structures are manufactured with high precision, creating intelligent hydrogel precursor solution chemical compositions, as shown in Fig. 13. Deformation is completed within 1–5 seconds under NIR light irradiation, and the deformation angle can be precisely controlled. Non-contact motion control in aqueous environments is achieved by combining this with the Marangoni effect.^147^

Regarding actuation, two common types of temperature-driven mechanisms are frequently employed: those based on poly(*N*-isopropylacrylamide) (PNIPAM) and liquid crystal elastomers (LCEs). PNIPAM-based systems achieve actuation through morphological changes involving contraction and expansion, while liquid crystal elastomers move due to their intrinsic structural anisotropy or disorder. For instance, Xie *et al.* developed a temperature-responsive double-network hydrogel constructed with polyvinyl alcohol as the first network and polyacrylamide combined with poly(*N*-isopropylacrylamide) as the second network. This hydrogel exhibited significant temperature-responsive behavior, with a swelling ratio reaching 257% at 30 °C and decreasing to 58% at 60 °C. Its phase transition temperature was approximately 34 °C, and it achieved a 30° bending angle within a 2-second response time, demonstrating rapid and reversible deformation capability. Based on the low critical solution temperature (LCST) characteristic of PNIPAM, the hydrogel undergoes reversible swelling and contraction changes upon temperature stimulation, making it suitable for constructing soft actuators. By designing a bilayer structure, the hydrogel can undergo bending deformation under temperature changes due to differences in swelling ratios between its two layers, thereby achieving thermo-driven actuation. This material possesses rapid responsiveness, programmable deformation, and good cyclic stability, showing potential in the field of micro-scale soft robotics, for example, as a temperature switch or a thermo-driven actuator for environmental sensing and deformation control.^149^

In terms of deformation-mediated targeted drug release and targeted therapy, temperature responsiveness often appears in conjunction with light, where hydrogels exhibit photothermal responses upon reaching a specific location. The photothermal response of a hydrogel refers to its ability to convert light energy into thermal energy *via* photothermal agents under illumination, which subsequently triggers dynamic changes in material structure, properties, or functions such as crosslinking density and pore size.^16^ For example, Chen *et al.* developed a bifunctional hydrogel system with both self-actuation and self-monitoring capabilities, combining photothermal actuation with resistance sensing. In this system, the photothermal response is mediated by the unique electronic structure of graphene nanosheets, enabling efficient absorption of light energy and its conversion into thermal energy. This localized temperature increase triggers the LCST phase transition of PNIPAm (approximately 32 °C), leading to hydrophobic aggregation of PNIPAm chains and dehydration-induced shrinkage or swelling of the hydrogel network.^150,151^ In Fig. 12(D), Gu *et al.* utilized a three-tier strategy involving “biotemplating-MOF drug loading-dual responsive release”, combining magnetic actuation with photothermal reactions to achieve chemo-photothermal synergistic therapy and pH/light dual-triggered drug release, leading to intelligent responses in the tumor microenvironment. Specifically, under the low pH conditions of the tumor microenvironment, the MOF structure may undergo cleavage or reconstruction of its coordination bonds, leading to drug release. Simultaneously, photothermal materials generate heat under light irradiation, accelerating drug release and/or inducing hyperthermic effects.^148^

Temperature-responsive hydrogels can be integrated with other stimulus-responsive and actuation mechanisms to achieve synergistic effects between temperature and other stimuli, thereby enhancing the performance and functionality of hydrogel-based micro/nano-robotic medical devices.

### 3.4. Multi-mechanism synergy (multifunctionality)

In recent years, hydrogel-based micro/nano-robotic medical devices have found applications in various fields. However, due to the complex and variable environments these devices encounter and their typical responsiveness to only a single stimulus, achieving truly precise control remains challenging. Consequently, research integrating multiple actuation mechanisms has progressively become a focal point, with composite hydrogels emerging as crucial carriers for various functional materials and essential materials for realizing multifunctional hydrogel-based micro/nano-robotic medical devices. Typically, the mechanism for multi-stimuli responsive hydrogel-based micro/nano-robotic medical devices involves integrating various responsive materials with distinct functionalities. This approach focuses on assembling diverse functional components to achieve a broad spectrum of capabilities. The actuation mechanisms involving multi-component assemblies in these devices enable them to respond to multiple external stimuli. This entails integrating various multifunctional components to react to a range of stimuli, rather than being limited to a single one, and designing these devices appropriately to ensure their responsiveness to diverse external cues.

#### 3.4.1. Integration of driving mechanisms with stimulus response mechanisms

**Fig. 14:**
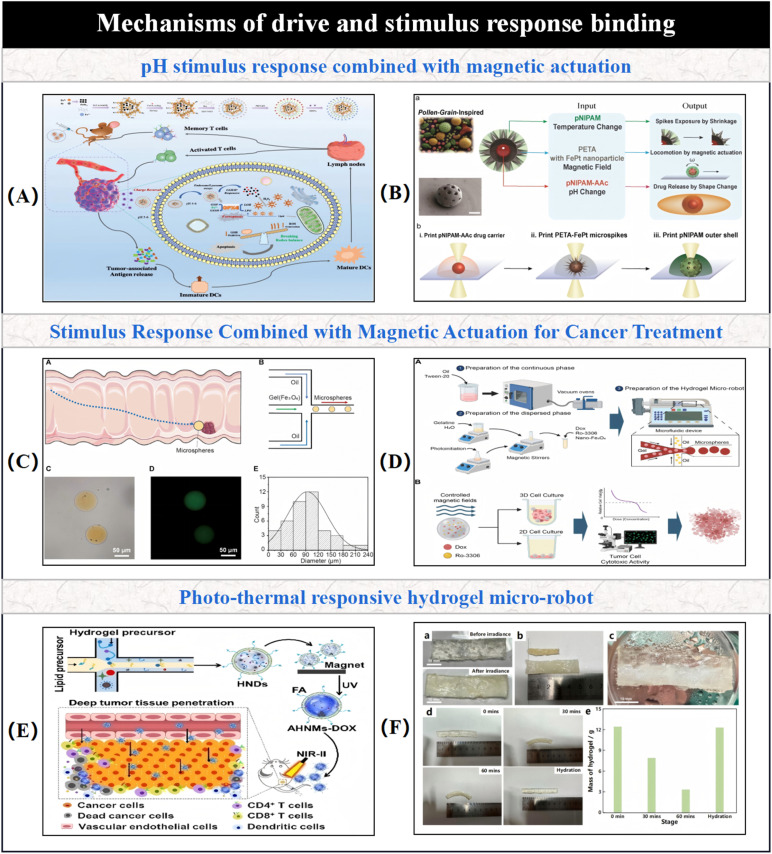
(A) Precise theranostics of hydrogel-based micro/nano-robotic medical devices. © The Royal Society of Chemistry 2022.^143^ (B) Pollen-inspired multifunctional hydrogel-based micro/nano-robotic medical devices, enabling independent control of locomotion, anchoring, and drug release. © 2023 The Authors. *Advanced Materials* published by Wiley-VCH GmbH.^65^ (C) Targeted delivery system of magnetic fluorescent microspheres, encompassing the full chain of preparation, characterization, and application validation. © 2024 Jiang, Zheng, Zhao, Qi, Wu, Li, Wu and Han.^7^ (D) Synthetic lethality strategy using magnetic hydrogel-based micro/nano-robotic medical devices to enhance osteosarcoma chemotherapy. © 2024 Tao, Li, Yang, Yin, Zhang, Wang, Pu, Wang, Zhang, Mu, Wu, He, and Yang.^18^ (E) Enhanced immunochemotherapy by NIR-II light-driven asymmetric hydrogel-based nanodevices. © 2022 Wiley-VCH GmbH.^152^ (F) Multifaceted performance changes of photo-responsive smart hydrogels under light and humidity stimuli. © 2024 by the authors.^153^

The combination of external stimuli such as light, magnetic fields, and electric fields with internal stimuli like pH, temperature, and ions represents the most widely adopted multi-actuation approach. This integration enables hydrogel-based micro/nano-robotic medical devices to respond simultaneously to changes in both *in vitro* and *in vivo* environments, thereby achieving precise control and efficient functionality.^6–8,10,16,18,62,65,104^ Regarding the combination of pH response and magnetic actuation, as shown in Fig. 14(A), Zhang *et al.* developed an iron-based smart hydrogel-based nanodevice for synergistic tumor therapy by combining chemodynamic therapy (CDT), photodynamic therapy (PDT), and immunotherapy. The specific experimental system utilized FeSe_2_ nanoparticles embedded in a MOF structure and modified with HA/PEI/CpG@HHPA, forming 210 nm nanogels. These nanogels undergo charge reversal in the acidic tumor microenvironment (pH 5.8), releasing Fe ions and the Ce_6_ photosensitizer. Experimental data revealed over 78% selenium ion release under pH 5.8 and GSH conditions. The combined CDT/PDT treatment demonstrated an IC_50_ of 40 μg mL^−1^ against 4T1 cells *in vitro* and significantly reduced intracellular GSH levels. *In vivo* treatment resulted in a substantial increase in tumor volume inhibition. The *in vivo* studies showed a blood half-life of 1.5 hours, with most of the nanodevice cleared after 8 hours, and iron ion enrichment at the tumor site. Experiments also indicated downregulated GPX4 expression and increased lipid peroxidation product MDA. Immunogenicity analysis confirmed an increase in splenic CD8^+^ T cells to 29.18% post-treatment, significant elevations in serum IL-6, IFN-γ, and TNF-α levels, and induced memory T cell formation, signifying a potent immune activation effect. This multi-actuation nanosystem demonstrated synergistic therapeutic potential by disrupting intracellular redox balance and activating immune responses.^143^ Similarly, in Fig. 14(B), Lee *et al.* developed a 3D-printed multifunctional pollen-inspired hydrogel-based medical device. Through a combination of multiple actuation mechanisms, it achieves directional movement, anchoring, and drug release. FePt nanoparticles embedded within PETA spike structures enable magnetic actuation for surface rolling, reaching speeds of up to 532 μm s^−1^. Temperature triggers anchoring functionality, with a 49% shrinkage at 45 °C exposing the spikes. pH controls drug release, with a 300% swelling at pH 11 releasing 70% of a fluorescent dye. Specific experimental data indicated an anchoring force of 1.2 mN and 2.8 mN on pig intestinal tissue, significantly exceeding simulated blood flow disturbances.^65^

In biomedical applications, the combination of magnetic field-driven actuation and pH-stimulus response is one of the most common synergistic approaches. By embedding magnetic nanoparticles within hydrogels, hydrogel-based micro/nano-robotic medical devices can achieve directional movement under an external magnetic field. Concurrently, by designing hydrogel materials with pH-sensitive functional groups, these devices can undergo protonation/deprotonation of polymer chains, leading to conformational or volumetric changes, or drug release, in different pH environments. For instance, Zhang proposed a metal–organic framework (MOF)-based intelligent hydrogel-based nanodevice. Modified with HHPA (typically referring to polymers containing ionizable groups), it achieves pH-responsive charge reversal, adapting the polymer surface charge state to the pH environment to promote intratumoral accumulation and cellular uptake.^143^ Subsequently, Cao *et al.* synthesized magnetic Fe@ZIF-8 nanoparticles *via* a one-step method, which possess high specific surface area, superparamagnetism, and pH-responsive degradation characteristics, enabling efficient doxorubicin loading.^51^ The same principle applies to cancer treatments discussed previously.^7,18^ Lee *et al.* fabricated hydrogel-based micro/nano-robotic medical devices using 3D microprinting technology, achieving decoupled multifunctionality including magnetic-driven locomotion, temperature-responsive anchoring, and pH-responsive drug release. Inspired by the nanospike morphology and cavity structure of pollen grains, these devices are composed of three hydrogel components: a magnetic layer containing iron-platinum nanoparticles, used for magnetic field response to achieve surface rolling and steering; a temperature-responsive layer, where a thermosensitive polymer in the outer shell undergoes a hydrophobic phase transition and contracts when the temperature exceeds its critical solution temperature, thereby exposing spikes to anchor to biological tissues; and a pH-responsive layer in the inner cavity, which actively releases drugs through protonation/deprotonation of polymer chains as pH changes.^65^ In the context of cancer therapy combining stimulus response and magnetic actuation, as shown in Fig. 14(C and D), Jiang *et al.* demonstrated a magnetic-photonic dual-functional integration, synchronously achieving magnetic-driven motion tracking and fluorescent efficacy feedback, delivering a plant-derived anticancer drug, offering a new therapeutic paradigm for colorectal cancer. Similarly, magnetic iron oxide nanoparticles (Fe_3_O_4_) overcome biological barriers such as blood flow shear forces under an external magnetic field to facilitate the delivery of cancer drugs.^7,18^

Another common synergistic combination is photo-actuation and temperature response. The integration of photo-actuation and temperature response essentially leverages the photothermal effect as a “bridge”, converting non-invasive light energy into a controllable localized thermal field and combining with the specific structure of the hydrogel-based micro/nano-robotic medical device, thereby precisely triggering the intelligent deformation or functional response of the hydrogel. For instance, Wang *et al.* utilized near-infrared region II (NIR-II) light to actuate nanomotors, combining the high photothermal conversion efficiency of copper sulfide to endow the nanomotors with anisotropic photothermal response, achieving deep tissue penetration and targeted therapy.^152^ Similarly, Zhan *et al.* combined the photothermal effect of near-infrared (NIR) light with grayscale-controlled 3D printing to achieve ultrafast response and controllable deformation of hydrogels. NIR light excites carbon nanoparticles to generate heat, inducing hydrogel network contraction and rapid water release, with water absorption for shape recovery after the light source is turned off.^147^ As shown in Fig. 14(E and F), Liu *et al.* leveraged the strong near-infrared light absorption at 1064 nm and high photothermal conversion efficiency of copper sulfide in Janus hydrogel-based nanomotors to achieve a trifunctional coupling of “photo-driven locomotion + immune regulation + chemotherapeutic drug release”. High-throughput fabrication was achieved by combining microfluidic chips with UV photopolymerization. A five-dimensional data chain revealed the synergistic regulatory capability of photo-responsive hydrogels in terms of deformation and mass, endowing the hydrogel-based micro/nano-robotic medical devices with photothermal responsiveness enabled by photothermal conversion.^152,153^ Subsequently, Liu and Zheng proposed a caterpillar-inspired bilayer hydrogel-based micro/nano-robotic medical device with added Fe_3_O_4_ nanoparticles. These nanoparticles act as photothermal agents under illumination, enhancing the efficiency of light-to-heat conversion. Simultaneously, their thermal conductivity aids in rapid heat diffusion within the hydrogel, thereby accelerating the hydrogel's thermal response, leading to faster response times and achieving rapid ground locomotion through thermal response.^153^ Beyond the combination of external magnetic fields and pH, Lai *et al.* developed a hybrid actuator combining a pH-responsive hydrogel with magnets, where both pH responsiveness and magnets contribute to driving the hydrogel-based micro/nano-robotic medical device's movement. Utilizing agar-poly(acrylic acid) hydrogels, at pH > 6, the carboxyl groups of poly(acrylic acid) deprotonate, causing the polymer chains to become charged, increasing electrostatic repulsion, resulting in significant volume expansion and generating blocking stress. By combining this with the repulsive force of permanent magnets, a hybrid actuator was designed, analogous to a spring, where the hydrogel's expansion triggers the rapid movement of a magnetic mechanism. This design offers a novel passive actuation solution for miniature medical hydrogel-based micro/nano-robotic medical devices, and future improvements could involve optimizing hydrogel chemical composition or crosslinking strategies to reduce response time.^141^ Such strategies possess unique advantages in minimally invasive medicine, targeted delivery, and soft robotics, and represent an important technological pathway for achieving high-precision, multi-functional hydrogel-based micro/nano-robotic medical devices.

#### 3.4.2. Combination of multiple drive mechanisms

**Fig. 15:**
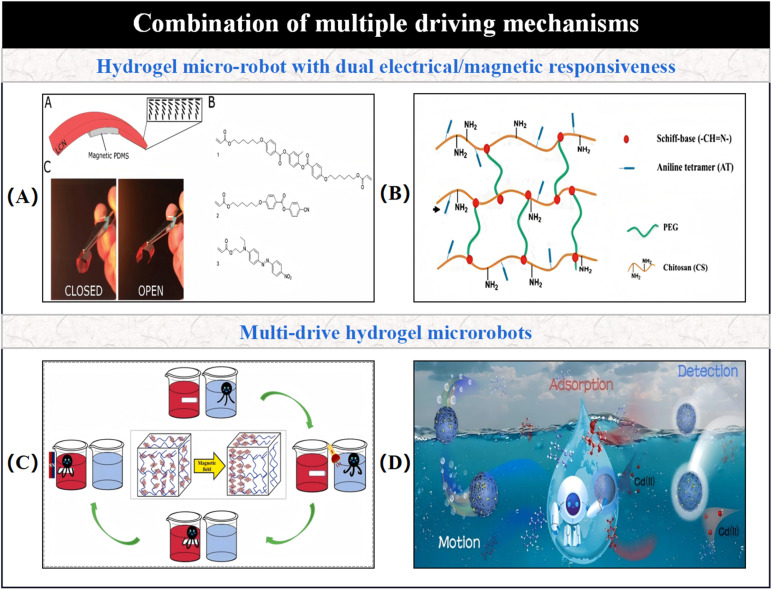
(A) Structural design, material composition, and function of a magnetically driven liquid crystal elastomer actuator. © 2019 The Authors.^154^ (B) Structural design of a chitosan-poly(ethylene glycol)-aniline tetramer composite hydrogel. © 2022 The Authors.^155^ (C) Asymmetric structured multi-responsive hydrogel actuator, with thermal/near-infrared/magnetic tri-modal actuation. © 2023 Elsevier B.V. All rights reserved.^102^ (D) Low-cost sodium alginate hydrogel-based micro/nano-robotic medical device. © 2025 Elsevier Ltd. All rights are reserved.^160^

The actuation technology for hydrogel-based micro/nano-robotic medical devices has evolved from single physical field actuation to multi-field synergistic actuation, aiming to enhance their adaptability and functional diversity in complex environments. By integrating the advantages of different physical fields, their application potential in biomedical, environmental, and other fields has been significantly elevated. This combination of multiple actuations can provide higher flexibility and precision, allowing hydrogel-based micro/nano-robotic medical devices to function effectively in various scenarios.^102,154–158^ For example, Pilz da Cunha *et al.* developed a dual-responsive soft gripper composed of photo-responsive liquid crystal polymers and magneto-responsive composite materials, where photo-actuation and magnetic actuation are combined, with each responsible for different functions. Photo-actuation controls the opening and closing of the gripper through bending induced by the azobenzene photothermal effect, while magnetic control achieves remote manipulation by guiding the movement or rotation of the PDMS/iron layer *via* an external magnetic field, as shown in Fig. 15(A) and (B). Both achieve remote control through the integration of magnetic nanoparticles. The first part discusses synthetic methods for magnetic hydrogels such as *in situ* precipitation and covalent bonding, while the second part achieves magnetic responsiveness through a PDMS/iron powder composite layer, both utilizing magnetic fields for precise manipulation.^154,155^ Composite hydrogels can also synergistically combine magnetic and electric fields. Garcia-Torres imparted electro-responsiveness to hydrogels by adding conductive nanomaterials such as metal nanoparticles, carbon nanotubes, conductive polymers, or by constructing conductive networks. Magnetic field responsiveness is achieved by embedding magnetic nanoparticles, with fabrication methods including physical mixing, *in situ* synthesis, and covalent bonding. Hydrogels with dual electro/magnetic responsiveness are widely used in biosensing and combined therapies.^155^ By incorporating magnetic nanoparticles into hydrogel-based micro/nano-robotic medical devices, they can be positioned and controlled under the influence of an external magnetic field. Concurrently, under specific light conditions, the morphological changes, movement, or drug release of these devices can be controlled. This combined approach is particularly suitable for deep tissue targeted therapy and for disease diagnosis and treatment requiring precise manipulation. Zhang *et al.* developed a multi-responsive hydrogel actuator based on an asymmetric structure, possessing triple actuation capabilities (thermo-responsive, near-infrared light-responsive, and magnetic-responsive). The asymmetric structural design enhances the hydrogel's actuation performance, integrating three actuation modes (thermal/NIR/magnetic) into a single material system, thereby improving environmental adaptability and application flexibility, as shown in Fig. 15(C) and (D). Magnetic fields can directionally control molecular arrangement, upgrading material functionality from disordered to ordered states.^102,159^ Yoon *et al.* fabricated an anisotropic bilayer hydrogel actuator, achieving multi-stimuli responsiveness (photoelectric-magnetic), multi-remote actuation, high strength, rapid response, and programmable complex deformation.^156^

While some hydrogel-based micro/nano-robotic medical devices possess excellent autonomous movement and navigation capabilities, making them suitable for drug delivery to hard-to-reach areas within the body. However, once these exogenous biohybrid micro/nano-robotic medical devices enter the body, they are highly susceptible to recognition, attack, and clearance by the immune system, thereby limiting their clinical applicability. Achieving targeted drug delivery in humans using micro/nano-robotic medical devices faces the critical challenge of immune rejection. Zhang *et al.* presented cutting-edge research on biohybrid micro/nano-robotic medical devices, centered on the development of a novel neutrophil-based micro/nano-robotic medical device for active targeted drug delivery to treat malignant brain tumors. Its interior consists of nanogels containing magnetic material, enabling remote guidance and manipulation by an external magnetic field for precise navigation. Neutrophils inherently possess natural chemotaxis; *in vivo*, they are attracted by chemical signals released from tumor or inflammatory sites, autonomously migrating towards the target area. The combination of these two mechanisms forms a synergistic active targeting strategy of magnetic navigation assistance and bio-autonomous chemotaxis, significantly enhancing delivery efficiency and precision. Through bacterial membrane cloaking and cell autophagy methods, inherent human neutrophils are engineered into micro/nano-robotic medical devices capable of performing active targeted drug delivery tasks. These micro/nano-robotic medical devices possess dual actuation and responsive capabilities of magnetic guidance and bio-autonomous chemotaxis, and have successfully addressed the critical challenge of immune rejection.^161^

## 4. Biomedical applications of hydrogel-based micro/nano-robotic medical devices

**Fig. 16:**
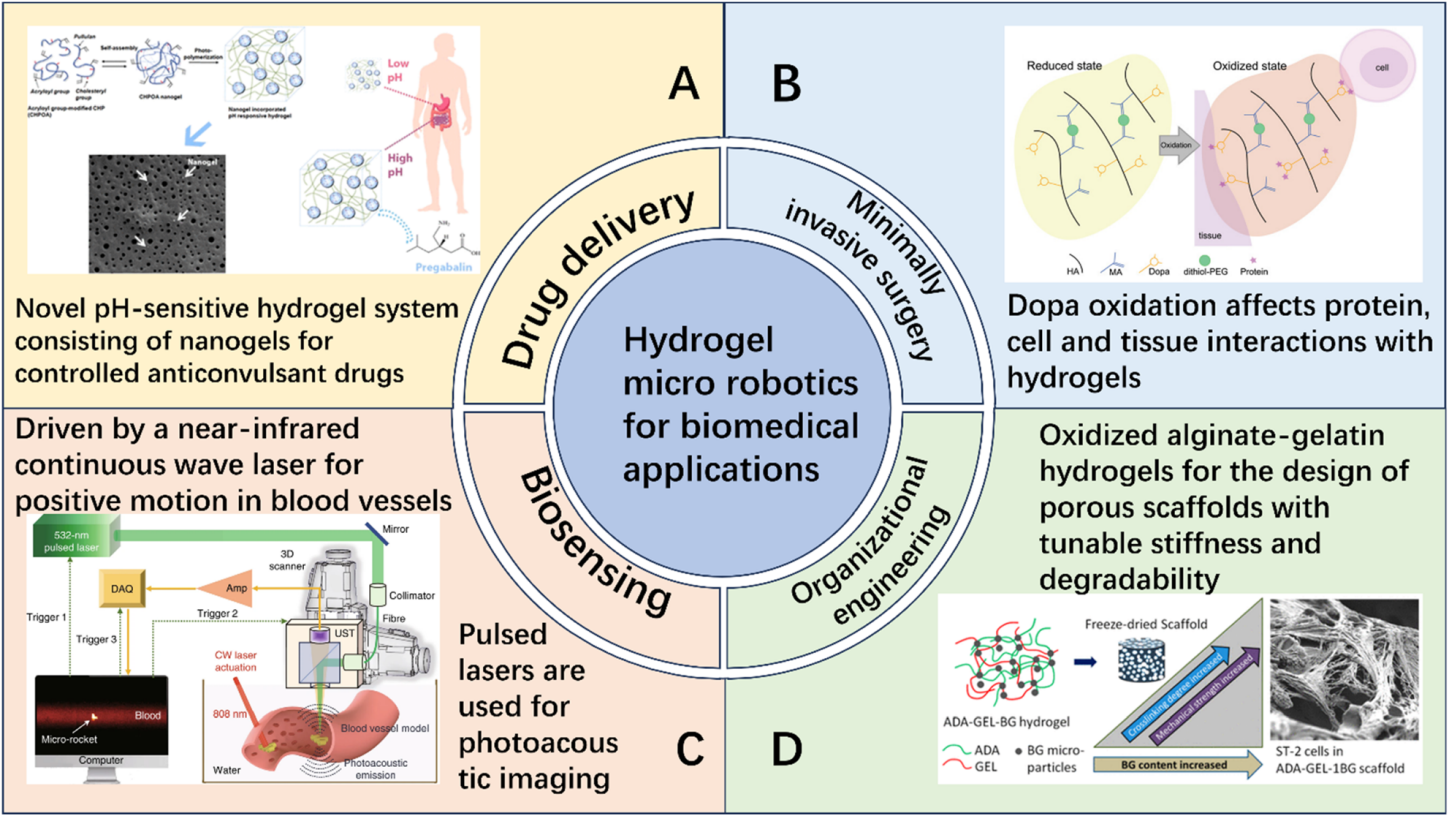
Biomedical applications of hydrogel-based micro/nano-robotic medical devices. (A) A novel pH-sensitive hydrogel system composed of poly(methacrylic acid)-*g*-poly(ethylene glycol) and acryloyl-modified cholesterol-containing pullulan nanogels for controlled release of anticonvulsant drugs. © 2017 American Chemical Society.^139^ (B) DOPA oxidation influencing interactions between proteins, cells, tissues, and hydrogels. © 2020 The Authors. Published by WILEY-VCH Verlag GmbH & Co. KGaA, Weinheim.^162^ (C) A hydrogel-based micro/nano-robotic medical device propelled by a near-infrared continuous wave laser for forward motion in blood vessels. A pulsed laser is used for photoacoustic imaging. © 2020, The Author(s).^163^ (D) Porous scaffolds composed of bioactive glass incorporated into covalently crosslinked oxidized alginate-gelatin hydrogels, designed using freeze-drying technology to achieve tunable stiffness and degradability. © 2016 American Chemical Society.^164^

Hydrogel-based micro/nano-robotic medical devices, owing to their high biocompatibility, programmable responsiveness, environmental adaptability, and ability to deform in response to multiple stimuli, enable them to adapt to various complex *in vivo* biological environments. Their applications in the medical field are becoming increasingly widespread. As shown in Fig. 16, the biomedical applications of hydrogel-based micro/nano-robotic medical devices are particularly prominent in targeted drug delivery, minimally invasive surgery, biosensing, and tissue engineering.

### 4.1. Targeted drug delivery

Targeted drug delivery stands as one of the pivotal applications of hydrogel-based micro/nano-robotic medical devices in medicine. By precisely delivering therapeutic agents to specific sites, particularly tumors, inflamed regions, or damaged tissues, these devices can significantly enhance treatment efficacy while reducing side effects. As depicted in Fig. 16(A), a novel pH-sensitive hydrogel system, comprising poly(methacrylic acid)-*g*-poly(ethylene glycol) and acryloyl-modified cholesterol-containing pullulan nanogels, is utilized for the controlled release of anticonvulsant drugs.^54,140,165–171^ Hydrogel particles, especially microspheres, are commonly used drug carriers due to their excellent modifiability, making them widely adopted in drug delivery systems. This enables them to serve as controlled release mechanisms that enhance drug bioactivity and stability. Hydrogel microspheres can serve as essential constituent materials for hydrogel-based micro/nano-robotic medical devices, augmenting their efficiency in drug delivery. For instance, Liu *et al.* described a micro/nanocomposite hydrogel microsphere that integrates various nanomaterials, such as targeted drug carriers, imaging probes, and biosensors, combining the flexibility of hydrogels with the functionality of nanomaterials. Depending on the loaded materials, these can perform diverse tasks, including efficient drug delivery, responsive release, detection of disease biomarkers, or promoting cartilage and vascular regeneration by loading growth factors or stem cells. The porous network structure of hydrogels can effectively encapsulate drugs and provide sustained, controlled release, which is crucial for achieving favorable pharmacokinetic properties. Sustained release directly influences drug absorption, distribution, metabolism, and excretion processes within the body. By regulating the hydrogel's crosslinking density, degradation rate, and the properties of the nanomaterials, stimulus-responsive release systems can be designed to optimize drug concentration–time profiles, reduce dosing frequency, and improve patient adherence. Precise drug delivery to the target enhances therapeutic efficacy significantly. Simultaneously, this system may possess the potential for real-time monitoring of treatment effects or biomarker changes. Given the generally good biocompatibility of hydrogels, this forms the basis for low immunogenicity.^165^ Shen *et al.* developed and validated a differentiable biohybrid robotic system for long-distance delivery of therapeutic agents in the human body, ultimately aiming for nerve connection repair. The robotic system is not composed of a single material or structure. It combines magnetic nanoparticles for propulsion and manipulation, biocompatible polymer hydrogels as synthetic materials, and bioactive components such as stem cells, growth factors, and exosomes for therapeutic purposes. By loading cells with differentiation potential, these cells, after precise delivery to the injury site by the hydrogel-based micro/nano-robotic medical device, can differentiate into the required neural cell types, thereby repairing severed neural circuits. This long-distance delivery strategy addresses the critical challenge in the field of micro/nano-robotic medical devices of effectively guiding and controlling these devices within the vast scale of the human body.^172^ Compared to hydrogel microspheres, hydrogel-based micro/nano-robotic medical devices offer the advantage of more precise drug delivery to target locations, and even the recovery of residual material after delivery. For example, Zou *et al.* innovatively proposed a magnetically driven mother-daughter robotic system, consisting of a magnetic continuum mother device and bioinspired inchworm-like magnetic micro-daughter devices. The mother device employs a multi-segment concentric tube structure and a detachable magnet design, achieving end-point constraint effects through stiffness differences, significantly enhancing navigation safety and operational space. The daughter devices incorporate pH-responsive hydrogels, allowing drug release to be triggered in acidic environments and complex path movements to be achieved *via* magnetic control. *In vitro* gastric model experiments verified that the system can complete the entire process of drug delivery, release, and recovery. By constructing an operational channel and a magnetic recovery mechanism, this system effectively reduces the risk of foreign body residue in biological systems.^140^ For gastrointestinal (GI) drug administration scenarios, the quantitative impact of physiological barriers must be thoroughly considered. Firstly, the GI tract exhibits a significant pH gradient: the gastric lumen pH can be as low as 1.5, gradually increasing to 6.0–7.4 in the small intestine, and potentially reaching 8.0 in the colon.^140^ This range not only influences the swelling/contraction behavior of pH-responsive hydrogels but can also prematurely or delay drug release. Secondly, the viscosity of the GI mucus layer is high; experimental measurements show it can exceed 10 Pa s under static conditions, far greater than aqueous media.^140^ High viscosity environments will significantly increase the drag coefficient *ζ* of hydrogel-based micro/nano-robotic medical devices within the mucus network, leading to reduced locomotion speed per unit energy, and the mucus turnover rate demands specific considerations for long retention strategies. Therefore, the design of hydrogel-based micro/nano-robotic medical devices for oral or rectal administration must not only optimize their surface interactions to reduce locomotion resistance in highly viscous mucus, such as using hydrophilic neutral polymer surfaces to minimize adhesion, but also regulate pH response thresholds, crosslinking density, and external actuation parameters to ensure the devices maintain sufficient maneuverability and controllable release kinetics in gradient pH and high-resistance fluid environments. Combining these physiological parameters for *in vitro*–*in vivo* validation can significantly enhance the predictability and reliability of GI drug delivery systems.^140^ In optimizing the predictability and reliability of GI drug delivery systems, there is an urgent need to establish precise dose-exposure-effect relationships. For instance, different dosing metrics, such as the number of hydrogel-based micro/nano-robotic medical device particles or drug load (particles per kg), the retention time of the devices in various GI segments, their degradation rates, and the accumulated drug concentration in target areas (*e.g.*, ulcer sites) (local *C*_max_ and AUCtarget) should be quantified. Simultaneously, it is necessary to clarify how drug release kinetics are affected by GI pH gradients and viscosity changes, which in turn influences local drug exposure levels. By establishing these key PK parameters and combining them with the latest biopharmaceutical PK/PD models and preclinical/early clinical data analysis methods, it will be possible to accurately assess whether the drug concentrations achieved by the devices under specific dosing regimens can reach therapeutic thresholds, and to establish a direct causal link with observed therapeutic effects, thereby better defining their therapeutic window. This is crucial for advancing hydrogel-based micro/nano-robotic medical devices to the clinical trial stage and requires strict adherence to relevant regulatory science guidelines to improve the success rate of clinical trials.^59^

Hydrogel-based micro/nano-robotic medical devices are capable of regulating drug release rates in response to external or internal stimuli. By operating within specific environments, these devices can achieve timed and quantitative drug release. The ability to adjust drug release enables hydrogel-based micro/nano-robotic medical devices to address the issue of uncontrolled drug release in traditional magnetic-responsive materials. For instance, Sun *et al.* proposed a soft capsule based on hard magnetic elastomer foam (HEF) for magnetically controlled on-demand drug delivery. Porous structures were prepared using sugar particles as templates, and the HEF was magnetized in a compressed state to enhance the magnetic field-driven compressive deformation rate. The HEF capsule structure features an inner HEF layer and an outer elastomer shell, which improves biocompatibility and reduces passive drug diffusion. It exhibits a higher drug loading capacity and allows for magnetically controlled release rates.^166^ Hydrogel materials inherently possess large specific surface areas and hydration properties, enabling efficient drug loading while maintaining drug biological stability and release efficacy. Furthermore, hydrogels can modify their chemical properties and structures, such as hydrophilicity/hydrophobicity and ionization degree, to adjust drug release performance. Han *et al.* proposed a biodegradable acoustic hydrogel-based micro/nano-robotic medical device, composed of gas-filled hydrogel, combining acoustic responsiveness and biodegradability. It achieves precise *in vivo* navigation through the synergistic action of sound waves and magnetic fields. Its effectiveness in delivering chemotherapeutic drugs was validated in a mouse bladder tumor model, significantly inhibiting tumor growth. After completing its task, the hydrogel naturally degrades *in vivo* through hydrolysis or enzymatic degradation, avoiding the risk of long-term retention. This addresses the pain points of difficult real-time tracking and long-term retention side effects associated with traditional micro/nano-robotic medical devices. The dual-mode actuation strategy combining acoustics and magnetic fields enhances manipulation capabilities in complex physiological environments.^6^ The excellent biocompatibility and biodegradability of hydrogels minimize irritation and damage to normal tissues during drug delivery, significantly improving therapeutic efficacy while effectively reducing potential drug side effects.

Hydrogel-based micro/nano-robotic medical devices exhibit significant characteristics in the field of targeted drug delivery. Their surface modification with specific ligands, such as antibodies or peptides, enables precise binding to receptors (*e.g.*, proteins or glycans) on the surface of diseased cells through highly specific molecular recognition, thereby effectively achieving targeted drug delivery. In the context of cancer therapy, hydrogel-based micro/nano-robotic medical devices can precisely deliver chemotherapeutic drugs into tumor cells. By ingeniously encapsulating magnetic nanoparticles and anticancer drugs within hydrogels, and guided by an external magnetic field, these devices accurately reach tumor sites. Subsequently, through temperature-induced polymer phase transitions or pH-responsive mechanisms affecting polymer ionization and swelling, they release drugs, achieving highly efficient killing of tumor cells and opening new avenues for cancer treatment.^7,18,142,144^ It is important to note that the targeting mechanisms of hydrogel-based micro/nano-robotic medical devices at tumor sites can be broadly categorized into passive enrichment (EPR effect) and active magnetic navigation, with their applicability closely related to tumor type and vascular permeability. Passive EPR (Enhanced Permeability and Retention) effect relies on structural defects in tumor vasculature, such as loose endothelial cell junctions, incomplete basement membranes, and impaired lymphatic drainage, which allow nanoscale carriers to passively extravasate into the tumor interstitium. However, the pore sizes of tumor vessels vary significantly; for instance, highly vascularized solid tumors (*e.g.*, breast cancer, liver cancer) can have vascular gaps ranging from 200–800 nm, while poorly vascularized or dense matrix tumors (*e.g.*, pancreatic cancer) may have pore sizes less than 100 nm, at which point the classic EPR effect is significantly attenuated.^143^ Active magnetic navigation can forcibly guide magnetic hydrogel-based micro/nano-robotic medical devices to the tumor area when the magnetic field strength gradient (∇B) is sufficiently high. However, magnetic field penetration depth and gradient attenuation are also influenced by tissue type; deep-seated organ tumors require higher field strengths or implantable field sources to maintain an adequate gradient. In tumor models with poor vascular permeability or insignificant EPR effect, the contribution of active magnetic navigation significantly increases. In highly permeable, superficial tumors, both effects often exhibit a superposition, allowing simultaneous utilization of passive EPR accumulation and active magnetic enrichment for spatiotemporally synergistic delivery. Therefore, for different tumor types, driving parameters and administration routes should be optimized by combining imaging results with vascular permeability assessments, drawing upon the experience of currently clinically approved nanomedicines or advanced drug delivery systems to enhance targeting efficiency and clinical translation success rates.^7,18,143^ This aligns with the importance of refined patient stratification and dosing strategies in cancer nanodrug clinical trials, as proposed in 18.Furthermore, to optimize the therapeutic window for tumor treatment, an in-depth investigation into the dose-exposure-effect relationship is imperative. This not only involves assessing the actual exposure level of a given dose of hydrogel-based micro/nano-robotic medical devices in tumor tissue, such as the intratumoral accumulation or drug concentration (AUC tumor) measured by imaging techniques, but also quantifying the dose-dependency between this exposure level and the ultimate therapeutic effect, for instance, tumor growth inhibition rate or apoptosis rate. For example, research by Jiang *et al.* and Tao *et al.*, using magnetically driven hydrogel-based micro/nano-robotic medical devices for anticancer drug delivery, has begun to explore the relationship between drug concentration and tumor inhibition.^7,18^ However, future research needs to more systematically provide specific PK parameters for hydrogel-based micro/nano-robotic medical devices in tumor and normal tissues at different administration doses, such as *C*_max_, tumor *vs. C*_max_, normal_tissue, and the ratio of AUC tumor to AUC normal_tissue. This will enable precise assessment of the local therapeutic index and guide preclinical studies to determine optimal dosing regimens. This is crucial for advancing hydrogel-based micro/nano-robotic medical devices to the clinical trial stage in the future, and requires strict adherence to relevant regulatory science guidelines to improve the success rate of clinical trials.^59^

As shown in Table 4, the matrix integrates the main benefits and potential risks of hydrogel-based micro/nano-robotic medical devices in targeted drug delivery, providing a decision-making reference for optimizing material design and actuation strategies. Overall, this matrix offers an intuitive framework for comprehensive design aimed at maximizing benefits and minimizing risks across pharmacokinetics (PK), pharmacodynamics (PD), and safety.

**Table 4:** Risk-benefit matrix of hydrogel-based micro/nano-robotic medical devices

	High yield	Medium income	Low returns
High risk	Invasive magnetically controlled hydrogel-based micro/nano-robotic medical devices for targeted brain tumor therapy (offering high precision and high therapeutic efficacy, but carrying risks such as hemorrhage, deep tissue damage, long-term retention of functional nanoparticles, and potential toxicity from degradation products).^5,7,18,21,53,54,58^	High-power photothermal effect hydrogel-based micro/nano-robotic medical devices for local therapy (effective, enabling precise local ablation, but carrying risks such as non-specific thermal damage, limited tissue penetration depth, and long-term retention of functional nanoparticles).^9,11,53,58^	Application of hydrogel-based micro/nano-robotic medical devices containing highly toxic residues (*e.g.*, unreacted monomers, crosslinkers) or difficult-to-degrade functional components (high cytotoxicity, accumulated immunogenicity risk, and relatively limited functional value).^53,54,58^
Moderate risk	Magnetically controlled hydrogel-based micro/nano-robotic medical devices for targeted tumor delivery (offering efficient drug enrichment, reduced systemic toxicity, and cell-level targeting, but still facing challenges such as magnetic field attenuation in deep tissues, complex PK/PD characteristics, and immunogenicity).^7,11–13,18,60,107^	Multi-actuation synergistic hydrogel-based micro/nano-robotic medical devices for localized drug release (precise, controllable, enhanced environmental adaptability, moderate risk, but may still be subject to physiological interference or require precise regulation of synergistic mechanisms).^8,11^	Single physiological response (*e.g.*, pH, temperature) hydrogel-based micro/nano-robotic medical devices for drug delivery (susceptible to physiological buffer systems or environmental interference, leading to premature drug release or limited release precision and efficiency, with functional stability needing improvement).^8,10,98,123^
Low risk	Natural polysaccharide or DNA hydrogel-based micro/nano-robotic medical devices for biosensing (high biosafety, high biocompatibility, used for early diagnosis of cancer biomarkers, pathogens, *etc.*, offering high sensitivity and programmability).^4,24,25,30–34,56^	Non-invasive low-intensity magnetic field-controlled hydrogel-based micro/nano-robotic medical devices for assisted diagnosis/simple operations (remotely controllable, high biosafety, but magnetic field attenuation may limit high-precision operations in deep tissues).^7^	While traditional biocompatible hydrogels used for conventional sustained release offer high safety and good biocompatibility, they are generally limited by a lack of intelligent responsiveness and precise control, alongside their singular functionality.^14,15,56^

### 4.2. Minimally invasive surgery and diagnostics

Hydrogel-based micro/nano-robotic medical devices can be actuated *via* external mechanisms such as magnetic fields, electric fields, or optical fields, enabling precise manipulation and motion within living organisms. Their extremely small size allows them to freely navigate through narrow spaces such as blood vessels and interstitial tissue gaps, thereby enabling targeted interventions at pathological sites. This approach significantly reduces damage to healthy tissue during surgical procedures, enhancing both safety and precision. Compared to conventional open surgery, such systems offer reduced invasiveness, high precision in treatment, multifunctionality, accelerated recovery, and lower incidence of postoperative complications. For instance, Piantanida *et al.* developed injectable nanocomposite hydrogels for minimally invasive procedures involving tissue repair, cancer therapy, and electroactive tissue engineering. Through chemical crosslinking or physical entanglement of polymer chains, along with interfacial interactions between conductive polymers, carbon nanotubes, and the polymer matrix, mechanical strength, electrical conductivity, self-healing capacity, and antibacterial functionality are imparted to hydrogels. These advanced hydrogels have been applied in tumor therapy, cartilage regeneration, and electroactive tissue repair.^5^ Compared to micro/nano-robotic medical devices constructed from rigid materials—which, in complex surgical contexts, may easily damage tissue, exhibit low freedom of movement, and have poor biocompatibility—hydrogels based on flexible materials simplify mechanical architecture, adapt to physiological environments, and improve safety and practical utility in medical operations.^145^ Furthermore, with regard to biocompatibility, Laurano *et al.* enhanced the *in vivo* compatibility of hydrogel-based micro/nano-robotic medical devices by developing an injectable bio-artificial hydrogel *via* green chemistry, combining synthetic polyether urethane with natural hyaluronic acid. Functionalization through thiol and catechol groups enables *in situ* crosslinking by multiple routes: oxidative coupling of thiols to form disulfide bonds, Michael addition between thiols and alkenes, and covalent additions between oxidized catechol-derived quinones and amino or thiol groups. In cardiovascular therapy, such hydrogel-based micro/nano-robotic medical devices can be administered *via* intravascular injection to the affected sites, performing tasks such as thrombus removal and vascular repair with high fidelity. As illustrated in Fig. 16(B), dopamine oxidation influences the interactions of proteins, cells, and tissues with hydrogels. In neurosurgical applications, these devices can enter the brain through minimally invasive surgical incisions to carry out tumor resection and nerve repair, while integrating the advantages of synthetic and natural materials.^173^ In parallel, Chen *et al.* designed a three-layer magnetic soft robotic structure for targeted gastrointestinal drug delivery and ulcer treatment. Each layer contains a magnetic substrate and adhesive membrane: the lateral layers employ magnetic frameworks with non-magnetic substrates, and the central layer combines a non-magnetic framework with a magnetic substrate, optimizing interlayer magnetic attraction and separation.^32^ Consequently, hydrogels represent a critical material platform for micro/nano-robotic medical devices in minimally invasive surgery. Their unique properties enable specialized medical applications, including targeted drug delivery and biosensor integration for biomarker detection. Moreover, the intrinsic biocompatibility and biodegradability of hydrogel materials ensure safety within the body.^174,175^ Nevertheless, improving the chemical stability and reliability of hydrogel-based micro/nano-robotic medical devices remains a significant challenge, especially in achieving precise controllability of motion in complex biological environments. Continuous development and refinement of these systems are expected to markedly enhance surgical precision and reduce patient recovery time, signaling innovative progress in minimally invasive medical practice.

### 4.3. Biosensing

Among the diverse applications of hydrogel-based micro/nano-robotic medical devices, biosensing has remained a focal point of sustained interest due to its close association with early-stage disease detection, precise physiological monitoring, and intelligent responsive therapy. Hydrogel networks, characterized by high water content, excellent biocompatibility, and tunable stimulus-responsiveness, can serve both as flexible substrates that enhance mechanical compliance and as functionalized materials capable of transducing chemical, electrical, or optical signals. These properties enable the integration of target recognition, signal acquisition, and feedback response into a single operational framework for both *in vivo* and *ex vivo* applications.^4,165^

Early research primarily focused on real-time monitoring capabilities of fluorescent hydrogels, wherein dynamic fluorescence signal changes occur upon interaction between incorporated fluorophores and target analytes, allowing detection of disease biomarkers with molecular-level sensitivity for early diagnosis.^20^ For example, Bai *et al.* employed molecular regulation of aggregation-induced emission (AIE) materials to enhance fluorescence properties and optimize the molecular recognition-based responsive sensing performance of DNA hydrogels, achieving rapid detection of cancer biomarkers and pathogens, and demonstrating the advantages of hydrogels in specific biomolecular recognition.^32–34^ Such methods often rely on conformational changes or the formation/cleavage of chemical bonds within the hydrogel network, which in turn affect fluorophore quantum yield or excitation/emission wavelengths. Li *et al.* developed DNA hydrogels and microgels that utilize complementary DNA strand pairing for specific target detection. *In vitro*, changes in gel volume or optical properties as a function of analyte concentration are employed; sol–gel transitions trigger the release of signaling molecules to amplify detection output. *In vivo*, injectable DNA microgels enable real-time monitoring of localized biomarkers *via* fluorescence imaging.^4^ Similarly, Singh *et al.* fabricated a dual-mode microfluidic biosensor for quantitative detection of cardiac myoglobin in human serum. This device utilized mesoporous cysteine–graphene oxide hydrogels wherein thiol and amino groups of cysteine chemically modify the functional groups on the graphene surface. By leveraging the high surface reactivity and exceptional conductivity of graphene, detection sensitivity was enhanced, making this approach suitable for clinical serum sample analysis. The mesoporous structure of Cys–RGO hydrogels amplified surface plasmon resonance (SPR) signals, enabling real-time monitoring of molecular recognition and binding kinetics between cMb and the sensing interface, while providing label-free detection to validate electrochemical measurements.^176^ Sokolov *et al.* reported composite hydrogels containing inorganic fluorescent nanocrystals, with high fluorescence quantum yield, photostability, and spectrally tunable emission. The three-dimensional hydrogel structure enhanced immobilization capacity for biomolecules, improving target analyte capture efficiency. Coupled with micro/nano-robotic medical devices, detection could be achieved through analyte-induced fluorescence quenching or enhancement. Applications spanned detection of metal ions, bacteria, biomolecules, and disease-related biomarkers.^177^ Deng *et al.* demonstrated that surface functionalization of hydrogel-based micro/nano-robotic medical devices enables selective capture of specific biomarkers. Magnetic actuation was employed to enhance enrichment at target sites, thereby improving detection sensitivity. For example, magnetic devices carrying enzyme labels were used to catalyze chromogenic reactions for colorimetric detection of low-abundance biomolecules.^178^ Building upon this, Liu *et al.* developed a dual-mode microfluidic biosensor based on target-triggered DNA hydrogel degradation for simultaneous colorimetric and electrochemical analysis. Molecular recognition between organophosphates and aptamers embedded in the DNA hydrogel induced conformational changes, leading to dissociation of double-stranded or multi-stranded DNA structures, hydrogel network breakdown, and release of pre-loaded detection probes, thereby amplifying the detection signal and improving sensitivity.^179^ In summary, hydrogel-based micro/nano-robotic medical devices offer high sensitivity and selectivity in biosensing applications, with capabilities for real-time monitoring, dynamic feedback, and non-invasive detection. Beyond sensing and detection of substances within the body, these devices can achieve high-resolution imaging and visualization of pathological sites through integration with external modalities such as fluorescence microscopy, magnetic resonance imaging, or the use of imaging agents like fluorescent dyes and magnetic particles. Such imaging provides clinicians with intuitive and detailed information on lesion morphology, location, and size, thereby supporting accurate diagnosis and treatment planning.^6,177^ For instance, Han *et al.* developed biodegradable acoustic hydrogels capable of both propelling hydrogel-based micro/nano-robotic medical devices and enhancing ultrasound signal contrast *via* internal gas cavities, enabling real-time *in vivo* imaging. As shown in Fig. 16(C), micro-scale propulsion units (“micro-rockets”) were driven forward in vasculature by near-infrared continuous-wave lasers, while pulsed lasers were used for photoacoustic imaging. Therapeutic agents carried by the devices were precisely delivered to tumor regions through combined acoustic/magnetic actuation, with real-time feedback monitoring of drug release to ensure delivery efficiency.^6^ Laser-based methods are also viable for imaging: Li *et al.* demonstrated that gold-coated micro/nano-robotic medical devices can absorb laser energy to produce photoacoustic signals, which are detected *via* ultrasound sensors to differentiate devices from background in blood with high imaging contrast, enabling high-resolution tracking of individual propulsion units. In their work, near-infrared light served as the driving stimulus, in which the photothermal effect of the gold coating provided propulsion, while simultaneous laser excitation generated photoacoustic signals, achieving coordinated control of actuation and imaging.^163,180^

With the continuous advancements in materials science and micro/nano-robotic technologies, the application of hydrogels in biosensing has extended far beyond optical detection.^181^ Recent studies have demonstrated that by precisely modulating the interactions between polymer chains, it is possible to fabricate intelligent hydrogels that exhibit exceptional mechanical properties, high electrical conductivity, and intrinsic self-powering capabilities, thereby enabling broader and more complex sensing functions. In the domain of high-sensitivity mechanical sensing, Guo *et al.* reported a multifunctional conductive polymer–polyvinyl alcohol composite hydrogel (CP–PVA@PS hydrogel) in which inter- and intra-chain polymer interactions were finely manipulated through a combination of cyclic freeze–thaw processing and salt immersion treatment. This approach significantly improved the physical properties of the hydrogel, yielding elongation exceeding 180%, electrical conductivity above 20 S m^−1^, and an energy dissipation coefficient below 0.15, while achieving a modulus (∼200 kPa) matched to human skin. Such hydrogels were capable of high-frequency strain sensing, including detection of subtle vocal cord vibrations, thereby providing a new paradigm for non-invasive, real-time physiological monitoring. Furthermore, when employed as a flexible conductive layer, the hydrogel facilitated the construction of a self-powered tactile sensing system (STS). Based on the triboelectric nanogenerator principle, the STS converted human motion energy into electrical power while simultaneously realizing tactile sensing. This system exhibited ultrahigh sensitivity to both pressure and strain and was successfully applied in handwriting recognition, Morse code detection and decoding, and material identification, thereby presenting an innovative solution for wearable electronics and human–machine interface systems. These applications not only expand the dimensionality of hydrogel-based sensing but also open new avenues in intelligent prosthetics, rehabilitation training, and human–machine interaction.^182^ In the context of self-powering and environmental perception, Guo *et al.* developed a leaf-based energy harvester (LEH) by integrating the natural microstructural features of leaves with a super-hygroscopic iron hydrogel. The LEH efficiently harvested energy from ambient humidity by exploiting moisture gradients between wet and dry ends, coupled with “wet-contact ionic interactions” between the hydrogel and carbon black surfaces to form electrical double layers (EDLs). This configuration generated a sustained voltage output of up to 0.5 V over more than 200 hours, with remarkable self-recovery capacity. Although primarily designed for energy harvesting, this humidity-responsive mechanism—directly converting environmental humidity fluctuations into electrical signals—provides both conceptual and material foundations for the development of self-powered passive hydrogel-based humidity sensors or biological fluid moisture sensors. Such devices are particularly suited for long-term environmental monitoring or biofluid analysis without reliance on external power sources.^183^ Regarding bio-integration and on-demand powering, ultrasound-mediated triboelectric nanogenerators (U-TENGs) offer new possibilities for energy provision to hydrogel-based micro/nano-robotic medical devices and other bio-integrated sensors within the body. Guo *et al.* proposed an ultrasound-driven bio-adhesive triboelectric nanogenerator capable of immediate wound sealing and accelerated healing, wherein ultrasound served as the external stimulus to supply power to implanted sensors, activating them only when needed to perform specific functions. Although hydrogels were not directly employed as triboelectric materials in that particular study, the design concept of transient electronic devices—biodegradable and resorbable after task completion—holds significant implications for the integration of power systems into hydrogel-based devices while ensuring biosafety. Combining such on-demand powering mechanisms with hydrogel-based sensing could overcome limitations associated with conventional batteries, including size constraints and biocompatibility issues, thereby achieving safer, longer-lasting biosensing within the human body.^184^

In summary, the applications of hydrogel-based micro/nano-robotic medical devices and their intelligent hydrogel materials in the field of biosensing are advancing toward multifunctionality, self-powering capability, ultra-high sensitivity, high-level bio-integration, and transient operation. Through material innovations such as the design of composite hydrogels and the adoption of multi-actuation cooperative strategies, researchers have been able to endow hydrogels with stronger environmental adaptability, more precise controllability, and broader sensing functionalities. These developments are expected to play a pivotal role in early-stage disease diagnosis, precise therapeutic efficacy monitoring, wearable health management, and intelligent human–machine interaction, thereby accelerating the clinical translation of intelligent diagnostic and therapeutic platforms.

### 4.4. Tissue engineering and regenerative medicine

Hydrogel-based micro/nano-robotic medical devices with excellent biocompatibility and biodegradability are also widely utilized in the fields of tissue engineering and regenerative medicine. Hydrogels play a crucial role in tissue engineering as three-dimensional biomimetic scaffolds that emulate the extracellular matrix (ECM) environment. Owing to their porous 3D network structure and physicochemical properties similar to those of natural ECM, hydrogels provide a dynamic microenvironment for cellular adhesion, proliferation, and differentiation.^185^ For example, Sarker *et al.* developed a covalently crosslinked oxidized alginate–gelatin hydrogel composite incorporating bioactive glass, as illustrated in Fig. 16(D). The bioactive glass embedded within the hydrogel scaffold was fabricated *via* freeze-drying to yield a porous composite hydrogel with tunable stiffness and degradability, intended for bone tissue engineering applications.^164^ Similarly, Tondera *et al.* investigated the degradation behavior, tissue interactions, and systemic responses of two gelatin-based hydrogels with different crosslinking degrees after subcutaneous implantation in immunocompetent nude mice. Multimodal imaging combined with histological analysis revealed that neither hydrogel caused long-term fibrosis or immune rejection, and both hydrogel types exhibited excellent ECM-mimicking capability.^186^ Subsequently, Madhusudana Rao *et al.* proposed a novel polysaccharide-based magnetic hydrogel by incorporating Fe_3_O_4_ nanoparticles to enable magnetic control, and validated its multifunctional potential in tissue engineering.^187^ In medical applications, hydrogel-based micro/nano-robotic medical devices often serve a dual role in minimally invasive surgery and tissue engineering—for instance, releasing therapeutic agents after surgery to promote wound healing or accelerate tissue regeneration. Bertsch *et al.* reviewed design principles of self-healing injectable hydrogels, which combine shear-thinning and self-repairing properties, allowing delivery through fine-needle minimally invasive injection for tissue regeneration and 3D bioprinting. The self-healing capability typically relies on reversible dynamic covalent bonds or non-covalent interactions, effectively reducing surgical trauma, accommodating irregular-shaped tissue defects, enabling precise control of cell/drug delivery, and improving cell survival rates, thereby offering important reference strategies for tissue engineering and regenerative medicine.^188^ He *et al.* developed a novel magnetic nano-actuator–protein fiber-coated hydrogel dressing, wherein fibrinogen surfaces were functionalized with RGD peptides to enhance cellular adhesion and migration. Superparamagnetic nanoparticles generated localized heating under near-infrared (NIR) irradiation to kill bacteria and prevent infection. The system integrated cyclic guanosine monophosphate (cGMP) signaling to promote angiogenesis and induce hair follicle regeneration. The highly oriented protein fiber coating mimicked natural ECM, providing mechanical support and bio-signal guidance. Magnetic nano-actuators thus combined therapeutic and diagnostic functionalities, driving the advancement of intelligent wound dressings.^189^ Hydrogel-based micro/nano-robotic medical devices have also been applied in large-animal models such as dogs and pigs. For example, Dong *et al.* developed micro–nanofiber composite conduits for repairing long-gap peripheral nerve defects in large animals. In addition to *in vitro* studies demonstrating modulation of cellular behavior by the micro–nanofiber structure, *in vivo* implantation in Beagle dogs successfully repaired 30 mm sciatic nerve defects, confirming that micro–nanofiber architectures can achieve effective tissue repair and regeneration in large-animal models.^159^

### 4.5. Clinical translation potential of hydrogel-based micro/nano-robotic medical devices: pharmacokinetic, pharmacodynamic, and immunogenicity perspectives

**Fig. 17:**
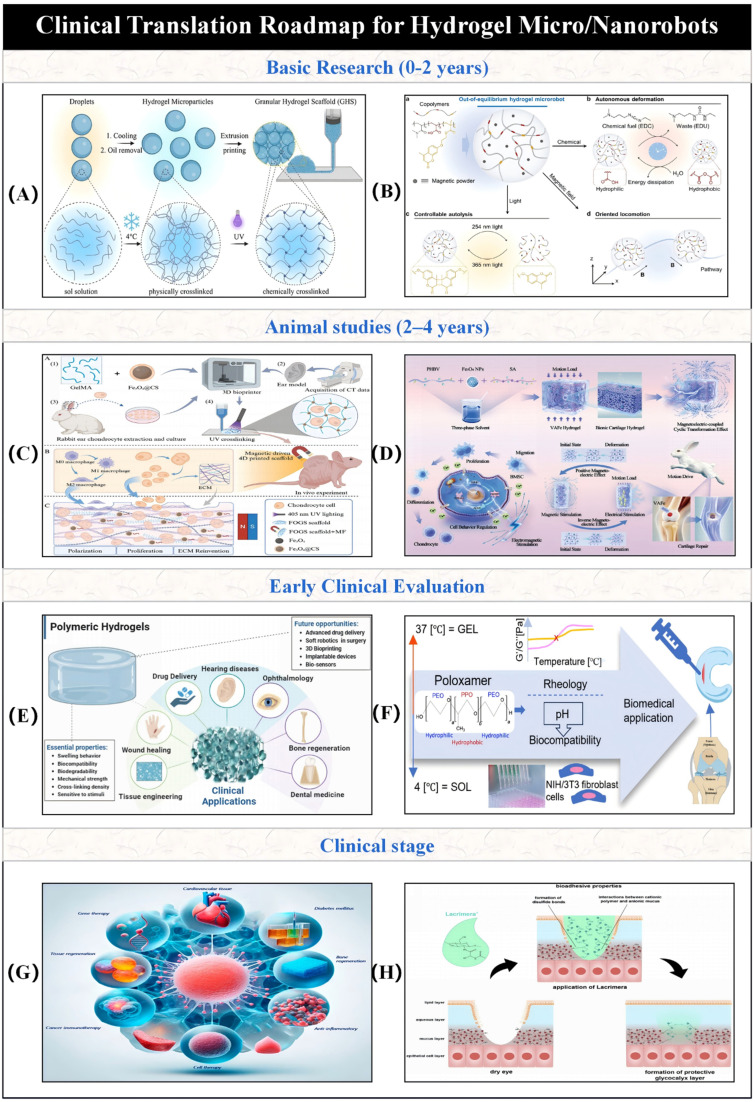
Clinical translation roadmap for hydrogel-based micro/nano-robotic medical devices. (A) Fabrication process of a porous particulate hydrogel scaffold. © 2025 Published by Elsevier Ltd.^190^ (B) Multifunctionally integrated non-equilibrium hydrogel-based micro/nano-robotic medical device. © 2025 Wiley-VCH GmbH.^191^ (C) *In vivo* experimentation in nude mice to investigate the process by which magnetic hydrogels induce chondrocyte proliferation and modulate anti-inflammatory immune responses. © 2025 Wiley-VCH GmbH.^192^ (D) *In vivo* experimentation in rabbits demonstrating efficient cartilage defect repair using VAFe biomimetic cartilage hydrogels. © 2025 Wiley-VCH GmbH.^193^ (E) Evaluation of key physicochemical properties of polymeric hydrogels, alongside a broad medical application landscape. © 2025 The Author(s). Published by Elsevier B.V.^194^ (F) Overall conceptual framework for thermosensitive poloxamer hydrogels—encompassing structure and temperature-responsive characteristics, rheological and biocompatibility assessment, and their potential injectable applications in bone and joint injury repair. © 2025 The Authors. Published by the American Chemical Society.^195^ (G) Hydrogel encapsulation to protect cells and drugs from adverse *in vivo* environments, enabling precise release and therapeutic applications across multiple domains, including regenerative medicine, immunotherapy, and chronic disease management. © 2024 The Authors. Published by the American Chemical Society.^196^ (H) Application of hydrogel-based products in the treatment of dry eye syndrome. © 2024 The Authors. Published by Elsevier Ltd.^197^

Although hydrogel-based micro/nano-robotic medical devices have demonstrated significant advantages in actuation modes, functional integration, and *in vitro* experimentation, their clinical translation still faces critical bottlenecks. The full-chain pathway from technological breakthroughs to clinical implementation—illustrated in Fig. 17—constitutes the essential process by which hydrogel-based micro/nano-robotic medical devices can be brought into practical medical use. This pathway encompasses sequential stages including fundamental research, animal studies, early human evaluations, and eventual clinical trials. In a truly clinical context, such devices must not only achieve precise motion control and stimulus-responsiveness but also undergo systematic evaluation of their pharmacological behavior and biocompatibility. From the perspectives of pharmacokinetics, pharmacodynamics, and immunogenicity, it is possible to dissect the key challenges and potential solutions for hydrogel-based micro/nano-robotic medical devices in the course of clinical translation.

The pharmacokinetic (PK) characteristics of hydrogel-based micro/nano-robotic medical devices are closely related to their material composition, structural design, and actuation mode. In terms of spatiotemporal distribution and targeting efficiency, the *in vivo* biodistribution of such devices is not only influenced by physiological factors such as hemodynamics and vascular permeability, but also by their autonomous locomotion capabilities. Although magnetically actuated devices can be navigated *via* external fields, their penetration efficiency, targeting precision, and retention time in complex biological environments—such as mucus layers and cellular matrices—still require quantitative assessment. A systematic evaluation should involve controlled dosing, acquisition of drug concentration–time profiles in both blood and target tissues, and calculation of key PK parameters such as area under the curve (AUC) and peak concentration (*C*_max_). Wang *et al.* addressed the issue of stability and spatial ordering in magnetic nanoparticle (MNP) cluster distribution, a prerequisite for precise spatiotemporal control. They utilized thermosensitive hydrogels as carriers to immobilize and encapsulate MNP clusters, thereby “freezing” specific assembly patterns within microspheres to form structurally stable devices. This ensured the preservation of their internal architecture against mechanical disruption in complex fluid environments, maintaining consistent and predictable physical functionality. By regulating gravitational fields, gradient magnetic fields, and uniform magnetic fields—along with tuning particle hydrophilicity/hydrophobicity and surface charge—they achieved precise programming of spatial arrangements in MNP clusters. Six distinct assembly patterns were generated, constituting a “device library” in which spatial arrangement determined subsequent motion and functional performance. Future research should establish multi-scale models to correlate actuation parameters with distribution dynamics, integrating PK data to enable accurate prediction of PK behavior and preliminary assessment of the therapeutic window.^11^ Regarding degradation and clearance mechanisms, the degradation profile of the hydrogel matrix directly affects the functional duration and *in vivo* retention risk of the device. Current studies primarily focus on *in vitro* degradation performance, whereas *in vivo* degradation kinetics, chemical composition and molecular weight distribution of degradation fragments, and clearance routes under physiological conditions (*e.g.*, enzymatic and pH-responsive processes) remain inadequately characterized. Qin *et al.* focused on constructing a magnetically actuated micro-motor system for active tumor DNA detection, validating its locomotion performance, detection capability, and biocompatibility. The fabricated micro-motor was sized to allow movement within large blood vessels; however, this dimension greatly exceeded the clearance threshold of the human body. Millimeter-scale objects, once implanted or injected, are nearly impossible to eliminate *via* normal physiological pathways, posing a substantial risk of long-term retention that could trigger foreign body responses, fibrotic encapsulation, or other complications. To address this issue, the motor body was fabricated from polyvinyl alcohol (PVA), a material widely used in drug delivery and tissue engineering. *In vivo* degradation of PVA primarily occurs through hydrolysis and enzymatic processes, producing metabolites that can be naturally processed by the body—representing a positive signal for degradability potential. Cytotoxicity and hemolysis tests confirmed that material extracts exhibited good biocompatibility at tested concentrations.^19^ Special consideration must be given to renal clearance thresholds and the capture effect of the reticuloendothelial system (RES), with systematic evaluation of metabolic and excretory pathways of the device. In terms of drug release kinetics, hydrogels serving as drug carriers are subject to multi-factorial regulation, including material swelling, degradation, and external stimuli. Ideally, therapeutic payloads should be released in a controlled manner within the target site rather than prematurely leaking into circulation. A key challenge at present lies in establishing quantitative relationships between actuation parameters and drug release rates, while assessing environmental interference on release kinetics. This is critical for precise control of drug exposure in the target site, forming the basis for evaluating the therapeutic window and achieving optimal therapeutic efficacy.

Efficient targeted enrichment does not necessarily equate to therapeutic gain; therefore, a robust pharmacodynamic (PD) evaluation framework for hydrogel-based micro/nano-robotic medical devices must be established. In the context of dose–effect relationships, unlike conventional drugs, the “dose” of such devices should encompass multiple dimensions—including device quantity, drug payload, and actuation energy. A multivariate model is required to elucidate the quantitative relationship between device number in the target site, drug release rate, and therapeutic outcomes such as tumor inhibition rate. For instance, Jiang *et al.* demonstrated *in vitro* that lycorine hydrochloride exhibits a significant dose–response inhibitory effect on colorectal cancer cells, and further showed that a magnetically actuated hydrogel-based micro/nano-robotic drug delivery system markedly enhances its pharmacological potency, thereby providing preliminary data for establishing the dose–response curve.^7^ Regarding therapeutic index and toxicity profiling, compared with traditional drug delivery systems, device-based therapy should substantially improve the therapeutic index—enhancing target-site efficacy while reducing toxic side effects from systemic exposure. This requires precise determination of systemic exposure levels (AUC systemic) at various administration doses, along with biodistribution and clearance data for major organs, in order to quantify whole-body toxicity. Such datasets are indispensable for defining the therapeutic window. Current research must address this need by employing relevant disease models to compare the efficacy and toxicity of device-mediated *versus* conventional administration under equivalent drug dosing, for example, a standardized mg per kg dosage, with special emphasis on non-target organ distribution and potential tissue damage. In the context of combination therapeutic effects, hydrogel-based micro/nano-robotic medical devices can integrate multiple treatment modalities, including hyperthermia, chemotherapy, and gene therapy. PD studies should clarify the synergetic mechanisms and spatiotemporal optimization strategies between these modalities—for example, sequencing control of magnetothermal effects and drug release—such as magnetothermal-induced cleavage of chemical bonds within the hydrogel and the respective contribution of such events to overall therapeutic efficacy.

Even when hydrogel materials themselves exhibit excellent biocompatibility, their incorporation into a device as an exogenous entity can still trigger immune responses. The chemical properties, topological features, and protein adsorption profile of the device surface may activate the complement system or induce macrophage phagocytosis. In addition, degradation products and their functional components may act as antigens, potentially eliciting adaptive immune responses. A comprehensive assessment of immunoactivation risks should be conducted through *in vitro* co-culture with immune cells and *in vivo* animal studies. Strategies to reduce immunogenicity include biomimetic cell membrane coatings and functionalization with immunomodulatory molecules, aimed at actively suppressing phagocytosis and immune recognition.^198,199^ Careful consideration must be given to the compatibility of such surface modification strategies with device motility and payload delivery functions. Beyond acute immune responses, potential induction of chronic inflammation or fibrotic reactions during prolonged *in vivo* retention must be evaluated—particularly in scenarios involving repeated administrations over long durations. Balancing biodegradability with immunological safety thus becomes a critical determinant for the clinical translation of hydrogel-based micro/nano-robotic medical devices.

## 5. Conclusion and outlook

This article provides a comprehensive and in-depth review of hydrogel-based micro/nano-robotic medical devices, covering material selection, fabrication technologies, actuation mechanisms, and medical applications. Leveraging their outstanding biocompatibility, tunable physicochemical properties, and diverse stimulus-responsiveness, hydrogels have emerged as an ideal matrix for constructing micro/nano-robotic medical devices. From natural hydrogels to synthetic systems, and further to functionalized composite hydrogels, continuous innovation in material design has endowed these devices with enhanced mechanical performance, improved biological adaptability, and expanded multifunctionality. Advances in fabrication technologies—such as 3D/4D printing, electrodeposition, and microfluidics—have greatly increased the structural complexity and functional integration of hydrogel-based devices, enabling precise construction from simple microspheres to intricate heterogeneous architectures. In terms of actuation, hydrogel-based micro/nano-robotic medical devices have evolved from relying on single-mode propulsion (*e.g.*, chemical fuels or external physical fields) to employing multi-mechanism cooperative strategies, such as magnetic–optical or magnetic–pH-responsive combinations. These synergistic approaches have significantly improved motion precision, targeting efficiency, and environmental adaptability in complex biological settings, allowing execution of increasingly delicate and sophisticated tasks. From a clinical perspective, hydrogel-based micro/nano-robotic medical devices demonstrate substantial potential in targeted drug delivery, minimally invasive surgical assistance, biosensing, and tissue engineering and regenerative medicine. They offer promising solutions to longstanding challenges in conventional medicine, such as achieving higher localized drug concentrations, reducing systemic toxicity, enabling early disease diagnosis, and enabling precision therapy.

Despite the considerable progress achieved in hydrogel-based micro/nano-robotic medical devices, significant limitations remain. The successful transformation of such devices from laboratory research to clinical application continues to be hindered by a series of core challenges and bottlenecks. Foremost among these is the requirement for systematic biosafety evaluation—an indispensable checkpoint—which must comprehensively and rigorously address the intrinsic toxicity of the hydrogel materials, the toxicological effects of degradation products, and the long-term *in vivo* accumulation risks of functional nanoparticles. Equally critical is the precise characterization of pharmacokinetic (PK) profiles and their correlation with pharmacodynamic (PD) outcomes, necessitating the establishment of robust quantitative assessment methodologies. Furthermore, mitigation of immunogenicity—particularly when introducing exogenous functional components—represents a key prerequisite for ensuring safety in long-term applications. (1) Hydrogel material properties – not all hydrogel matrices inherently exhibit high biocompatibility. To address this, future development should focus on new hydrogel bases and functional components with superior biocompatibility, precisely controllable degradation rates, reduced immunogenicity, and the ability to be efficiently and non-toxically cleared from the body. Strategies may include deep integration of natural polymers and biomimetic materials, or active evasion of immune recognition through surface modifications. Alternatively, devices may be endowed with responsiveness to external physical fields, allowing retrieval of hydrogel-based micro/nano-robotic medical devices after task completion *via* remote actuation, thus enhancing safety. (2) Fabrication process optimization – certain high-precision hydrogel-based micro/nano-robotic medical devices require complex fabrication steps that challenge scalability and commercial production. To overcome current limitations in resolution, cost-efficiency, and scale-up, optimization of advanced manufacturing techniques—such as laser-based 3D printing—should be pursued to simplify workflows and adapt them for batch production. (3) Actuation mechanisms – present actuation strategies primarily rely on either external driving or intrinsic stimulus-responsiveness, with each exhibiting inherent constraints. The rising need for multi-mechanism cooperative hydrogel-based micro/nano-robotic medical devices calls for structural innovations. Increasing crosslink density, optimizing hybrid ratios, and employing dual-network hydrogels—where individual networks carry distinct functionalities—can enhance compatibility between multiple actuation modalities and facilitate coordinated operation. (4) Medical applications and performance monitoring – beyond visualization capabilities—which are crucial—the fundamental challenge lies in establishing complete dose–exposure–effect relationships and defining therapeutic windows. This may be addressed by integrating multiple stimulus-response modalities into a single micro-scale system; for example, constructing feedback-regulated platforms that combine optical, acoustic, and magnetic fields to monitor and control *in vivo* behavior. Optimization of multimodal imaging strategies for real-time visualization should be coupled with quantitative bioanalytical techniques to track PK parameters—such as AUC, *C*_max_, tissue distribution, degradation, and clearance—and to correlate these with PD effects including therapeutic efficacy and adverse reactions, thereby generating a solid scientific foundation for clinical translation. (5) Clinical translation and regulatory science – close adherence to evolving regulatory science requirements for biomedical products worldwide should be embedded into research design from the outset. Establishment of a comprehensive, standardized preclinical evaluation framework—comprising well-validated animal models, precise PK/PD analyses, and multidimensional immunological assessments—will provide robust datasets to support early-phase human clinical trials.

In summary, the design methodologies for hydrogel-based micro/nano-robotic medical devices, along with their potential applications in biomedical research fields such as drug delivery and tissue engineering, are poised for further advancement. Interdisciplinary collaboration will be essential to accelerate progress in this domain. Future development of hydrogel-based micro/nano-robotic medical devices will require innovation in materials and breakthroughs in manufacturing technologies to create a broader range of stable and safe hydrogel matrices, while continuously refining and optimizing actuation strategies. Achieving efficient multi-mechanism cooperative control will be key to driving their evolution toward multifunctional integration and synergistic multi-modal actuation, thereby promoting rapid expansion of medical applications.

## Author contributions

Junteng Yao: investigation, conceptualization, writing – original manuscript. Peng Yu: literature organizing, categorizing, summarizing framing. Xue-Bo Chen: methodology, conceptualization, guidance, writing – review and editing.

## Conflicts of interest

We declare that we have no known competing financial interests or personal relationships that have appeared to influence the work reported in this paper.

## Data Availability

No primary research results, software or code have been included and no new data were generated or analysed as part of this review.

## References

[cit1] Cao Q., Chen W., Zhong Y., Ma X., Wang B. (2023). Biomedical Applications of Deformable Hydrogel Microrobots. Micromachines.

[cit2] Song W., Li L., Liu X., Zhu Y., Yu S., Wang H., Wang L. (2024). Hydrogel microrobots for biomedical applications. Front. Chem..

[cit3] Li J. (2018). *et al.*, Development of a magnetic microrobot for carrying and delivering targeted cells. Sci. Robot..

[cit4] Li F. Y., Lyu D. Y., Liu S., Guo W. W. (2020). DNA Hydrogels and Microgels for Biosensing and Biomedical Applications. Adv. Mater..

[cit5] Piantanida E., Alonci G., Bertucci A., De Cola L. (2019). Acc. Chem. Res..

[cit6] Han H. (2024). *et al.*, Imaging-guided bioresorbable acoustic hydrogel microrobots. Sci. Robot..

[cit7] Jiang F., Zheng Q., Zhao Q., Qi Z., Wu D., Li W., Wu X., Han C. (2024). Magnetic propelled hydrogel microrobots for actively enhancing the efficiency of lycorine hydrochloride to suppress colorectal cancer. Front. Bioeng. Biotechnol..

[cit8] Peng X., Peng Q., Wu M., Wang W., Gao Y., Liu X., Sun Y., Yang D., Peng Q., Wang T., Chen X.-Z., Liu J., Zhang H., Zeng H. (2023). ACS Appl. Mater. Interfaces.

[cit9] Du X., Zhai J., Li X., Zhang Y., Li N., Xie X. (2021). ACS Sens..

[cit10] Zhang Z., Wang R., Yuan M., Huang X., Ding C., Wu H., Wang S., Liu A. (2022). Magnetically driven pH-responsive composite hydrogel for controlled drug delivery. Funct. Mater. Lett..

[cit11] Wang B., Liu D., Liao Y., Huang Y., Ni M., Wang M., Ma Z., Wu Z., Lu Y. (2022). ACS Nano.

[cit12] Vanhaeren M., Gijsbers R., Van Dyck W., Huys I., Simoens S. (2025). Advanced therapy medicinal products are coming of age: A pipeline analysis of the clinical trial landscape. Drug Discovery Today.

[cit13] Lin X., Zhang X., Wang Y., Chen W., Zhu Z., Wang S. (2025). Hydrogels and hydrogel-based drug delivery systems for promoting refractory wound healing: Applications and prospects. Int. J. Biol. Macromol..

[cit14] Zhou H., Zhu Y., Yang B., Huo Y., Yin Y., Jiang X., Ji W. (2024). J. Mater. Chem. B.

[cit15] Lavrador P., Esteves M. R., Gaspar V. M., Mano J. F. (2021). Stimuli-Responsive Nanocomposite Hydrogels for Biomedical Applications. Adv. Funct. Mater..

[cit16] Jamil M. F., Pokharel M., Park K. (2022). Light-Controlled Microbots in Biomedical Application: A Review. Appl. Sci..

[cit17] Vanderpoorten O., Peter Q., Challa P. K. (2019). *et al.*, Scalable integration of nano-,and microfluidics with hybrid two-photon lithography. Microsyst. Nanoeng..

[cit18] Tao Y., Li L., Yang X., Yin S., Zhang Z., Wang H., Pu R., Wang Z., Zhang Q., Mu H., Wu C., He J., Yang L. (2024). Magnetic-driven hydrogel microrobots for promoting osteosarcoma chemo-therapy with synthetic lethality strategy. Front. Chem..

[cit19] Qin F., Wu J., Fu D., Feng Y., Gao C., Xie D., Fu S., Liu S., Wilson D. A., Peng F. (2022). Magnetically driven helical hydrogel micromotor for tumor DNA detection. Appl. Mater. Today.

[cit20] Wang X., Ou Y., Wang X., Yuan L., He N., Li Z., Luo F., Li J., Tan H. (2023). J. Mater. Chem. B.

[cit21] Salzlechner C., Haghighi T., Huebscher I., Walther A. R., Schell S., Gardner A., Undt G., daSilva R. M. P., Dreiss C. A., Fan K., Gentleman E. (2020). Adhesive Hydrogels for Maxillofacial Tissue Regeneration Using Minimally Invasive Procedures. Adv. Healthcare Mater..

[cit22] Singh R., Datta B. (2023). ACS Appl. Polym. Mater..

[cit23] Lee S. R., Ong C. Y. J., Wong J. Y., Ke Y., Lim J. Y. C., Dong Z., Long Y., Hu Y. (2024). ACS Appl. Mater. Interfaces.

[cit24] Yu Z., Li Q., He X., Wang X., Wen Y., Zeng L., Yu W., Hu P., Chen H. (2023). A multifunctional hydrogel based on nature polysaccharide fabricated by Schiff base reaction. Eur. Polym. J..

[cit25] Berglund L., Rakar J., Johan P., Junker E., Forsberg F., Oksman K. (2020). ACS Appl. Bio Mater..

[cit26] Cho S., Lee J. (2020). Hydrogels of polyacrylic acid crosslinked by atorvastatin. J. Ind. Eng. Chem..

[cit27] Gao L., Luo H., Wang Q., Hu G., Xiong Y. (2021). ACS Omega.

[cit28] Raţă D. M., Cadinoiu A. N., Vochita G., Gherghel D., Lakkaboyana S. K., Fuioagă C. P., Atanase L. I., Ichim D. L. (2025). Biocomposite Complex Hydrogels With Antimicrobial Activity Suitable for Wound Healing. J. Polym. Sci..

[cit29] Tang K., Wang Z., Liu Y., Yan C., Ge Y., Zhang H., Song Y., Li H., Li X., He M., Lin Z., Duan T. (2025). Double-network composite hydrogel for efficient surface decontamination of nuclides. Chem. Eng. Sci..

[cit30] Xu H., Che Y., Zhou R., Wang L., Huang J., Kong W., Liu C., Guo L., Tang Y., Wang X., Yang X., Wang E., Xu C. (2024). Research progress of natural polysaccharide-based and natural protein-based hydrogels for bacteria-infected wound healing. Chem. Eng. J..

[cit31] Li S., Chen L. (2025). DNA hydrogels for biomedical applications: Advances and prospects. Chem. Eng. J..

[cit32] Bai X., Wang C., Huang L., Zhang H., Zhang J., Cao Y., Wang L., Pang W., Zhou H., Gao Z. (2025). Molecular modulation of aggregation-induced luminescence for improving response sensing of DNA hydrogels. Biosens. Bioelectron..

[cit33] Ma Z., Tang S., Shen W., Lee H. K. (2024). Advanced applications of DNA hydrogels in fluorescence sensing. Trac. Trends Anal. Chem..

[cit34] Xi L., Shang Y., Wang Z., Wang J., Wu Q., Shen Y., Ding Y. (2024). Programmable DNA hydrogels for biosensing and point-of-care test. Coord. Chem. Rev..

[cit35] Saito J., Furukawa H., Kurokawa T., Kuwabara R., Kuroda S., Hu J., Tanaka Y., Gong J. P., Kitamura N., Yasuda K. (2011). Polym. Chem..

[cit36] Xu R., Dou B., Yu S., Wang Z., Zhang Y., Leng L., Ouyang L., Sun W. (2025). Enabling 3D printability and vascular morphogenesis with double network dynamic hydrogels. Mater. Today.

[cit37] Londhe P. V., Londhe M. V., Salunkhe A. B., Laha S. S., Thompson Mefford O., Thorat N. D., Khot V. M. (2025). Magnetic hydrogel (MagGel): An evolutionary pedestal for anticancer therapy. Coord. Chem. Rev..

[cit38] Xing L. , Wang J., Guo Z., Shen W., Jia Z., Jia S., Li L., Wang J., Wang L., Li J., Chen Y., Sun Y., Zhang M., Bai J., Wang L. and Li X., bioRxiv, 2023, preprint, 10.19.563054, 10.1101/2023.10.19.563054

[cit39] Wen X., Wei J., Huang J., Kuo C.-F., Mei X., Xu S., Lu N. (2025). Injectable Arctium lappa polysaccharide-based composite hydrogel enhances diabetic wound healing. Int. J. Biol. Macromol..

[cit40] Jiang Y., Chen Y., Feng W., Zhong X., Yu D., Wang W. (2024). Rapid gelation of polyacrylic acids-like hydrogel via the dopamine acrylamide-Fe3+ system and its formation mechanism. Chem. Eng. J..

[cit41] Shen C., Li Y., Meng Q. (2022). Adhesive polyethylene glycol-based hydrogel patch for tissue repair. Colloids Surf., B.

[cit42] Gao F., Ma X., Wang F., Zhou F., Ye J., Yang D., Li M., Wang P. (2023). Injectable multifunctional DNA hydrogel for accelerated wound healing. Chem. Eng. J..

[cit43] Khajouei S., Ravan H., Ali E. (2020). DNA hydrogel-empowered biosensing. Adv. Colloid Interface Sci..

[cit44] Jia W., Liang Y., Wei X., Liang H., Chen X.-L. (2023). Chitosan-based double network hydrogel loading herbal small molecule for accelerating wound healing. Int. J. Biol. Macromol..

[cit45] Jiang Y., Wang J., Zhang H., Chen G., Zhao Y. (2022). Bio-inspired natural platelet hydrogels for wound healing. Sci. Bull..

[cit46] Liu W., Zhang X., Wei G., Su Z. (2018). Reduced Graphene Oxide-Based Double Network Polymeric Hydrogels for Pressure and Temperature Sensing. Sensors.

[cit47] Strachota B., Strachota A., Vratović L., Pavlova E., Šlouf M., Kamel S., Cimrová V. (2023). Exceptionally Fast Temperature-ResponsiveMechanically Strong and Extensibl Monolithic Non-Porous Hydrogels:Poly(N-isopropylacrylamide)Intercalated with Hydroxypropyl Methylcellulose. Gels.

[cit48] Santhosh M., Choi J.-H., Choi J.-W. (2019). Magnetic-Assisted Cell Alignment within a Magnetic Nanoparticle-Decorated Reduced Graphene Oxide/Collagen 3D Nanocomposite Hydrogel. Nanomaterials.

[cit49] Hu X., Nian G., Liang X., Wu L., Yin T., Lu H., Qu S., Yang W. (2019). ACS Appl. Mater. Interfaces.

[cit50] van Sprang J. F., Smits I. P. M., Nooten J. C. H., Fransen P. K. H., Söntjens S. H. M., van Houtem M. H. C. J., Janssen H. M., Rutten M. G. T. A., Schotman M. J. G., Dankers P. Y. W. (2025). J. Mater. Chem. B.

[cit51] Cao Q., Zhang Y., Tang Y. (2024). *et al.*, MOF-based magnetic microrobot swarms for pH-responsive targeted drug delivery. Sci. China Chem..

[cit52] Chen W., Sheng S., Tan K., Wang S., Wu X., Yang J., Hu Y., Cao L., Xu K., Zhou F., Su J., Zhang Q., Yang L. (2024). Mater. Horiz..

[cit53] Hushka E. A. , Blatchley M. R., Macdougall L. J., Max Yavitt F., Kirkpatrick B. E., Bera K., Dempsey P. J. and Anseth K. S., bioRxiv, 2024, preprint, 07.06.602364, 10.1101/2024.07.06.602364

[cit54] Cong L., Liu W., Tian L. (2025). *et al.*, A microgel composite hydrogel based on glucose response for drug delivery. Polym. Eng. Sci..

[cit55] Antoniou M., Melagraki G., Lynch I., Afantitis A. (2024). In Vitro Toxicological Insights from the Biomedical Applications of Iron Carbide Nanoparticles in Tumor Theranostics: A Systematic Review and Meta-Analysis. Nanomaterials.

[cit56] Zahedi Tehrani T., Irani S., Ardeshirylajimi A., Seyedjafari E. (2024). Natural based hydrogels promote chondrogenic differentiation of human mesenchymal stem cells. Front. Bioeng. Biotechnol..

[cit57] Jia J., Wu G., Zhang H., Wang F., Gu X., Dorma D., Zhang L., Chen H., Xu Y., Xie H. (2025). ACS Appl. Mater. Interfaces.

[cit58] Wang Z., Wang C., Ji Y., Yang M., Chan L., Li M., Yang J., Tang H., Luo X., Haoyang H., Liu Z., Chen K., Chang Y., Yuan H., Lin F., Xing G., Li J. (2025). Magnetically driven bionic nanorobots enhance chemotherapeutic efficacy and the tumor immune response via precise targeting. Innovation.

[cit59] Wu Z., Wang X., Lu Y., Xu Q. (2025). Multichamber magnetic capsule robot for selective liquid sampling and drug delivery. Natl. Sci. Rev..

[cit60] Xu Z., Wu Z., Yuan M., Chen Y., Ge W., Xu Q. (2023). Versatile magnetic hydrogel soft capsule microrobots for targeted delivery. iScience.

[cit61] Fu H., Yu B. (2024). 3D micro/nano hydrogel structures fabricated by two-photon polymerization for biomedical applications. Front. Bioeng. Biotechnol..

[cit62] Mazeeva A., Masaylo D., Razumov N., Konov G., Popovich A. (2023). 3DPrinting Technologies for Fabrication of Magnetic Materials Based on Metal–Polymer Composites: AReview. Materials.

[cit63] Billiet T., Vandenhaute M., Schelfhout J., Van Vlierberghe S., Dubruel P. (2012). A review of trends and limitations in hydrogel-rapid prototyping for tissue engineering. Biomaterials.

[cit64] Fu H., Yu B. (2024). 3D micro/nano hydrogel structures fabricated by two-photon polymerization for biomedical applications. Front. Bioeng. Biotechnol..

[cit65] Lee Y.-W., Kim J.-K., Bozuyuk U., Dogan N. O., Khan M. T. A., Shiva A., Wild A.-M., Sitti M. (2023). Multifunctional 3D-Printed Pollen Grain-Inspired Hydrogel Microrobots for On-Demand Anchoring and Cargo Delivery. Adv. Mater..

[cit66] Sanchis-Gual R., Ye H., Ueno T., Landers F. C., Hertle L., Deng S., Veciana A., Xia Y., Franco C., Choi H., Puigmartí-Luis J., Nelson B. J., Chen X.-Z., Pané S. (2023). 3D Printed Template-Assisted Casting of Biocompatible Polyvinyl Alcohol-Based Soft Microswimmers with Tunable Stability. Adv. Funct. Mater..

[cit67] Li J., Cao J., Bian R. (2025). *et al.*, Multimaterial cryogenic printing of three-dimensional soft hydrogel machines. Nat. Commun..

[cit68] Chen Y., Li M., Tang Q., Cheng Y., Miao A., Cheng L., Zhu S., Luo T., Liu G., Zhang L., Niu F., Zhao L., Chen J., Yang R. (2023). High-Speed NIR-Driven Untethered 3D-Printed Hydrogel Microrobots in High-Viscosity Liquids. Adv. Intell. Syst..

[cit69] Lee Y.-W., Ceylan H., Ceren Yasa I., Kilic U., Sitti M. (2021). ACS Appl. Mater. Interfaces.

[cit70] Deng C., Liu Y., Fan X., Jiao B., Zhang Z., Zhang M., Chen F., Gao H., Deng L., Xiong W. (2023). Femtosecond Laser 4D Printing of Light-Driven Intelligent Micromachines. Adv. Funct. Mater..

[cit71] Yue C., Li B., Wang J., Wang Y., Wang L., Wei M., Wang Y., Chen Z., Zhao G. (2025). Langmuir.

[cit72] Sadraei A., Naghib S. M. (2024). 4D Printing of Physical Stimuli-Responsive Hydrogels for Localized Drug Delivery and Tissue Engineering. Polym. Rev..

[cit73] Young Cho S. (2024). et al. Int. J. Extrem. Manuf..

[cit74] Wang Y., Zhu H., Ye X., Ge Y., Jie Z., Zhao Y., Jiang C. (2025). 4D Printing of magneto/thermo-responsive, adaptive and multimodal soft robots. Virtual Phys. Prototyp..

[cit75] Zheng Z., Han J., Demir S. O., Wang H., Jiang W., Liu H., Sitti M. (2023). Electrodeposited Superhydrophilic-Superhydrophobic Composites for Untethered Multi-Stimuli-Responsive Soft Millirobots. Adv. Sci..

[cit76] Zheng Z. (2022). *et al.*, Programmable aniso-electrodeposited modular hydrogel microrobots. Sci. Adv..

[cit77] Zhong S., Xin Z., Hou Y., Li Y., Huang H. W., Sun T., Shi Q., Wang H. (2024). Double-Modal Locomotion of a Hydrogel Ultra-Soft Magnetic Miniature Robot with Switchable Forms. Cyborg Bionic Syst..

[cit78] Zhu P., Hosneolfat Z., Nekoonam N., Bhagwat S., Helmer D., Rapp B. E. (2025). Fabricating Microstructures on Freeform Surfaces via Flexible Hydrogel Micromolds. Small.

[cit79] Xu Z., Ma J., Hu H., Liu J., Yang H., Chen J., Xu H., Wang X., Luo H., Chen G. (2025). Metal ioncrosslinking multifunctional hydrogel microspheres with inflammatory immune regulation for cartilage regeneration. Front. Bioeng. Biotechnol..

[cit80] Zhao D., Qian L., Yang Q., Xiang L., Ye C., Shi T., Wang Y. (2025). Microfluidic synthesis of stimuli-responsive hydrogel particles. Appl. Mater. Today.

[cit81] Wang P., Yang Y., Yin Y., Zhang H., Shi T., Zhang W., Liao H., Li S., Tan X., Yao Z., Chi B. (2025). ACS Appl. Polym. Mater..

[cit82] Muir V. G., Qazi T. H., Shan J., Groll J., Burdick J. A. (2021). ACS Biomater. Sci. Eng..

[cit83] Henise J., Yao B., Ashley G. W., Santi D. V. (2021). Facile preparation of tetra-polyethylene glycol hydrogel microspheres for drug delivery by cross-flow membrane emulsification. Eng. Rep..

[cit84] Chi H., Qiu Y., Ye X., Shi J., Li Z. (2023). Preparation strategy of hydrogel microsphere and its application in skin repair. Front. Bioeng. Biotechnol..

[cit85] Ying Teo M., Kee S., RaviChandran N., Stuart L., Kean C., Stringer J. (2020). ACS Appl. Mater. Interfaces.

[cit86] Ozbolat I. T., Hospodiuk M. (2016). Current advances and future perspectives in extrusion-based bioprinting. Biomaterials.

[cit87] Feng Z., He D., Zhang L., Li Q., Xue C., Yi X., Liao L., Pei Z., Shen X. (2025). Preparation of myofibrillar protein oleogels by emulsion template method: Application of fat substitute for sponge cakes. LWT–Food Sci. Technol..

[cit88] Huo H., Ye B., Shi Y., Feng C., Wang J., Li M., Fan J., Li L., Wang J., An C. (2023). Preparation of HNS microspheres by rapid membrane emulsification. Particuology.

[cit89] Zhou X., Hou C., Chang T.-L., Zhang Q., Liang J. F. (2020). Controlled released of drug from doubled-walled PVA hydrogel/PCL microspheres prepared by single needle electrospraying method. Colloids Surf., B.

[cit90] Shao L. (2022). et al. Biofabrication.

[cit91] Ciarleglio G., Russo T., Toto E., Santonicola M. G. (2024). Fabrication of Alginate/Ozoile Gel Microspheres by Electrospray Process. Gels.

[cit92] Mostofizadeh M., Kainz M., Alihosseini F., Haudum S., Youssefi M., Bauer P., Gnatiuk I., Brüggemann O., Zembsch K., Rinner U., Coelho C., Guillén E., Teasdale I. (2024). ACS Appl. Mater. Interfaces.

[cit93] Li X., Liu B., Pei B., Chen J., Zhou D., Peng J., Zhang X., Jia W., Xu T. (2020). Chem. Rev..

[cit94] Thareja P., Swarupa S., Ahmad S., Esther Jinugu M. (2025). Hydrogel-based inks for extrusion 3D printing: A rheological viewpoint. Curr. Opin. Colloid Interface Sci..

[cit95] Chen N., Zhang X., Lyu J., Zhao G., Gu K., Xia J., Chen Z., Shao Z. (2022). Soft Matter.

[cit96] Ye Y., Wan Z., Gunawardane P., Hua Q., Wang S., Zhu J., Chiao M., Renneckar S., Rojas O. J., Jiang F. (2024). Ultra-Stretchable and Environmentally Resilient Hydrogels Via Sugaring-Out Strategy for Soft Robotics Sensing. Adv. Funct. Mater..

[cit97] Wang H., Gu X., Wang C. (2016). ACS Appl. Mater. Interfaces.

[cit98] Xu H., Bai S., Gu G., Gao Y., Sun X., Guo X., Xuan F., Wang Y. (2022). ACS Appl. Mater. Interfaces.

[cit99] Wang H., Kan J., Zhang X., Gu C., Yang Z. (2021). Pt/CNT Micro-Nanorobots Driven by Glucose Catalytic Decomposition. Cyborg Bionic Syst..

[cit100] Yeruva T., Morris III R. J., Kumar S., Zhao L., Kofinas P., Duncan G. A. (2025). Biomater. Sci..

[cit101] Duan Q., Gao J., Qi Z., Wang X., Li H., Guo X., Han D., Wang X., Xi Y., Guo L., Li P., Xue J., Sang S. (2024). Photothermal effects of supra-CNDs@GelMA composite hydrogels under near-infrared stimulation. Colloids Surf., A.

[cit102] Zhang J., Liu J., He S., Cui Z., Shao W. (2023). Preparation and characterization of thermal/NIR/magnetically actuated hydrogel based on asymmetric structure. Colloids Surf., A.

[cit103] Ijaz F., Tahir H. M., Ali S., Ali A., Khan H. A., Muzamil A., Manzoor H. H., Qayyum K. A. (2023). Biomolecules based hydrogels and their potential biomedical applications: A comprehensive review. Int. J. Biol. Macromol..

[cit104] Lai Y. P., Lee T., Sieben D., Gauthier L., Nam J., Diller E. (2024). Hybrid Hydrogel-Magnet Actuated Capsule for Automatic Gut Microbiome Sampling. IEEE Trans. Biomed. Eng..

[cit105] Fu Q., Zhang S., Guo S., Guo J. (2018). Performance Evaluation of a Magnetically Actuated Capsule Microrobotic System for Medical Applications. Micromachines.

[cit106] Hu W., Lum G., Mastrangeli M. (2018). *et al.*, Small-scale soft-bodied robot with multimodal locomotion. Nature.

[cit107] Chen B., Zhang S., Guo H., Yang M., Guan J., Mou F. (2025). Chem. Asian J..

[cit108] Shi W., Huang J., Fang R., Liu M. (2020). ACS Appl. Mater. Interfaces.

[cit109] Zhou H., Mayorga-Martinez C. C., Pané S., Zhang L., Pumera M. (2021). Chem. Rev..

[cit110] Xu K., Liu B. (2021). Recent progress in actuation technologies of micro/nanorobots. Beilstein J. Nanotechnol..

[cit111] Huang H., Feng Y., Yang X., Yang L., Shen Y. (2022). An Insect-Inspired Terrains-Adaptive Soft Millirobot with Multimodal Locomotion and Transportation Capability. Micromachines.

[cit112] Chen X., Tian C., Zhang H., Xie H. (2023). ACS Appl. Mater. Interfaces.

[cit113] Islam M. S., Molley T. G., Jalandhra G. K., Fang J., Kruzic J. J., Kilian K. A. (2025). Magnetoactive Nanotopography on Hydrogels for Stimulated Cell Adhesion and Differentiation. Small Sch..

[cit114] Qiao S., Ouyang H., Zheng X., Qi C., Ma L. (2023). J. Mater. Chem. B.

[cit115] Ye M., Zhou Y., Zhao H., Wang Z., Nelson B. J., Wang X. (2023). A Review of Soft Microrobots: Material, Fabrication, and Actuation. Adv. Intell. Syst..

[cit116] Okada S., Takashima Y., Nakamura H. (2025). ACS Appl. Polym. Mater..

[cit117] Xuan X., Li Y., Xu X., Pan Z., Li Y., Luo Y., Sun L. (2025). Three-Dimensional Printable Magnetic Hydrogels with Adjustable Stiffness and Adhesion for Magnetic Actuation and Magnetic Hyperthermia Applications. Gels.

[cit118] Quan K., Mao Z., Lu Y., Qin Y., Wang S., Yu C., Bi X., Tang H., Ren X., Chen D., Cheng Y., Wang Y., Zheng Y., Xia D. (2024). Mater. Horiz..

[cit119] Chen X., Tian C., Zhang H., Xie H. (2024). J. Mater. Chem. B.

[cit120] Zhu Q. L., Du C., Dai Y. (2020). *et al.*, Light-steered locomotion of muscle-like hydrogel by self-coordinated shape change and friction modulation. Nat. Commun..

[cit121] Cheng Q., Lu X., Tai Y., Luo T., Yang R. (2024). ACS Biomater. Sci. Eng..

[cit122] Nan M., Guo K., Jia T., Wang G., Liu S. (2024). ACS Appl. Mater. Interfaces.

[cit123] Zhao T., Tan Y., Li Y., Wang X. (2025). Ionic fuel-powered hydrogel actuators for soft robotics. J. Colloid Interface Sci..

[cit124] Jiang H., Tang J. (2020). ACS Appl. Polym. Mater..

[cit125] Lee M. J., Shrotriya D. R., Espinosa-Marzal R. M. (2024). Responsiveness of Charged Double Network Hydrogels to Ionic Environment. Adv. Funct. Mater..

[cit126] Gao Y., Wang X., Chen Y. (2024). RSC Adv..

[cit127] Ni C., Chen D., Wen X. (2023). *et al.*, High speed underwater hydrogel robots with programmable motions powered by light. Nat. Commun..

[cit128] Mustakim N., Pandey M., Seo S.-W. (2025). Surface photografted thermoresponsive hydrogel microvalves on PDMS/silicon hybrid membrane for light-actuated localized chemical release. Sens. Actuators, B.

[cit129] Wang X., Liu Y., Su L., Wang W., Wang J. (2025). Magnetically boosted light-driven hydrogel microrobots for ultrasensitive and fast detection of PFOS distribution in unprocessed urine, bile, and whole blood. J. Hazard. Mater..

[cit130] MMcNeill J. M., Nama N., Braxton J. M., Thomas E. M. (2020). ACS Nano.

[cit131] Aghakhani A. (2022). *et al.*, High shear rate propulsion of acoustic microrobots in complex biological fluids. Sci. Adv..

[cit132] Ren L. (2019). *et al.*, 3D steerable, acoustically powered microswimmers for single-particle manipulation. Sci. Adv..

[cit133] Kaynak M., Dirix P., Sakar M. S. (2020). Addressable Acoustic Actuation of 3D Printed Soft Robotic Microsystems. Adv. Sci..

[cit134] Mohanty S., Lin Y., Paul A., van den Broek M. R. P., Segers T., Misra S. (2024). Acoustically Actuated Flow in Microrobots Powered by Axisymmetric Resonant Bubbles. Adv. Intell. Syst..

[cit135] Del Campo Fonseca A., Ahmed D. (2024). Ultrasound robotics for precision therapy. Adv. Drug Delivery Rev..

[cit136] Nikolov S. V., Shum H., Balazs A. C., Alexeev A. (2016). Computational design of microscopic swimmers and capsules: From directed motion to collective behavior. Curr. Opin. Colloid Interface Sci..

[cit137] Andhari S. S., Wavhale R. D., Dhobale K. D. (2020). *et al.*, Self-Propelling Targeted Magneto-Nanobots for Deep Tumor Penetration and pH-Responsive Intracellular Drug Delivery. Sci. Rep..

[cit138] Dixit A., Bag D. S., Sharma D. K., Eswara Prasad N. (2019). Synthesis of multifunctional high strength, highly swellable, stretchable and self-healable pH-responsive ionic double network hydrogels. Polym. Int..

[cit139] Cinay G. E., Erkoc P., Alipour M., Hashimoto Y., Sasaki Y., Akiyoshi K., Kizilel S. (2017). ACS Biomater. Sci. Eng..

[cit140] Zou B., Liu H., Fen X., Gao Z., Ge Z., Yang W. (2025). Design, optimization, and validation of a magnetic mother-child robot system for targeted drug deliveryAdvances in Transdisciplinary Engineering. Mater. Des..

[cit141] Lai Y. P., Li Z., Naguib H., Diller E. (2023). Hybrid Hydrogel-Magnet Actuators with pH-Responsive Hydrogels for Gastrointestinal Microrobots. Adv. Eng. Mater..

[cit142] Li H. (2016). et al. Smart Mater. Struct..

[cit143] Zhang D., Meng Y., Song Y., Cui P., Hu Z., Zheng X. (2022). Nanoscale.

[cit144] Chen X., Jin D., Hu Y., Yang L., Li R., Wang L., Ren Z., Wang D., Ji S., Hu K., Pan D., Wu H., Zhu W., Shen Z., Wang Y., Li J., Zhang L., Wu D., Chu J. (2021). ACS Nano.

[cit145] Yu Z., Li L., Mou F. (2023). *et al.*, Swarming magnetic photonic-crystal microrobots with on-the-fly visual pH detection and self-regulated drug delivery. InfoMat.

[cit146] Ryu K., Li G., Zhang K., Guan J., Long Y., Dong Z. (2025). Acc. Mater. Res..

[cit147] Zhan Z. (2022). et al. Int. J. Extrem. Manuf..

[cit148] Gu B., Cai J., Gong D., Zhou H., Peng G., Zhang D. (2025). ACS Appl. Mater. Interfaces.

[cit149] Xie T., Gao Y., Li Z., Gao W. (2024). J. Appl. Polym. Sci..

[cit150] Chen N., Zhou Y., Liu Y. (2022). *et al.*, Conductive photo-thermal responsive bifunctional hydrogel system with self-actuating and self-monitoring abilities. Nano Res..

[cit151] Wang Y. , Mu R., Ren H., Jia B., Gao X. and Sun C., Advances in Transdisciplinary Engineering, 2022, 10.3233/ATDE220424

[cit152] Wang Y., Chen W., Wang Z., Zhu Y., Zhao H., Wu K., Wu J., Zhang W., Zhang Q., Guo H., Ju H., Liu Y. (2023). Angew. Chem., Int. Ed..

[cit153] Liu Y., Zheng X. (2024). Bio-Inspired Double-Layered Hydrogel Robot with Fast Response via Thermo-Responsive Effect. Materials.

[cit154] Pilz da Cunha M., Foelen Y., van Raak R. J. H., Murphy J. N., Engels T. A. P., Debije M. G., Schenning A. P. H. J. (2019). Adv. Opt. Mater..

[cit155] Garcia-Torres J. , Hybrid Hydrogels with Stimuli-Responsive Properties to Electric and Magnetic Fields, Hydrogels - From Tradition to Innovative Platforms with Multiple Applications, IntechOpen, 2023, 10.5772/intechopen.102436

[cit156] Yoon D., Park S., Park S. (2023). Smart hydrogel structure for microbiome sampling in gastrointestinal tract. Sens. Actuators, B.

[cit157] Xianshuo W., Wu Q., Chen L., Sun Y., Lin C., Zhang C., Li S., Ma C., Jiang S. (2023). ACS Appl. Mater. Interfaces.

[cit158] Wang G., Wang S., Hu T., Shi F. (2024). Multifunctional Hydrogel with 3DPrintability, Fluorescence, Biodegradability, and Biocompatibility for Biomedical Microrobots. Molecules.

[cit159] Dong X., Yang Y., Bao Z., Midgley A. C., Li F., Dai S., Yang Z., Wang J., Liu L., Li W., Zheng Y., Liu S., Liu Y., Yu W., Liu J., Fan M., Zhu M., Shen Z., Gu X., Kong D. (2023). Micro-nanofiber composite biomimetic conduits promote long-gap peripheral nerve regeneration in canine models. Bioact. Mater..

[cit160] Ouyang J., Ma G., Wang Y., Ma Q., Guo X., Wu Y. (2025). Cost-effective sodium alginate-based hydrogel microrobot for multidirectional motion, high-efficiency Cd (II) adsorption and fluorescent detection. Carbohydr. Polym..

[cit161] Zhang H. (2021). *et al.*, Dual-responsive biohybrid neutrobots for active target delivery. Sci. Robot..

[cit162] Roy A., Manna K., Dey S., Chakraborty K., Dhara S., Pal S. (2025). Soft Matter.

[cit163] Li D., Liu C., Yang Y. (2020). *et al.*, Micro-rocket robot with all-optic actuating and tracking in blood. Light Sci. Appl..

[cit164] Sarker B., Li W., Zheng K., Detsch R., Aldo R. (2016). Boccaccini. ACS Biomater. Sci. Eng..

[cit165] Liu J., Du C., Chen H., Huang W., Lei Y. (2024). Nano-Micron Combined Hydrogel Microspheres: Novel Answer for Minimal Invasive Biomedical Applications. Macromol. Rapid Commun..

[cit166] Sun X., Zhang P., Ye Z., Li L., Qian L., Zhang H., Liu B., Lin G. (2023). ACS Biomater. Sci. Eng..

[cit167] Sun Z., Wang T., Wang J., Xu J., Tong S., Zhang T., Zhang B., Gao S., Zhao C., Yang M., Sheng F., Yu J., Hou Y. (2023). J. Am. Chem. Soc..

[cit168] Zhang B. (2023). *et al.*, Twin-bioengine self-adaptive micro/nanorobots using enzyme actuation and macrophage relay for gastrointestinal inflammation therapy. Sci. Adv..

[cit169] Wang J., Liu B., Wu E. Q., Ma J., Li P. (2024). Simulation Analysis of Deformation Control for Magnetic Soft Medical Robots. IEEE/CAA J. Autom. Sin..

[cit170] Das T., Sultana S. (2024). Multifaceted applications of micro/nanorobots in pharmaceutical drug delivery systems: a comprehensive review. Futur. J. Pharm. Sci..

[cit171] Massoud E. N., Hebert M. K., Siddharthan A., Ferreira T., Neron A., Goodrow M., Ferreira T. (2025). Delivery vehicles for light-mediated drug delivery: microspheres, microbots, and nanoparticles: a review. J. Drug Target..

[cit172] Shen J., Wang Y., Yao M., Liu S., Guo Z., Zhang L., Wang B. (2025). Long-span delivery of differentiable hybrid robots for restoration of neural connections. Matter.

[cit173] Hsiao J. H., Chang James J. Y., Cheng C. M. (2019). Soft medical robotics: clinical and biomedical applications, challenges, and future directions. Adv. Robot..

[cit174] Laurano R., Boffito M., Cassino C., Liberti F., Ciardelli G., Chiono V. (2023). Design of Injectable Bioartificial Hydrogels by Green Chemistry for Mini-Invasive Applications in the Biomedical or Aesthetic Medicine Fields. Gels.

[cit175] Chen Z., Wang Y., Chen H. (2024). *et al.*, A magnetic multi-layer soft robot for on-demand targeted adhesion. Nat. Commun..

[cit176] Singh N., Ali Md. A., Rai P., Ghori I., Sharma A., Malhotra B. D., John R. (2020). Lab Chip.

[cit177] Sokolov P., Samokhvalov P., Sukhanova A., Nabiev I. (2023). Biosensors Based on Inorganic Composite Fluorescent Hydrogels. Nanomaterials.

[cit178] Deng X., Su Y., Xu M., Gong D., Cai J., Akhter M., Chen K., Li S., Pan J., Gao C., Li D., Zhang W., Xu W. (2023). Magnetic Micro/nanorobots for biological detection and targeted delivery. Biosens. Bioelectron..

[cit179] Liu Z., Chen R., Wang H., Wang C., Zhang X., Yang Y., Pang W., Ren S., Yang J., Yang C., Li S., Zhou H., Gao Z. (2024). A colorimetric/electrochemical microfluidic biosensor using target-triggered DNA hydrogels for organophosphorus detection. Biosens. Bioelectron..

[cit180] Wang Q., Zhang L. (2021). ACS Nano.

[cit181] Zhou Y., Zhao Y., Zhao D. (2025). *et al.*, Sensing-actuating integrated asymmetric multilayer hydrogel muscle for soft robotics. Microsyst. Nanoeng..

[cit182] Guo S., Zhang S., Li H., Liu S., Koh J. J., Zhou M., Sun Z., Liu Y., Qu H., Yu Z., Zhang Y., Yang L., Chen W., He C., Lee C., Mao D., Ravi S. K., Lai Y., Tan S. C. (2025). Precisely manipulating polymer chain interactions for multifunctional hydrogels. Matter.

[cit183] Guo S., Zhang Y., Yu Z. (2025). *et al.*, Leaf-based energy harvesting and storage utilizing hygroscopic iron hydrogel for continuous power generation. Nat. Commun..

[cit184] Guo S. (2025). *et al.*, Self-powered green energy–harvesting and sensing interfaces based on hygroscopic gel and water-locking effects. Sci. Adv..

[cit185] Li J., Yu J. (2023). Biodegradable Microrobots and their Biomedical Applications: A Review. Nanomaterials.

[cit186] Tondera C., Hauser S., Krüger-Genge A., Jung F., Neffe A. T., Lendlein A., Klopfleisch R., Steinbach J., Neuber C., Pietzsch J. (2016). Gelatin-based Hydrogel Degradation and Tissue Interaction in vivo: Insights from Multimodal Preclinical Imaging in Immunocompetent Nude Mice. Theranostics.

[cit187] Madhusudana Rao K., Kumar A., Han S. S. (2018). Polysaccharide-based magnetically responsive polyelectrolyte hydrogels for tissue engineering applications. J. Mater. Sci. Technol..

[cit188] Bertsch P., Diba M., Mooney D. J., Leeuwenburgh S. C. G. (2023). Chem. Rev..

[cit189] He C., Yin M., Zhou H., Qin J., Wu S., Liu H., Yu X., Chen J., Zhang H., Lin Z., Wang Y. (2025). ACS Nano.

[cit190] Guo Y., Shan M., Suo L., Zhou Y., Wu S., Xie X., Sun D., Wang B., Wang Z., Horch R. E., Yang J., Sun J. (2025). Porous granular hydrogel scaffolds biofabricated from dual-crosslinked hydrogel microparticles for breast tissue engineering. Mater. Today Bio.

[cit191] Zhang J., Tang H., Wang H., Cai P., Gao Y., Guo X., Wang Y., Xuan F.-Z. (2025). Out-Of-Equilibrium Hydrogel Microrobots Exhibiting Autonomous Deformation, Controllable Autolysis, and Directed Locomotion. Small.

[cit192] Zhang H., Hua S., He C., Yin M., Qin J., Liu H., Zhou H., Wu S., Yu X., Jiang H., Wang Y., Qian Y. (2025). Application of 4D-Printed Magnetoresponsive FOGS Hydrogel Scaffolds in Auricular Cartilage Regeneration. Adv. Healthcare Mater..

[cit193] Liang J., Huang X., Qin K., Wei H., Yang J., Liu B., Fan Z. (2025). Implanted Magnetoelectric Bionic Cartilage Hydrogel. Adv. Mater..

[cit194] Lohani A., Saxena R., Duarte J. G., Khan S., Figueiras A., Mascarenhas-Melo F. (2025). Tailored polymeric hydrogels for regenerative medicine and drug delivery: From material design to clinical applications. Int. J. Pharm..

[cit195] Tuszynska M., Skopinska-Wisniewska J., Bartniak M., Bajek A. (2025). Bioconjugate Chem..

[cit196] Farasati Far B., Safaei M., Nahavandi R., Gholami A., Naimi-Jamal M. R., Tamang S., Ahn J. E., Ramezani Farani M., Suk Huh Y. (2024). ACS Omega.

[cit197] Zöller K., To D., Bernkop-Schnürch A. (2025). Biomedical applications of functional hydrogels: Innovative developments, relevant clinical trials and advanced products. Biomaterials.

[cit198] Yang Y., Liu Q., Wang M. (2024). *et al.*, Genetically programmable cell membrane-camouflaged nanoparticles for targeted combination therapy of colorectal cancer. Signal Transduction Targeted Ther..

[cit199] Zhao X., Chen W., Wu J., Shen Y., Xu B., Chen Z., Sun Y. (2025). Application of Biomimetic Cell Membrane-Coated Nanocarriers in Cardiovascular Diseases. Int. J. Nanomed..

